# London Dispersion Governs Stereochemistry, Stability, and Self-Sorting in a System of M_4_L_4_ Cages

**DOI:** 10.1021/jacs.6c05686

**Published:** 2026-05-21

**Authors:** Itai Massad, James T. F. Dobson, Paula C. P. Teeuwen, Tanya K. Ronson, Jonathan R. Nitschke

**Affiliations:** Yusuf Hamied Department of Chemistry, 2152 University of Cambridge, Cambridge CB2 1EW, U.K.

## Abstract

London dispersion forces between alkyl groups can give rise to “steric attraction”—a promising molecular design principle. The scope and magnitude of this attraction are subject to intense debate, however, along with its ability to outweigh competing effects. Here, we describe a system of four tetrahedral M_4_L_^R^4_ cages (M = Fe^II^ or Zn^II^, R = Me or Et), each confining 12 R groups within the cavity. These cages clearly demonstrate how steric attraction can dictate cage stereochemistry and stability—both in solution and in the gas phase—at the expense of other driving forces. The observed trends align with the optimization of London dispersion between the confined alkyl groups, predominating over criteria of steric hindrance, strain, solvophobic effects, and metal–ligand bond strength. These differences in stability manifested during the self-sorting of a mixture containing two metals and two ligands into cages that feature optimal alkyl-alkyl contacts, despite the entropic preference for a statistical mixture. This work thus establishes metal–organic cages as a promising platform to study London dispersion forces.

## Graphical abstract

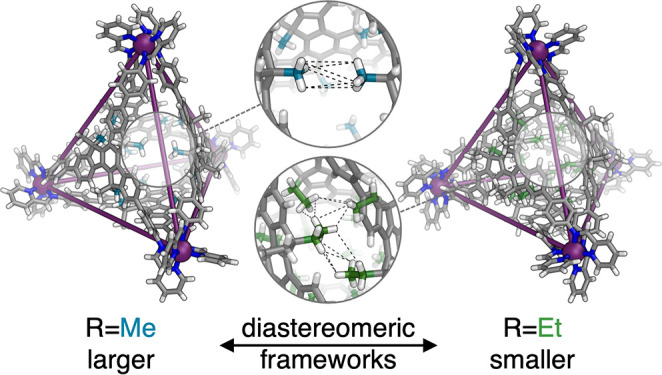

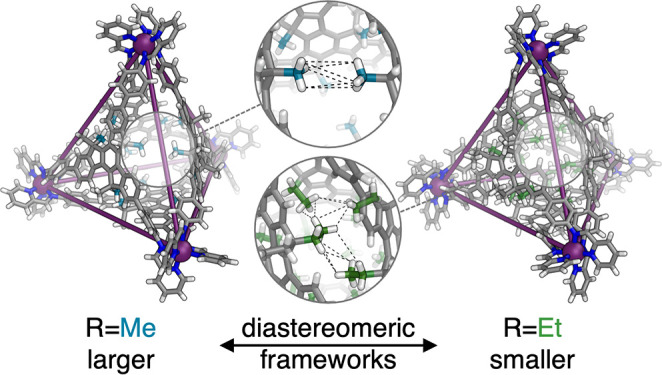

## Introduction

Steric hindrance is a core concept in organic chemistry, reflected in the notion that large substituents repel each other. However, as the distance between two substituents increases, the Pauli repulsion that underpins steric hindrance decays more rapidly than London dispersion^1^ (LD)—an attractive force that is usually neglected when considering steric effects.^2^ Indeed, LD can become decisive at intermediate distances,^3^ acting to bring together “bulky” groups.^4–6^ While LD is widely accepted to play a key role in the structure and energetics of biomolecules^7–10^ and supramolecular assemblies,^11,12^ it is not commonly invoked in the design of smaller organic assemblies. Recently, however, a growing number of examples^13^ is leading to increased interest in the application of LD in molecular design. Steric attraction^14^ through LD has been shown to stabilize otherwise labile or unfavored organic,^15^ organometallic,^16,17^ and inorganic structures,^18,19^ and even lead to contra-steric selectivity in organic reactions.^20–22^ The design of novel catalysts provides a particularly demanding testing ground, where the subtle modulation of competing energy barriers is key to attaining high selectivity. Recent successes in catalyst design^23–30^ bode well for the adoption of LD as a design element in organic and supramolecular chemistry.

To gain confidence in employing dispersion in molecular design, it must be tested and quantified under controlled conditions, so that the magnitude and scope of LD interactions can be weighed against competing effects. Systems that study LD^31^ are therefore valuable, but challenging to design, as they must fulfill several criteria: they should be structurally well-defined such that the distance and geometry of the interaction is unambiguous; address different substituents, with sizes ranging from small to large; and be measurable using analytical techniques sensitive enough to quantify these subtle interactions. Furthermore, information on the behavior of these systems both in solution and in the gas phase is required,^32^ to separate the contribution of LD from solvent competition^33–35^ and solvophobic effects,^36–39^ which either attenuate or amplify apparent dispersive attraction.^40,41^

Here, we show that self-assembled M_4_L_4_ coordination cages^42^ that constrict alkyl groups within their cavities serve as a promising platform to investigate LD. These well-defined tetrahedral cage frameworks minimize solvent competition, and their high symmetry leads to the inclusion of many instances of the same interaction, multiplying effects. The alkyl groups can be varied, and the distances between them can be tuned by changing the metal ions at the vertices. The cages were studied in the crystal, in solution, and in the gas phase, and their overall stability trends were correlated with the aggregate of the driving forces in the system. We found that our series of cages displayed distinct trends that defy canonical driving forces, instead tracking with the optimization of LD between the confined alkyl groups. Thus, this system is relevant both for fundamental studies of dispersion interactions, and the elucidation of their scope.

## Results and Discussion

We prepared four tetrahedral **M**_4_ **L** ^**R**^_4_ cages (Figure 1a), assembled from hexaalkylated truxene-based trianilines **A^Me^** or **A^Et^**, 2-formylpyridine, and either Fe­(NTf_2_)_2_ or Zn­(NTf_2_)_2_. Each ligand projects three R groups into the cavity, yielding 12 proximal R groups.

**Figure 1:**
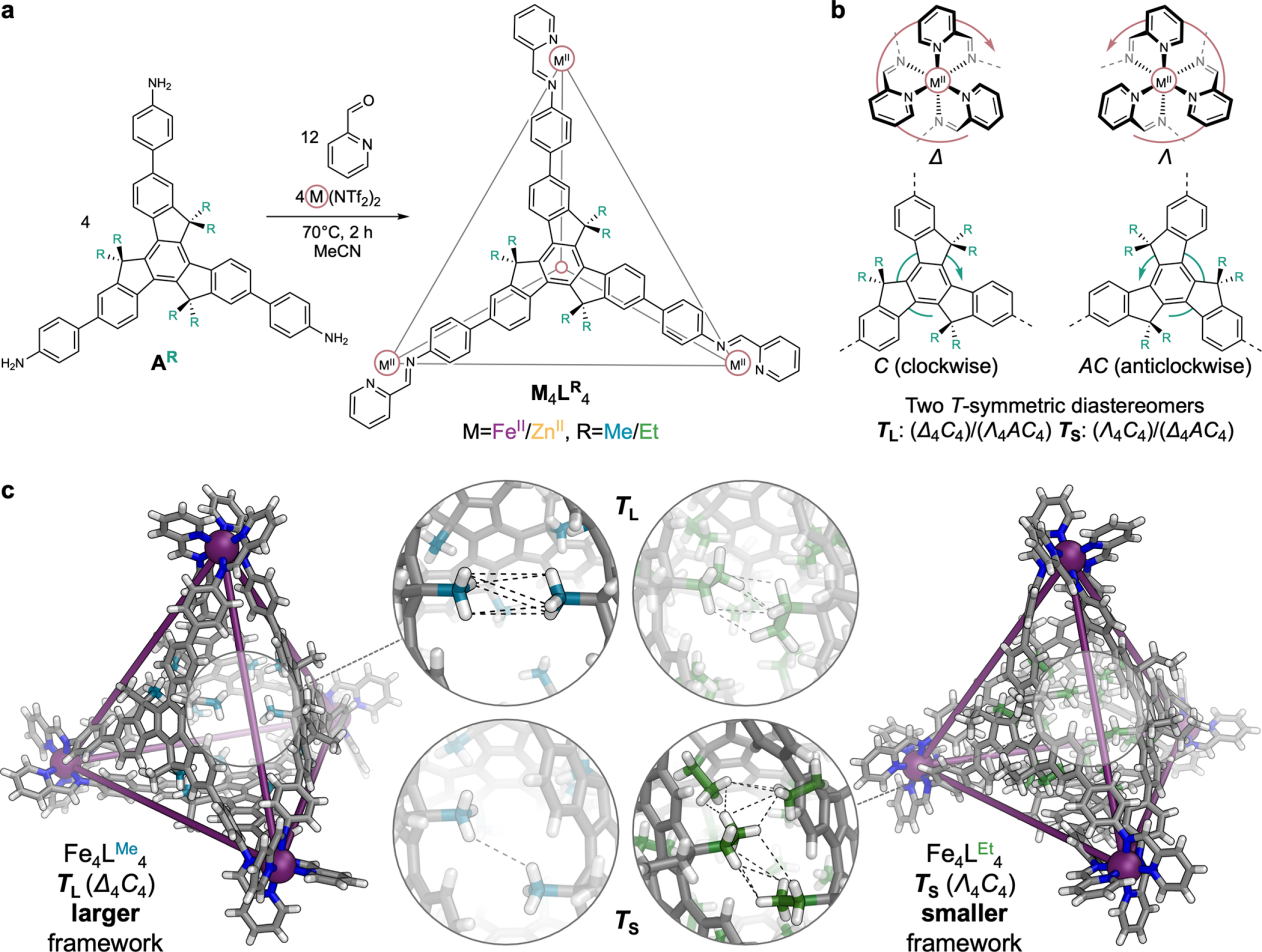
Structure and stereochemistry of the **M**_4_ **L** ^**R**^_4_ cages. (a) Subcomponent self-assembly of **M**_4_ **L** ^**R**^_4_ cages. (b) Two types of stereogenic elements in the cage give rise to two distinct *T*-symmetric diastereomers. (c) The crystal structures of **Fe**_4_ **L^Me^**_4_ and **Fe**_4_ **L^Et^**_4_ revealed diastereomeric cage frameworks. The interaction between the endohedral alkyl groups is highlighted in the center, with H–H contacts below 3.2 Å shown as dashed lines. The conformations of the methyl groups were determined from the electron density maps, and the C–H bond lengths were fixed to standard values. The MM3-optimized models in the faded circles, depicting the disfavored diastereomers, were obtained by swapping the alkyl groups in the crystal structures.

### Stereochemistry of M_4_L^Me^_4_ and M_4_L^Et^_4_

Although both **Fe**_4_ **L^Et^**_4_ and **Fe**_4_ **L^Me^**_4_ adopted configurations with chiral tetrahedral (*T*) point symmetry, their structures differ notably (Figure 1c). **Fe**_4_ **L^Et^**_4_ formed a structure with *Λ*_4_ *C*_4_ relative stereochemistry between the metal vertices and the truxene faces (Figure 1b),^43–46^ a configuration we label ***T*_S_** (for tetrahedral, small; see below). The stereochemistry of **Fe**_4_ **L^Et^**_4_ matches its known **Zn**_4_ **L^Et^**_4_ congener.^47^ **Fe**_4_ **L^Me^**_4_ and **Zn**_4_ **L^Me^**_4_, in contrast, adopted the relative configuration *Δ*_4_ *C*_4_, labeled ***T*_L_** (for tetrahedral, large; see below). We note that the absolute stereochemistry referred to throughout the paper (e.g., *Λ*_4_ *C*_4_ rather than *Δ*_4_ *AC*_4_ for ***T*_*S*_**) is arbitrary, since all cages are formed as racemates.

Computational studies (Supporting Information, Section 7) indicated that for the parent structure **Zn**_4_ **L** ^**H**^_4_, with no alkyl groups, the *Λ*_4_ *C*_4_ configuration of the ***T*_S_** diastereomer is lower in energy than the ***T*_L_** diastereomer.^48^ Thus, **Fe**_4_ **L^Et^**_4_ adopted the ***T*_S_** configuration, featuring a minimally strained backbone, while **Fe**_4_ **L^Me^**_4_—although the less sterically demanding Me groups could fit in place of the Et groups in the ***T***_**S**_ isomer—preferred the more strained ***T*_L_** stereochemistry. Moreover, the ***T***_**L**_ framework favored by **Fe**_4_ **L^Me^**_4_ is larger than the ***T***_**S**_ framework favored by **Fe**_4_ **L^Et^**_4_—the larger alkyl group (Et) forms the smaller framework (***T***_**S**_), and vice versa. The system thus displays contra-steric stereoselectivity.

The size difference between the two cages is manifested in three key structural parameters (Supporting Information, Section 6): First, the average Fe–Fe distance in **Fe**_4_ **L^Me^**_4_ is 0.37 Å longer than in **Fe**_4_ **L^Et^**_4_ (21.66 vs 21.29 Å, respectively). We interpret this slight variation in Fe–Fe distance to reflect the minor intrinsic size difference between the ***T*_S_** and ***T*_L_** frameworks. Second, the average distance between a truxene centroid and the center of the cage (defined as the centroid between the four metals) is 0.50 Å longer in **Fe**_4_ **L^Me^**_4_ than in **Fe**_4_ **L^Et^**_4_ (5.94 vs 5.44 Å, respectively). Third, for each R, the calculated cavity volume^49^ is larger for ***T*_L_** than ***T*_*S*_**: 656 vs 392 Å^3^ for **Fe**_4_ **L^Me^**_4_ and 211 vs 61 Å^3^ for **Fe**_4_ **L^Et^**_4_ (Table S6, Figure S51). The larger cavity size of ***T*_L_**-**Fe**_4_ **L^Me^**_4_ should also disfavor its formation based on the solvophobic effect, which drives structures to minimize their cavity size,^50^ and with it, the number of ordered encapsulated solvent molecules.

Inspection of the methyl–methyl contacts in its crystal structure (Figure 1c, center) suggests an explanation for the counterintuitive stereochemical preference of **Fe**_4_ **L^Me^**_4_: in ***T*_L_**-**Fe**_4_ **L^Me^**_4_, the two Me groups at each edge of the tetrahedron approach in a staggered head-to-head orientation, with six close H–H contacts, whereas the alternative ***T*_*S*_** diastereomer (modeled based on the crystal structure of **Fe**_4_ **L^Et^**_4_) positions the Me groups side-to-side, with only one side-on H–H contact per edge. In the observed ***T*_L_** diastereomer, the orientation and distance between methyl groups closely resemble the computed structure of the methane dimer,^51,52^ where maximization of LD leads to a similar 3:3 staggered orientation. Specifically, the average H–H distance between neighboring Me groups in **Fe**_4_ **L^Me^**_4_ is 3.18 Å, and 3.05 Å in **Zn**_4_ **L^Me^**_4_ compared to 3.17 Å in the 3:3 staggered methane dimer.^53^ These rare head-to-head Me-Me contacts are expected to be much more favorable than more common side-on Me-Me contacts, and further bolstered by the polarizable truxene backbone the Me groups are attached to.^53^

Conversely, in **Fe**_4_ **L^Et^**_4_, the more compact ***T*_S_** framework allows the Et groups to form a dense network of 61 close (2.34–3.20 Å) H–H contacts (58 contacts in **Zn**_4_ **L^Et^**_4_), which again match the optimal distance range reported for LD between alkyl groups. Each Et group interacts with three neighboring Et groups: one at the same edge, and two that are placed further away along the cage backbone but reside near the same vertex. Models indicate that the larger ***T*_L_**-**Fe**_4_ **L^Et^**_4_ diastereomer would only feature one pair of interacting Et groups at each edge. Furthermore, due to the head-to-head arrangement of the Et groups in ***T*_L_**-**Fe**_4_ **L^Et^**_4_, some of the Et hydrogens at the edges sterically repel rather than attract as they are <2.0 Å apart, which would lead the cage to distort in order to relieve this repulsion, thus introducing strain in the cage backbone and disfavoring this diastereomer. Solvophobic effects would also disfavor ***T*_L_**-**Fe**_4_ **L^Et^**_4_ relative to ***T*_S_**-**Fe**_4_ **L** ^**Et**^_4_, due to its larger central cavity, and additional “pockets” between each metal vertex and the three adjacent Et groups.

Overall, the relative stereochemistry displayed by **Fe**_4_ **L^Me^**_4_ and **Zn**_4_ **L^Me^**_4_ defies both the expected intrinsic stereochemical preference of the cage backbone and solvophobic effects, each anticipated to steer the system away from the more strained and voluminous ***T*_L_** diastereomer. Remarkably, LD between six pairs of methyl groups overcame both effects.

### Alkyl-Dependent Spin-State Preference in Fe_4_L^R^_4_

In solution, both the colors and ^1^H NMR spectra of **Fe**_4_ **L^Me^**_4_ and **Fe**_4_ **L^Et^**_4_ indicated distinct properties (Figure 2a): **Fe**_4_ **L^Me^**_4_ was deep violet in acetonitrile solution, and its ^1^H NMR spectrum displayed a single set of sharp signals between 1.6 and 10.3 ppm, consistent with a *T*-symmetric structure containing diamagnetic, low-spin Fe^II^. In contrast, reddish-brown **Fe**_4_ **L^Et^**_4_ displayed broad signals between −1.3 and 46.8 ppm, consistent with a proportion of paramagnetic, high-spin Fe^II^.

**Figure 2:**
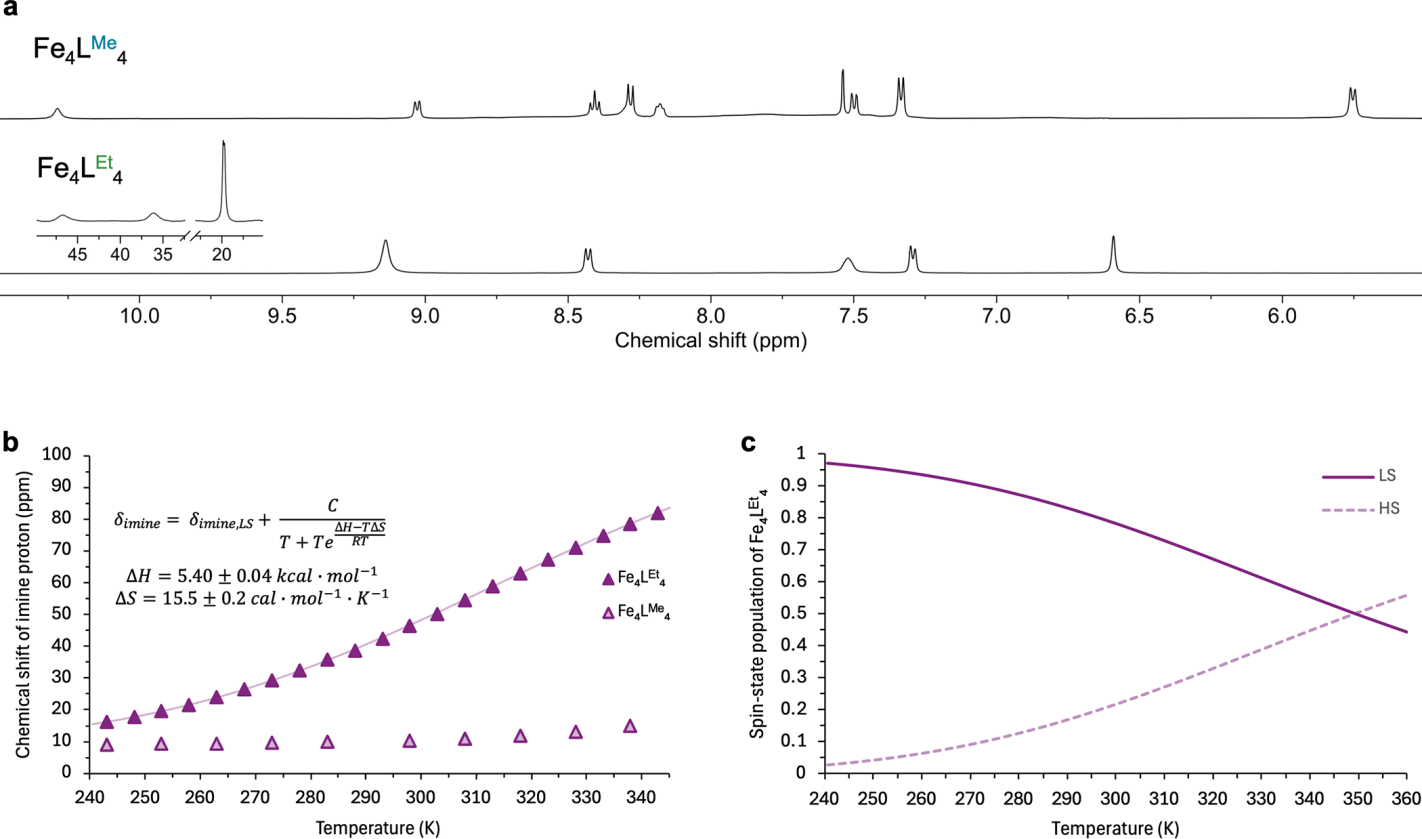
Divergent magnetic behaviors of **Fe**_4_ **L^Me^**_4_ and **Fe**_4_ **L^Et^**_4_. (a) The aromatic and imine region of the ^1^H NMR spectrum (CD_3_CN, 298 K) of **Fe**_4_ **L^Me^**_4_ (top) extends to 10.3 ppm, whereas **Fe**_4_ **L^Et^**_4_ (bottom) displays peaks up to 46.8 ppm, indicating paramagnetic character. (b) Chemical shifts of the imine protons of **Fe**_4_ **L^Et^**_4_ and **Fe**_4_ **L^Me^**_4_ at different temperatures. The strong temperature sensitivity observed for **Fe**_4_ **L^Et^**_4_ is indicative of spin-crossover. (c) Calculated spin-state populations for **Fe**_4_ **L^Et^**_4_ as a function of temperature.

The ^1^H chemical shifts of **Fe**_4_ **L^Et^**_4_ were sensitive to temperature, indicating temperature-dependent spin-crossover (Figure 2b, Figures S46–S47).^54^ The ΔH and ΔS values for the spin-crossover equilibrium were extracted by fitting chemical shift as a function of temperature to the equation shown in Figure 2b,^55^ which enabled the population of high- and low-spin states to be calculated at each temperature (Figure 2c and Supporting Information, Section 4). At room temperature, **Fe**_4_ **L^Et^**_4_ is 21% high-spin, reaching 50% high-spin at 349 K.

Strong-field pyridine-imine ligands give rise to low-spin Fe^II^ cages in many reported examples.^42^ To stabilize high-spin Fe^II^ within cages, weaker-field imine ligands must be employed, which incorporate sterically demanding 6-methyl-2-formylpyridine,^56^ or other aldehydes that contain five-membered nitrogen heterocycles.^57–59^ We were therefore surprised that tuning of the alkyl groups far from the Fe^II^ centers led to marked differences in spin-state preference. A recent study on an octahedral Fe^II^ cage showed that guest encapsulation can stabilize the low-spin state of the cage, in line with shrinking of the cavity to maximize attractive contacts with the guest.^60^ In contrast, the observed stabilization of the high-spin state of **Fe**_4_ **L^R^**_4_ upon increasing the size of the endohedral alkyl groups from Me to Et indicates steric congestion between the Et groups in **Fe**_4_ **L^Et^**_4_, which is relieved by elongating the Fe–N bonds at the vertices. This elongation in turn weakens the effective ligand field and favors the high-spin configuration at the Fe^II^ vertices.

The distinct spin-state preferences of **Fe**_4_ **L^Me^**_4_ and **Fe**_4_ **L^Et^**_4_ provide a benchmark for the influence of LD on thermodynamic stability. While the partial high-spin character of **Fe**_4_ **L^Et^**_4_ would be expected to destabilize it compared to **Fe**_4_ **L^Me^**_4_, due to the weaker bonds and smaller crystal-field stabilization energy at the Fe^II^ vertices, increased alkyl–alkyl contacts between the Et groups in **Fe**_4_ **L^Et^**_4_ compared to the Me groups in **Fe**_4_ **L^Me^**_4_ would oppose this effect and stabilize **Fe**_4_ **L^Et^**_4_. The overall relative stability of the two cages would thus report on the balance between these two competing effects (see below).

### Competition Experiments: Relative Stabilities in Solution

The diverse behaviors of the four cages in the solid state and in solution prompted us to study their relative stabilities, in order to gauge whether LD influences it significantly. Competition experiments provide a simple means to compare the solution-state stabilities of the cages (Figure 3).^61^ To compare the effects of the two alkyl groups, a mixture of aniline subcomponents **A^Me^** and **A^Et^** (4 equiv each) was treated with limiting amounts of 2-formylpyridine (12 equiv) and either Zn^II^ or Fe^II^ (4 equiv). At equilibrium, the most stable cage should form, leaving the remaining subcomponent(s) unreacted. In the case of no preference for either R group, a statistical mixture of cages incorporating **L^Me^** and **L^Et^** would result. In practice (Figure 3a), both Fe^II^ and Zn^II^ were observed to exclusively form **M**_4_ **L^Et^**_4_, as confirmed by ^1^H NMR and high-resolution mass spectrometry (HRMS, Figures S54, S58). The thermodynamic nature of this selectivity was verified through two additional experiments: for both M = Fe and Zn, when **M**_4_ **L^Me^**_4_ was treated with 4 equiv of **A^Et^**, complete subcomponent exchange occurred to afford **M**_4_ **L^Et^**_4_ (Figures S55, S59), whereas adding **A^Me^** to **M**_4_ **L^Et^**_4_ left the cage unchanged (Figures S56, S60).

**Figure 3:**
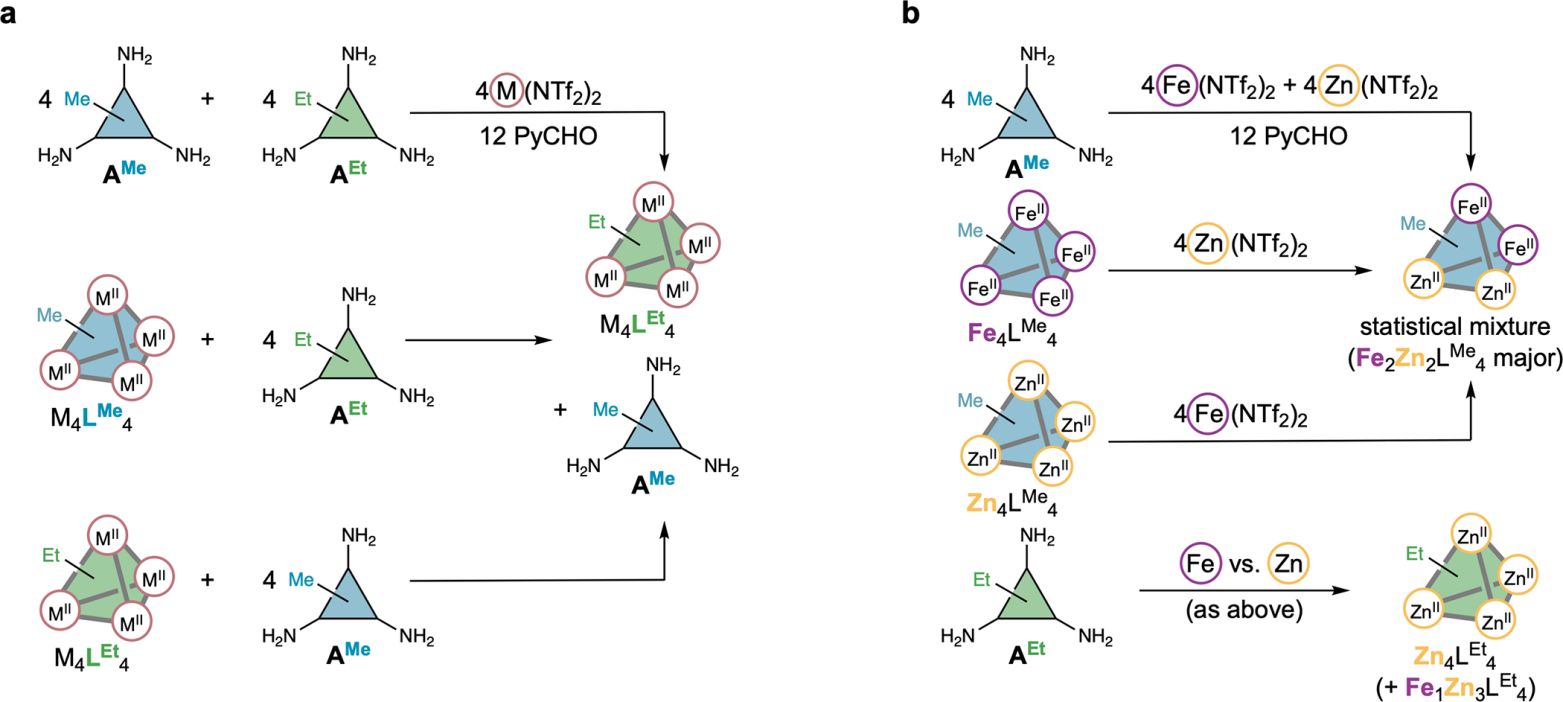
Competition experiments. (a) Alkyl competition: the reaction of an equimolar mixture of **A^Me^** and **A^Et^** with limiting amounts of either Fe^II^ or Zn^II^ and 2-formylpyridine led to the exclusive formation of **M**_4_ **L^Et^**_4_. The thermodynamic nature of the selectivity is confirmed by subcomponent substitution reactions: adding **A^Et^** to preformed **M**_4_ **L^Me^**_4_ led to complete displacement, and the addition of **A^Me^** left **M**_4_ **L^Et^**_4_ unchanged. (b) Metal competition: cages ordinarily incorporate Fe^II^ over Zn^II^, due to the stronger Fe^II^–N bonds (Supporting Information, Section 8). In contrast, both the Me and Et cages incorporate apparently incommensurate amounts of Zn^II^: **M**_4_ **L^Me^**_4_ affords a roughly statistical mixture, whereas **M**_4_ **L^Et^**_4_ incorporates Zn^II^ selectively. PyCHO, 2-formylpyridine.

This selectivity could reflect stabilization of **M**_4_ **L^Et^**_4_ relative to **M**_4_ **L^Me^**_4_ either through stronger LD between the larger Et groups, solvophobic preference for the smaller cavity of **M**_4_ **L^Et^**_4_, or a combination of both effects. In the case of Fe^II^, the selectivity for partially high-spin **Fe**_4_ **L^Et^**_4_ (46% high-spin at 70 °C) over low-spin **Fe**_4_ **L^Me^**_4_ is noteworthy, since a preference for low-spin Fe^II^ cages has been shown to drive transformations between cages.^56^ In the present system, this effect appears to be overcome by the stabilization provided by the Et groups in **Fe**_4_ **L^Et^**_4_.

Analogous competition experiments were performed to compare the stabilities of the Fe- and Zn-cages (Figure 3b). First, control experiments using a known triazine-based trianiline^62^ and 2-formylpyridine were carried out (Figures S61–S65). Selective formation of the low-spin Fe_4_L_4_ cage was observed, leaving unreacted Zn^II^ in acetonitrile solution. As expected from well-established crystal-field stabilization energy and bond-dissociation energy trends,^63^ Fe^II^ was exclusively incorporated into the cage vertices over Zn^II^.

In contrast, the ligands derived from both truxene subcomponents **A^Me^** and **A^Et^** displayed high affinity for Zn^II^. **A^Me^** formed a near-statistical mixture of mixed-metal cages, with **Fe**_2_ **Zn**_2_ **L^Me^**_4_ as the principal species detected by HRMS (Figures S66–S69). Remarkably, **A^Et^** showed a strong preference for Zn^II^, affording **Zn**_4_ **L^Et^**_4_ and **Fe**_1_ **Zn**_3_ **L^Et^**_4_ as the major products, with only trace amounts of **Fe**_4_ **L^Et^**_4_ detected by HRMS (Figures S70–S73). Incorporation of Zn^II^ into the cage vertices in place of Fe^II^ leaves the unreacted Fe^II^ coordinated to MeCN, a much weaker-field ligand, thus incurring the loss of the larger crystal-field stabilization energy and stronger bonds between Fe^II^ and the pyridine-imine ligands. A competing effect must therefore outweigh the stabilization that Fe^II^ incorporation would bring. Avoidance of strain might lead the cages to favor Zn^II^ due to its more flexible coordination geometry. However, no significant deviation from octahedral geometry around the metal centers of the two Fe^II^ cages was observed (Supporting Information, Section 6). Similarly, comparing **Fe**_4_ **L^Me^**_4_ to **Zn**_4_ **L^Me^**_4_ (and **Fe**_4_ **L^Et^**_4_ to **Zn**_4_ **L^Et^**_4_) revealed no significant distortions to the organic backbones of the Fe^II^ cages.

Rather than differences in backbone strain, we infer that differences between the Me-Me contacts in **Fe**_4_ **L^Me^**_4_ and **Zn**_4_ **L^Me^**_4_ contribute instrumentally to the observed affinity for Zn^II^. There are 34 close (<3.2 Å) H–H contacts in **Zn**_4_ **L^Me^**_4_, as compared to 16 in **Fe**_4_ **L^Me^**_4_. The Me-Me contacts at the cage edges are also more uniform in geometry and distance in **Zn**_4_ **L^Me^**_4_. Although we did not identify any obvious signs of strain in the crystal structures of either **Fe**_4_ **L^Et^**_4_ or **Zn**_4_ **L^Et^**_4_, we hypothesize that the strain signaled by the spin-crossover behavior of **Fe**_4_ **L^Et^**_4_ (Figure 2) is effectively dissipated in the Zn-congener, allowing for a less strained backbone and optimal Et-Et contacts. Thus, we conclude that the surprising affinity for Zn^II^ results from the maximization of attractive alkyl–alkyl contacts, which outweigh the preference to incorporate Fe^II^ in the strong-field ligand environment of the cage vertices.

### CID-MS: Relative Stabilities in the Gas Phase

Some of the above solution-state stability trends might be ascribed to solvophobic effects rather than LD, and separating the effects of the two driving forces is not straightforward.^65^ We were therefore keen to compare the stabilities of the four cages in the gas phase, in the absence of solvent. Collision-induced dissociation mass spectrometry (CID-MS) has been extensively employed to compare and quantify the gas-phase stability of inorganic,^63^ organometallic,^66^ and supramolecular^67–71^ structures, bringing dispersion effects into sharp relief.^33,72,73^

In the CID-MS experiment, the [(**M**_4_ **L** ^**R**^_4_)­(NTf_2_)_1_]^7+^ ion of each cage is isolated in the quadrupole and subsequently exposed to increasingly higher voltages in the trap-cell. This procedure progressively increases the center-of-mass collision energy and thus the extent of fragmentation following cleavage of the metal–ligand bonds through CID (Figure 4a, Figures S75, S76). The different cages fragment to varying extents at a given trap-cell voltage, providing a basis for comparing their stability. In contrast to methods which evaluate stability in the bulk solid or solution, CID-MS gauges the stability of individual molecules without the interference of intermolecular interactions. It is therefore an attractive method to gauge the contribution of LD between the confined alkyl groups to the overall stability of the cages.

**Figure 4:**
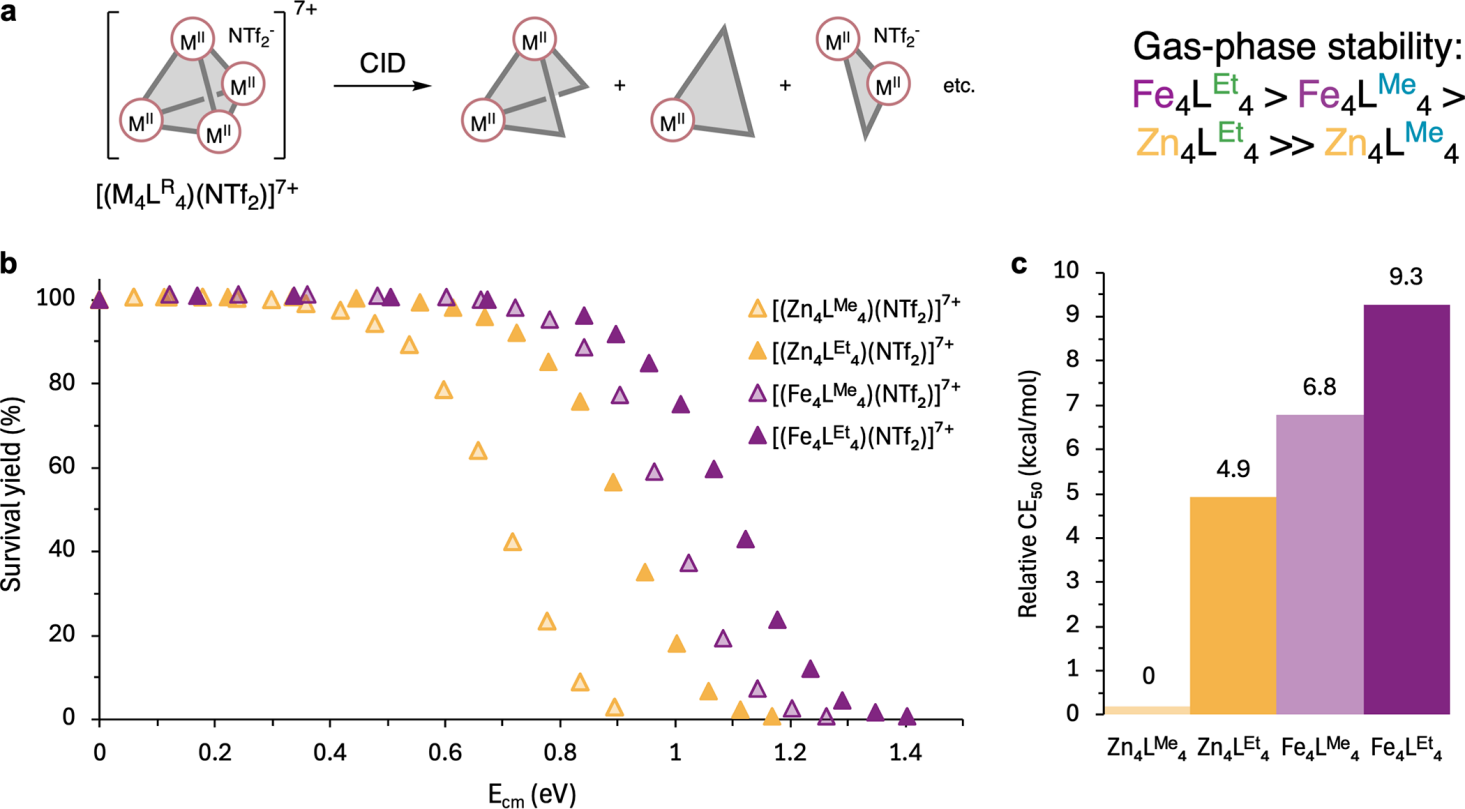
CID-MS and gas-phase stability trends. (a) Schematic description of the gas-phase fragmentation of the cages during collision-induced dissociation. (b) Breakdown curves for the 7+ ions of the four cages. Survival yield indicates the percentage of intact cage, and E_cm_ is the kinetic energy in the center-of-mass frame. (c) Relative CE_50_ values (the energy required for 50% fragmentation) interpolated from the breakdown curves of the 7+ ions provide a quantitative metric to compare the stabilities of the cages.

Figure 4b shows the breakdown curves for the four cages, where the survival yield (%) is plotted against the center-of-mass collision energy (E_cm_) (Supporting Information, Section 9). A clear qualitative stability trend emerged: **Fe**_4_ **L^Et^**_4_ > **Fe**_4_ **L^Me^**_4_ > **Zn**_4_ **L^Et^**_4_ > **Zn**_4_ **L^Me^**_4_, from most to least stable. Both Fe^II^ cages are more stable than either Zn^II^ cage, reflecting the stronger Fe^II^–ligand bonds,^63^ and each Et-cage is more stable than its Me congener. The retention of the **M**_4_ **L^Et^**_4_ > **M**_4_ **L^Me^**_4_ stability trend in the gas phase demonstrates that London dispersion does indeed contribute to the stability trends observed in solution, rather than solvophobic effects alone.

The greater gas-phase stability of the Fe^II^ cages compared to the Zn^II^ cages observed by CID-MS seemingly contrasts with the preference for Zn^II^ cages observed in the competition experiments described above. However, the CID-MS experiments directly compared bond strengths at the vertices of the different cages, whereas the competition experiments tested the overall stability of the system, including both the cage and the unbound metal ions that remain in solution. While the acetonitrile solvent does not bind to the coordinatively saturated hexacoordinate metal ions at the cage vertices, it coordinates to and stabilizes the excess metal ions remaining in solution. We would therefore expect analogous competition experiments between Fe^II^ and Zn^II^ in a weakly coordinating solvent to lead to an outcome in line with the CID-MS experiments, where Fe^II^, featuring the stronger bonds, is incorporated exclusively. Limited solubility of the cages in such solvents precluded these experiments, however.

By comparing the energy values at which 50% fragmentation is observed (CE_50_, Figure 4c), interpolated from the breakdown curves (Figures S74–S75),^74^ this gas-phase stability trend was quantified. A remarkably large 4.9 kcal·mol^1^ difference in stability between **Zn**_4_ **L^Me^**_4_ and **Zn**_4_ **L^Et^**_4_ was thus observed. This magnitude of energy difference is more than sufficient to induce the observed complete selectivity for the formation of the latter in solution (Figure 3a), signaling that dispersion is likely to dominate the system. Differentiating between Me and Et to achieve high selectivity is considered a “holy grail” in catalysis;^75–78^ the stability difference observed here shows that LD is capable of doing so.

The 2.5 kcal·mol^–1^ difference in CE_50_ values between **Fe**_4_ **L^Et^**_4_ and **Fe**_4_ **L^Me^**_4_ is also noteworthy, since the partial high-spin character of **Fe**_4_ **L^Et^**_4_ is expected to weaken the Fe^II^–N bonds holding it together compared to low-spin **Fe**_4_ **L^Me^**_4_, leading to an opposite stability trend. Stronger LD between the Et groups thus overrules the effect of the intrinsically weaker Fe–N bonds, resulting in greater stability of **Fe**_4_ **L^Et^**_4_. Since **Zn**_4_ **L^Me^**_4_ and **Fe**_4_ **L^Me^**_4_ favor the ***T*_L_** over the ***T*_S_** configuration, we note that our CID-MS experiments underestimate the more direct comparison between the stability of ***T*_S_**-**M**_4_ **L^Me^**_4_ and ***T***_**S**_-**M**_4_ **L^Et^**_4_, which would feature the same backbone configuration with only the alkyl groups varied (provided that the preference for ***T*_L_**-**M**_4_ **L^Me^**_4_ over ***T*_S_**-**M**_4_ **L^Me^**_4_ is retained in the gas phase).

Comparison of the gas-phase stabilities of the four cages thus indicates that LD can account for the stability trends observed in solution, and even outweigh changes to the strength of the M–N bonds at the vertices.

Direct computational quantification of LD interactions within the cage cavity proved challenging due to the size of the cages and the large number of H–H contacts, which could lead to the accumulation of otherwise small errors. Subsequent CID-MS experiments on series of cages encapsulating homologous alkyl groups could prove useful as a benchmark for computational tools that estimate the strength of alkyl–alkyl LD.

### Self-Sorting

In light of the stability trends observed in solution and in the gas phase, we tested the self-sorting^79^ of a mixture containing **A^Me^**, **A^Et^**, Fe^II^ and Zn^II^ (Figure 5a), which could give rise to 25 different cage compositions: five combinations of Fe^II^ and Zn^II^, together with five combinations of **L^Me^** and **L^Et^** (Supporting Information, Section 10). The balance between the entropic driving force for mixed cage formation (Figure 5b, left) and the enthalpic favorability of certain metal–alkyl combinations would serve as an additional gauge of the scope of LD control over the system.

**Figure 5:**
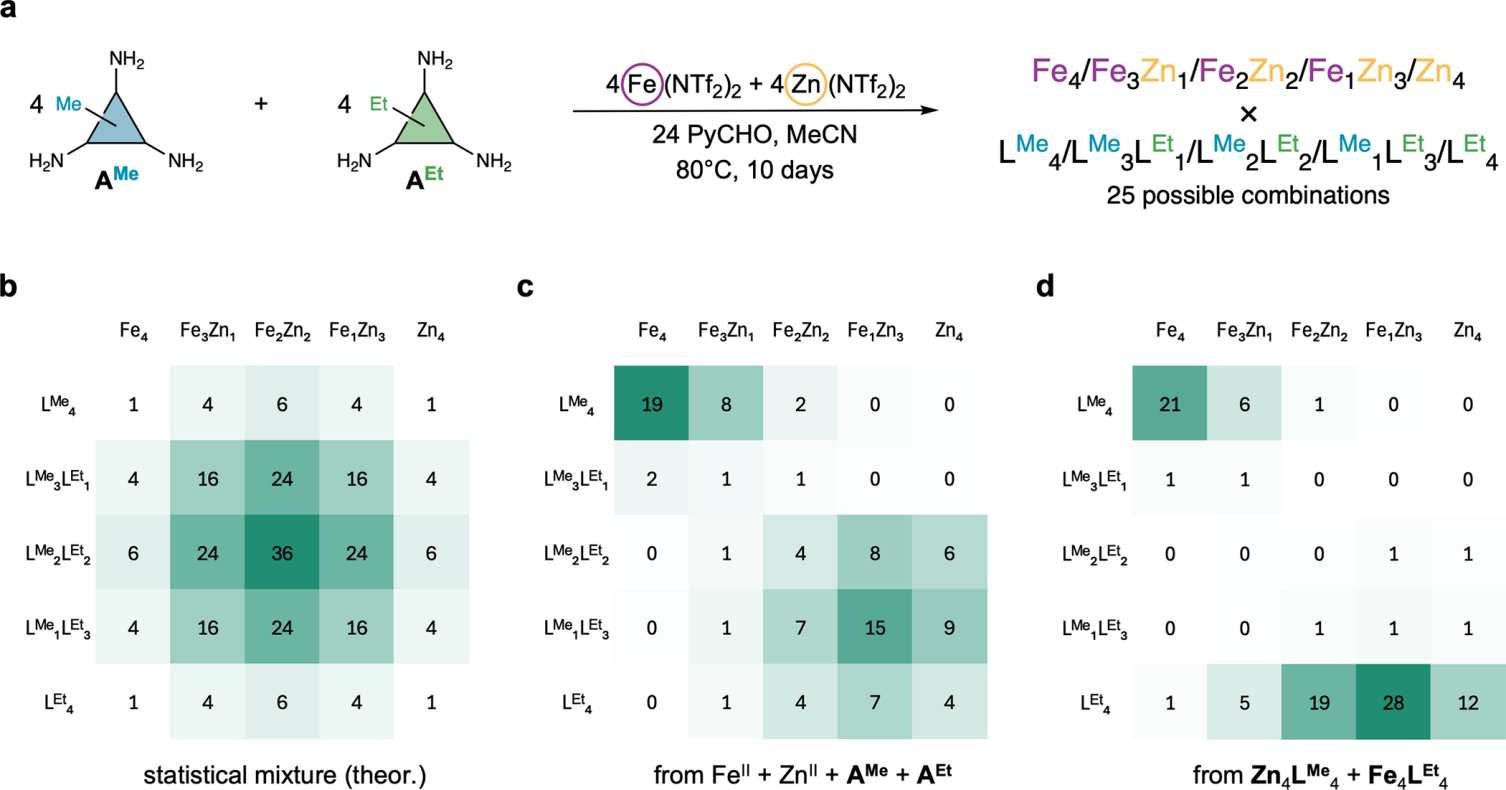
Self-sorting among cages. (a) The mixture of 25 possible cages containing **A^Me^**, **A^Et^**, Fe^II^, and Zn^II^, wherein mixed cages are entropically favored. (b–d) Heat maps depicting selectivity in the above mixture: (b) a hypothetical, purely statistical mixture. Relative concentrations were normalized relative to the least statistically favored homoleptic and homometallic cages; (c) product distribution after 10 days of reaction at 80 °C, starting from subcomponents or (d) from the mismatched cages **Zn**_4_ **L^Me^**_4_ and **Fe**_4_ **L^Et^**_4_. Relative concentrations were determined by HRMS (Supporting Information, Section 10) and are presented on a percentage scale. PyCHO, 2-formylpyridine.

Initially, the above mixture afforded a kinetically trapped set of products, where mixed-alkyl cages such as **Zn**_4_ **L^Me^**_2_ **L^Et^**_2_ predominate (Figure S78). Subsequent equilibration of this mixture into the distribution shown in Figure 5c required prolonged heating and proceeded on a longer time scale than previously reported examples of cage ligand-exchange processes.^80^ We attribute this kinetic barrier to the stabilization of cages featuring two or more **L^Et^** panels through LD and solvophobic effects, which inhibits their disassembly. In a control experiment, heating an equimolar mixture of **Zn**_4_ **L^Me^**_4_ and **Fe**_4_ **L^Et^**_4_ (or **Fe**_4_ **L^Me^**_4_ and **Zn**_4_ **L^Et^**_4_, Figure S81) produced a distribution of products that self-sorted more effectively (Figure 5d), in which the metal ions swapped to match Fe^II^ with **L^Me^** and Zn^II^ with **L^Et^**, and where the alkyl group distribution favored homoleptic cages (**M**_4_ **L^Me^**_4_ and **M**_4_ **L^Et^**_4_) almost exclusively. At equilibrium, the product distributions from both experiments should converge, given that the individual components present in system are identical. Extended reaction times led to precipitation of the cages, however, which precluded closer approach to equilibrium. Thus, the experiments starting from the individual subcomponents (Figure 5c) and the preformed cages (Figure 5d) represent kinetically trapped systems that provide lower and upper bounds for the equilibrium distribution, respectively. The higher stability of **M**_4_ **L^Et^**_4_ cages, and the observation that the product distribution in the latter experiment changes more slowly than in the former case, suggest that the true equilibrium features predominantly homoleptic cages (**M**_4_ **L^Me^**_4_ and **M**_4_ **L^Et^**_4_). Nonetheless, despite this ambiguity, both empirical distributions (Figure 5c,d) indicate that **Fe**_4_ **L^Me^**_4_ and **Zn**_4_ **L^Et^**_4_ are enthalpically favored (Figure S83).

We thus draw the qualitative conclusion that the apparently minor structural differences between cages in this system drive enthalpic differences of sufficient magnitude to overrule the entropic driving force toward a statistical mixture.

## Conclusion

The system of cages studied herein demonstrates that London dispersion forces between simple alkyl groups can dominate the behavior of a self-assembling system. This conclusion is supported by four key observations. First, **M**_4_ **L^Me^**_4_ and **M**_4_ **L^Et^**_4_ adopt different stereochemical configurations, where the Me-containing cages adopt an arrangement contrary both to the intrinsic stereochemical preference of the framework and solvophobic effects, favoring instead a diastereomer that optimizes Me–Me contacts. Second, **M**_4_ **L^Et^**_4_ exhibited greater stability than **M**_4_ **L^Me^**_4_, both in solution and in the gas phase, despite the otherwise stronger bonds to low-spin Fe^II^ in **Fe**_4_ **L^Me^**_4_. Third, both **L^Me^** and **L^Et^** incorporated abnormally high amounts of Zn^II^ over Fe^II^ into the cages, despite the intrinsic drive to bind Fe^II^ to the strong-field pyridine-imine ligands at the cage vertices. Fourthly, a system of mixed ligands and metals exhibited a preference to form homoleptic, homometallic cages, matching Fe^II^ with Me and Zn^II^ with Et from among a mixture of 25 cages, overcoming the entropic driving force to form a statistical mixture. The contra-steric nature of the stereoselectivity and stability trends observed, combined with the quantitative comparison of gas-phase stability through CID-MS, allowed the ruling out of alternative driving forces such as subtle steric strain or solvophobic effects.

The dominance of LD in governing this system thus emerges from synergy between key structural features: backbone rigidity, which fixes the geometry of alkyl–alkyl contacts and eliminates solvent competition; the high polarizability of the alkyl groups, amplified by the truxene backbone; and the inclusion of multiple instances of each alkyl–alkyl interaction.

These results establish the use of supramolecular capsules as a platform to study and quantify LD, and demonstrate that LD can overcome multiple competing driving forces to govern self-assembly within complex systems. Moving forward, cages which coencapsulate different types of alkyl groups cage can be envisaged, which would allow for more subtle tuning of the interactions between the confined groups, and thus serve as a sensitive platform to quantify alkyl–alkyl LD.

## Supplementary Material



## Notes

Experimental procedures, characterization data for new compounds, details of computational studies, and a comparison between the geometry of the cages (PDF)
