# Supplementary material for: London Dispersion Governs Stereochemistry, Stability, and Self-Sorting in a System of M4L4 Cages
Source: J Am Chem Soc. 2026 May 21;148(21):22253–62. doi: 10.1021/jacs.6c05686 (PMC13244474; doi:10.1021/jacs.6c05686)
Supplement: Supplementary file 1 [file ja6c05686_si_001.pdf]

Supporting information

London dispersion governs stereochemistry, stability, and self-sorting in a system of M_4L_4 cages

Itai Massad,[1] James T. F. Dobson,[1] Paula C. P. Teeuwen,[1] Tanya K. Ronson[1] and Jonathan R. Nitschke[1*]

[1]Yusuf Hamied Department of Chemistry, University of Cambridge, Cambridge, UK.

1. General information....2
2. Synthesis and characterization of $\mathbf{A^{Me}}$....3
3. Synthesis and characterization of $\mathbf{M_4L^R}_4$ cages....5
$\mathbf{Zn_4L^{Et}}_4$....5
$\mathbf{Fe_4L^{Me}}_4$....5
$\mathbf{Zn_4L^{Me}}_4$....13
$\mathbf{Fe_4L^{Et}}_4$....20
4. Spin-crossover studies on $\mathbf{Fe_4L^{Et}}_4$....29
5. X-ray crystallography....32
$\mathbf{Fe_4L^{Me}}_4$....32
$\mathbf{Fe_4L^{Et}}_4$....33
$\mathbf{Zn_4L^{Me}}_4$....36
6. Distance, angle and volume comparisons....38
7. Computational studies....40
8. Competition experiments....42
$\mathbf{L^{Me}}$ vs. $\mathbf{L^{Et}}$ with limiting $\mathbf{M^{II}}$....42
$\mathbf{Fe^{II}}$ vs. $\mathbf{Zn^{II}}$ with limiting $\mathbf{A^{triazine}}$....47
$\mathbf{Fe^{II}}$ vs. $\mathbf{Zn^{II}}$ with limiting $\mathbf{A^R}$....50
9. CID-MS experiments....55
10. Self-sorting experiments....57
11. References....62

1. General information

Unless stated otherwise, reactants and solvents were purchased from commercial suppliers and used without further purification.

NMR spectra were recorded on the following instruments: Bruker 400 MHz Avance III HD Smart Probe, Bruker 500 MHz Avance III HD Smart Probe, Bruker 500 MHz Avance III DCH, Bruker 700 MHz AVIII Cryo. Chemical shifts (δ) are given in parts per million (ppm) from low to high field and referenced using the residual solvent signal. ($CDCl_3$: ^{1}H = 7.26 ppm, ^{13}C = 77.026 (centre of the deuterated solvent triplet); CD_3CN: ^{1}H = 1.94 ppm, ^{13}C = 118.26 ppm). Coupling constants (*J*) are given in Hertz (Hz). Signal multiplicity is denoted by the following abbreviations: singlet (s), doublet (d), broad (br). Spectra were measured at 298 K, unless otherwise stated.

High-resolution electrospray ionization mass spectrometry (HR-ESI-MS) were recorded on a Waters Synapt G2-Si spectrometer, with samples directly infused using a syringe pump (420 µl/h).

X-ray diffraction data were collected at Beamline I19 of Diamond Light Source (see section 5).

2. Synthesis and characterization of A^{Me}

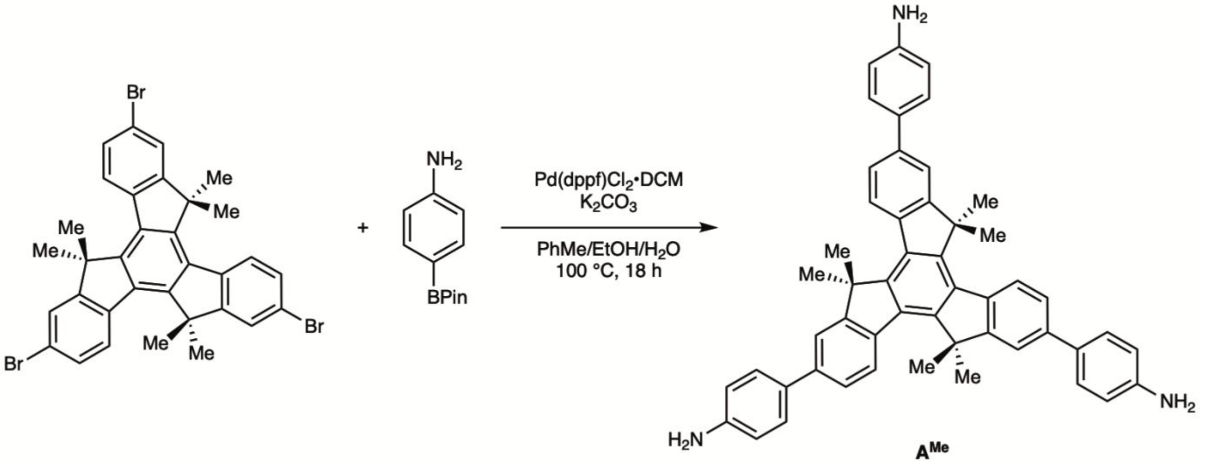

Figure S1. Synthesis of $\mathbf{A^{Me}}$.

In a 100 ml three-neck flask equipped with a reflux condenser, a mixture of toluene, ethanol and deionized water (15/9/6 ml) was sparged with N_2 for 30 minutes. 2,7,12-tribromo-5,5,10,10,15,15-hexamethyltruxene[1] (1 g, 1.43 mmol), 4-aminophenylboronic acid pinacol ester (3.15 equiv., 1 g, 3.5 mmol), potassium carbonate (4.75 equiv., 938 mg, 6.8 mmol) and Pd(dppf)Cl_2•DCM (6.3 mol%, 74 mg, 90 µmol) were then added, and the mixture was stirred at 100 °C overnight under an N_2 atmosphere. Upon complete consumption of the tribromide starting material (as observed by TLC), the mixture was allowed to cool to room temperature and was then diluted with water and extracted thrice with dichloromethane (50 ml). The solvent was removed *in vacuo*, and the crude product was purified by column chromatography on silica gel, eluting with a gradient of 0-0.5-1-2% methanol in dichloromethane. $\mathbf{A^{Me}}$ was obtained as an off-white solid (883 mg, 1.26 mmol, 88% yield).

^{1}H-NMR (400 MHz, $CDCl_3$) δ 8.32 (d, *J* = 8.3 Hz, 1H), 7.70 (d, *J* = 1.8 Hz, 1H), 7.65 – 7.58 (m, 1H), 7.57 (d, *J* = 6.5 Hz, 2H), 6.83 (d, *J* = 8.5 Hz, 2H), 3.78 (bs, 2H), 1.95 (s, 6H).

^{13}C-NMR (176 MHz, $CDCl_3$) δ 158.2, 148.0, 145.9, 139.6, 135.5, 135.1, 131.7, 128.1, 125.8, 124.6, 120.3, 115.5, 46.9, 24.2.

ESI-HRMS $C_{51}H_{46}N_3$ $[M+H]^+$: calculated m/z 700.3692, found m/z 700.3715.

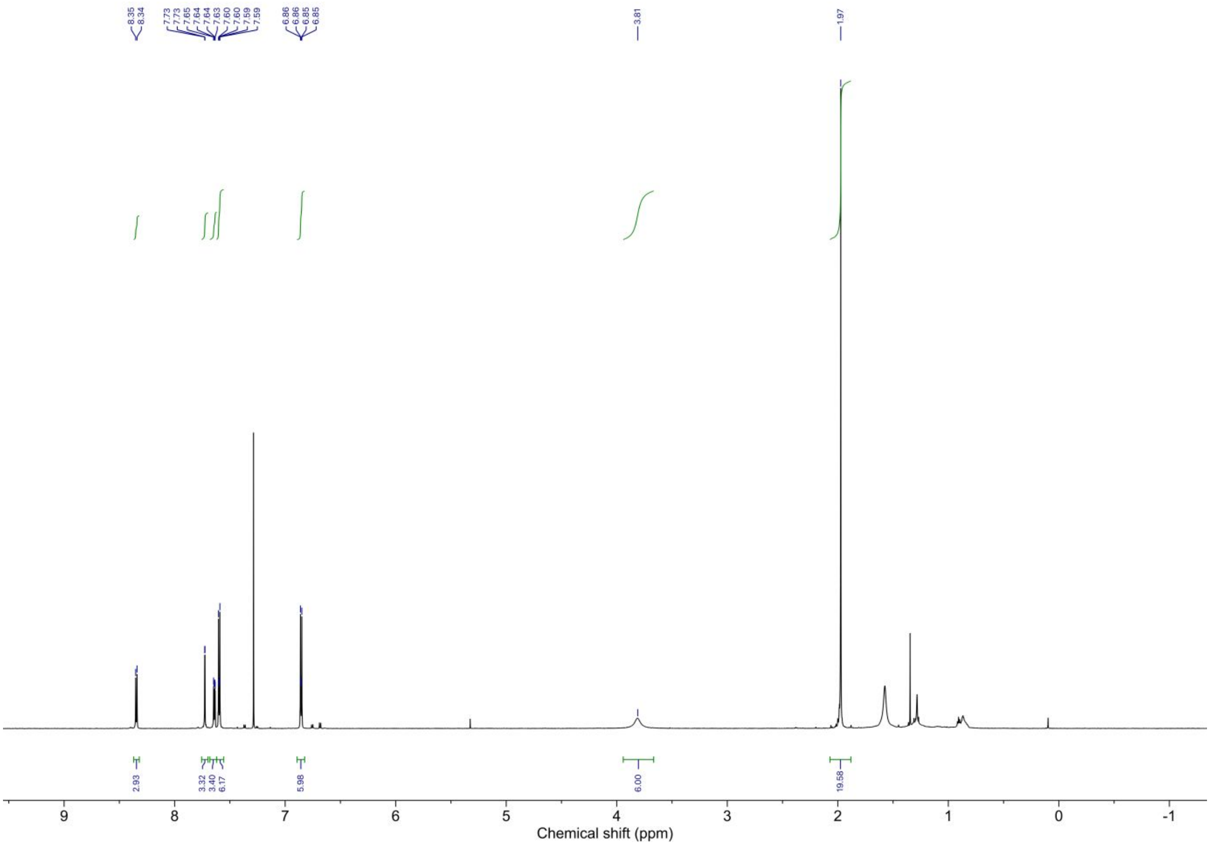

Figure S2. ^{1}H-NMR spectrum of **A^{Me}** (400 MHz, $CDCl_3$).

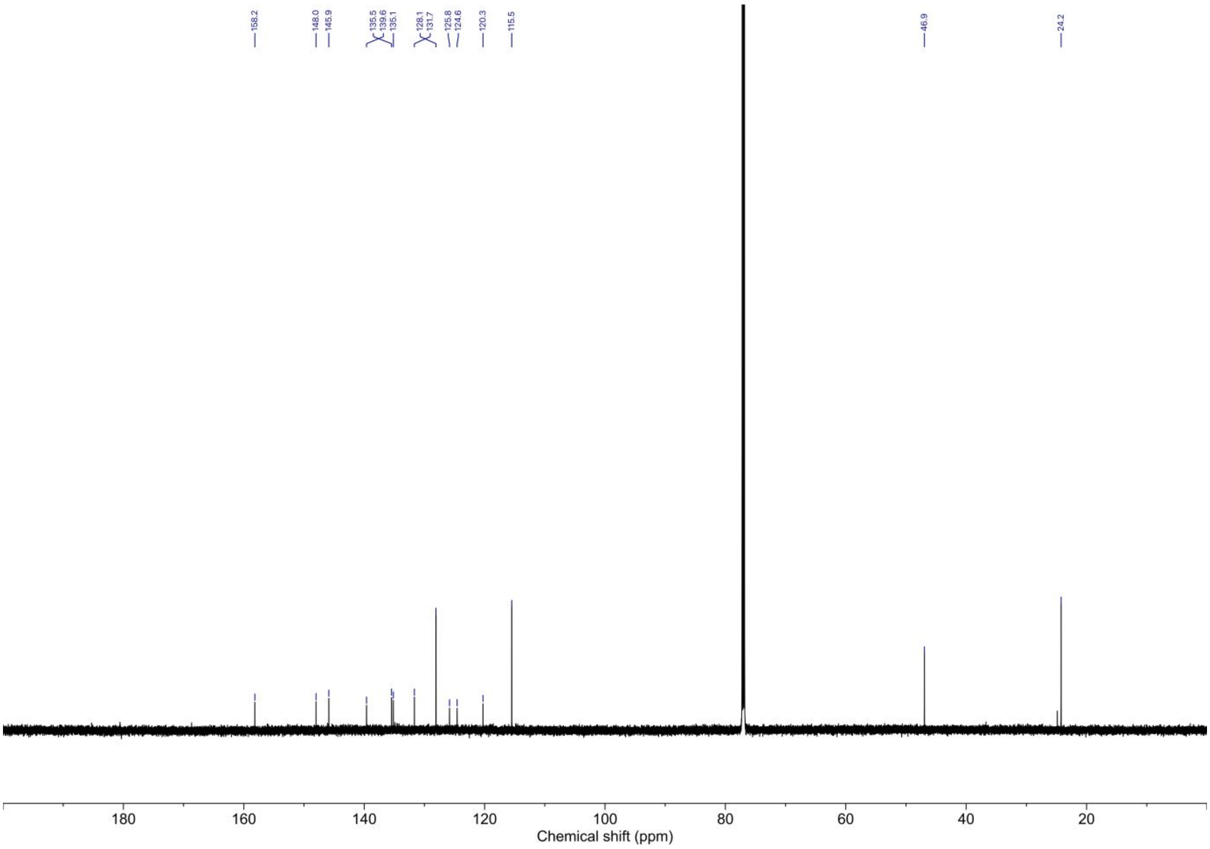

Figure S3. ^{13}C-NMR spectrum of **A^{Me}** (176 MHz, $CDCl_3$).

3. Synthesis and characterization of $M_4L^R_4$ cages

$Zn_4L^{Et}_4$

Prepared according to a reported procedure.[2]

$Fe_4L^{Me}_4$

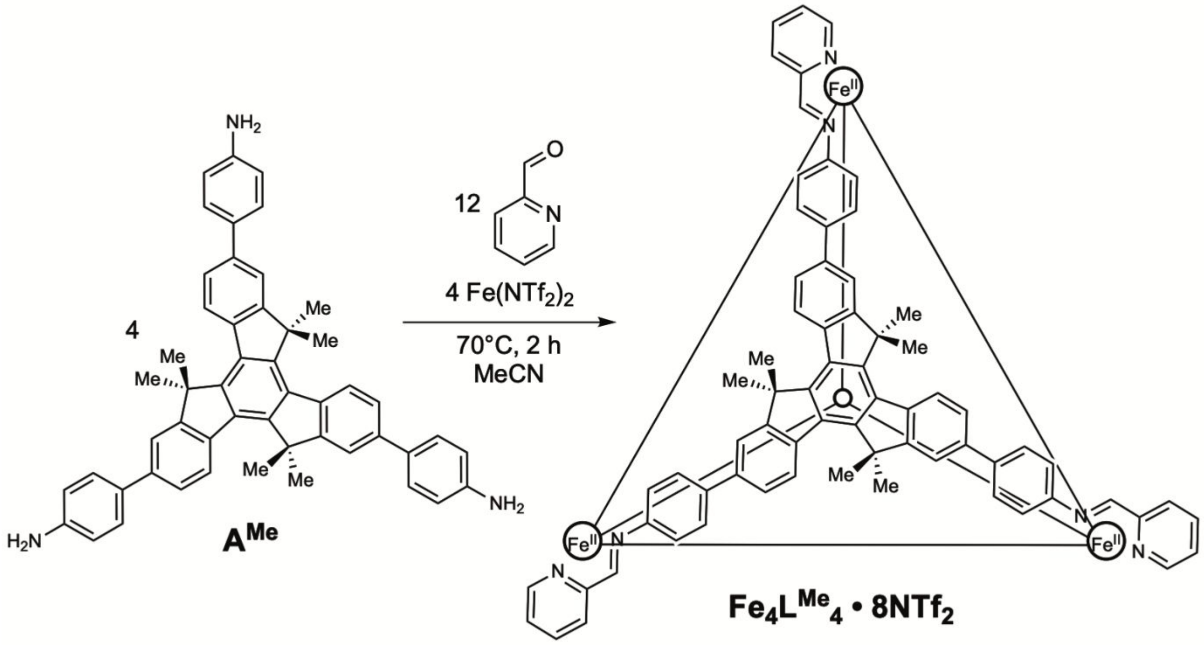

Figure S4. Synthesis of **$Fe_4L^{Me}_4$**.

To a vial, **A^{Me}** (20 mg, 28.6 µmol), $Fe(NTf_2)_2$ (92% purity, 1 equiv., 19.2 mg, 28.6 µmol) and 2-formylpyridine (3 equiv., 8.2 µl, 9.2 mg, 85.7 µmol) were added. Acetonitrile (2 ml) was added, and the mixture was stirred until clear. The resulting solution was sparged with N_2 for 15 minutes and was then heated to 70 °C for 2 hours. The reaction mixture was allowed to cool to room temperature and was then added to diethyl ether (20 ml). The precipitate was isolated by centrifugation and washed with two more portions of diethyl ether. The resulting solid was dried *in vacuo* to afford **$Fe_4L^{Me}_4$** as a black crystalline solid (43 mg, 6.8 µmol, 95% yield).

^{1}H-NMR (500 MHz, CD_3CN) δ 10.29 (s, 1H), 9.03 (d, *J* = 7.7 Hz, 1H), 8.41 (t, *J* = 7.7 Hz, 2H), 8.29 (d, *J* = 8.2 Hz, 2H), 8.18 (t, *J* = 6.4 Hz, 1H), 7.55 (d, *J* = 1.7 Hz, 1H), 7.51 (dd, *J* = 8.2, 1.7 Hz, 1H), 7.34 (d, *J* = 7.7 Hz, 2H), 5.76 (d, *J* = 8.0 Hz, 2H), 1.81 (s, 3H), 1.55 (s, 3H).

^{13}C-NMR (126 MHz, CD_3CN) δ 171.8, 159.0, 157.2, 156.8, 153.3, 149.5, 144.3, 140.8, 139.9, 136.6, 136.1, 134.3, 129.3, 127.2, 126.9, 124.6, 124.3, 122.3, 122.1, 119.5, 117.0, 47.6, 25.1, 22.4.

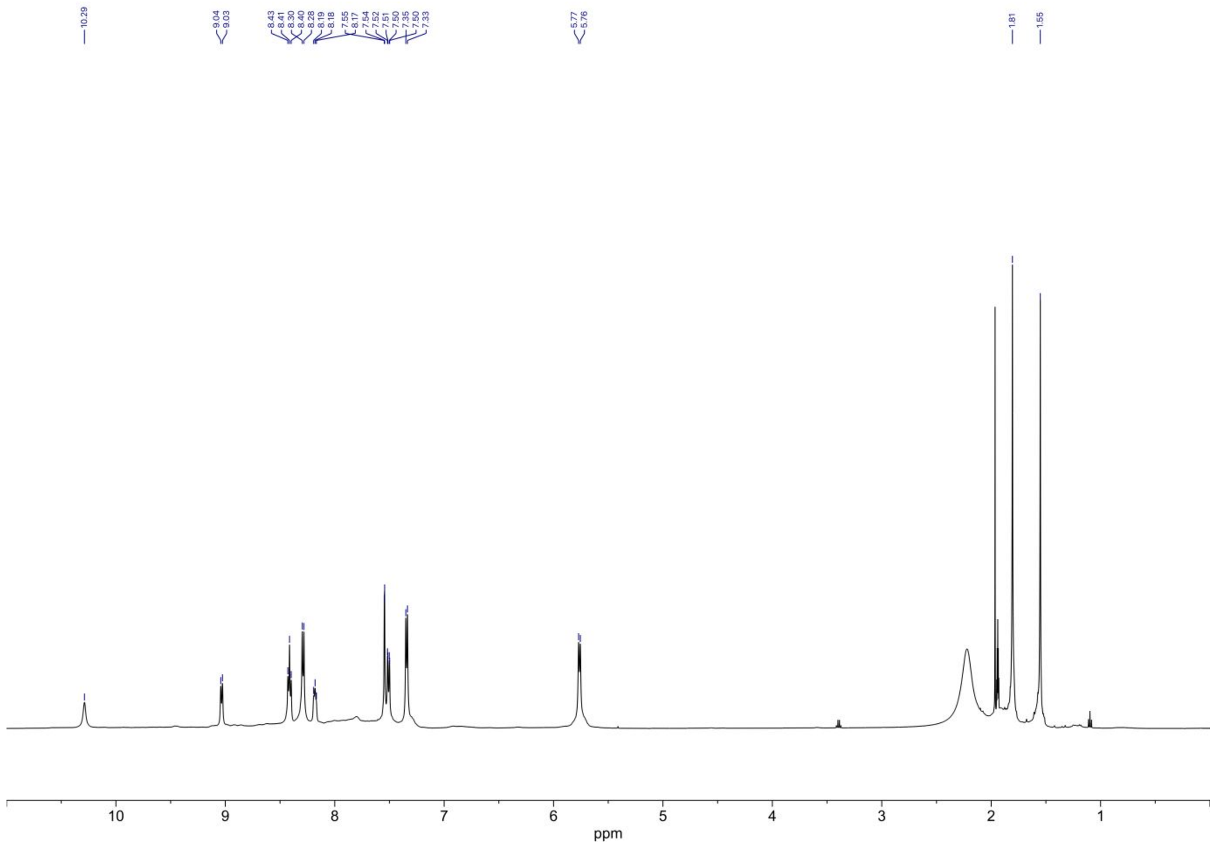

Figure S5. ^{1}H-NMR spectrum of **Fe$_4$L^{Me}$_4$** (500 MHz, CD_3CN).

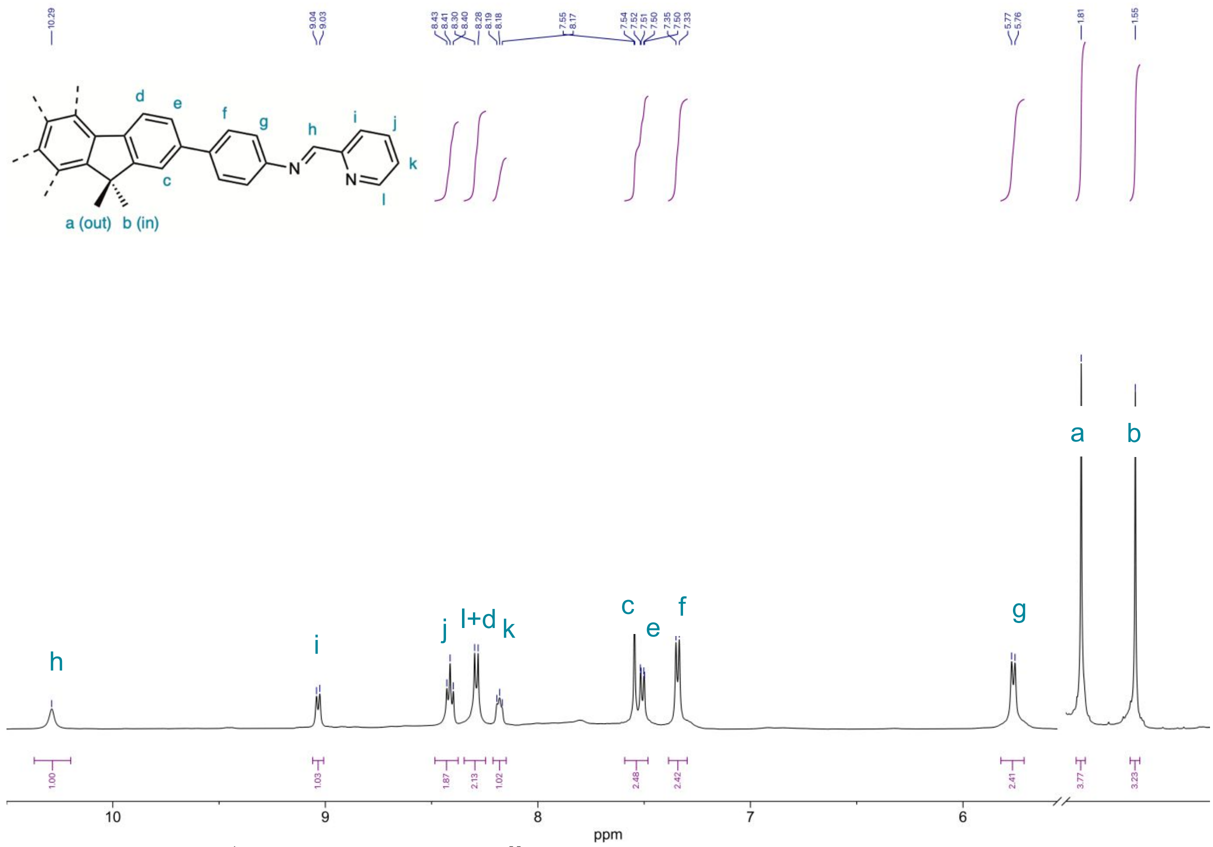

Figure S6. Assigned ^{1}H-NMR spectrum of **Fe$_4$L^{Me}$_4$** (500 MHz, CD_3CN).

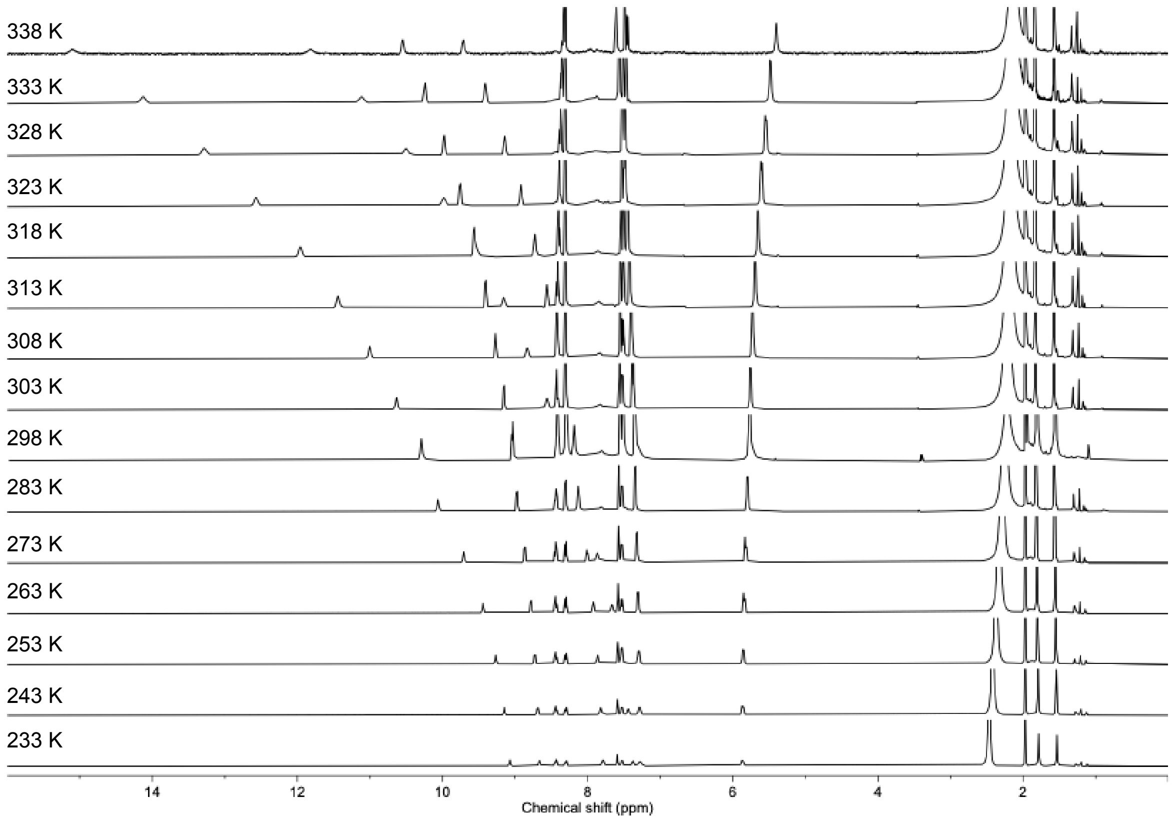

Figure S7. ^{1}H-NMR spectra of $\mathbf{Fe_4L^{Me}_4}$ (500 MHz, CD_3CN) at temperatures from 233 to 338 K.

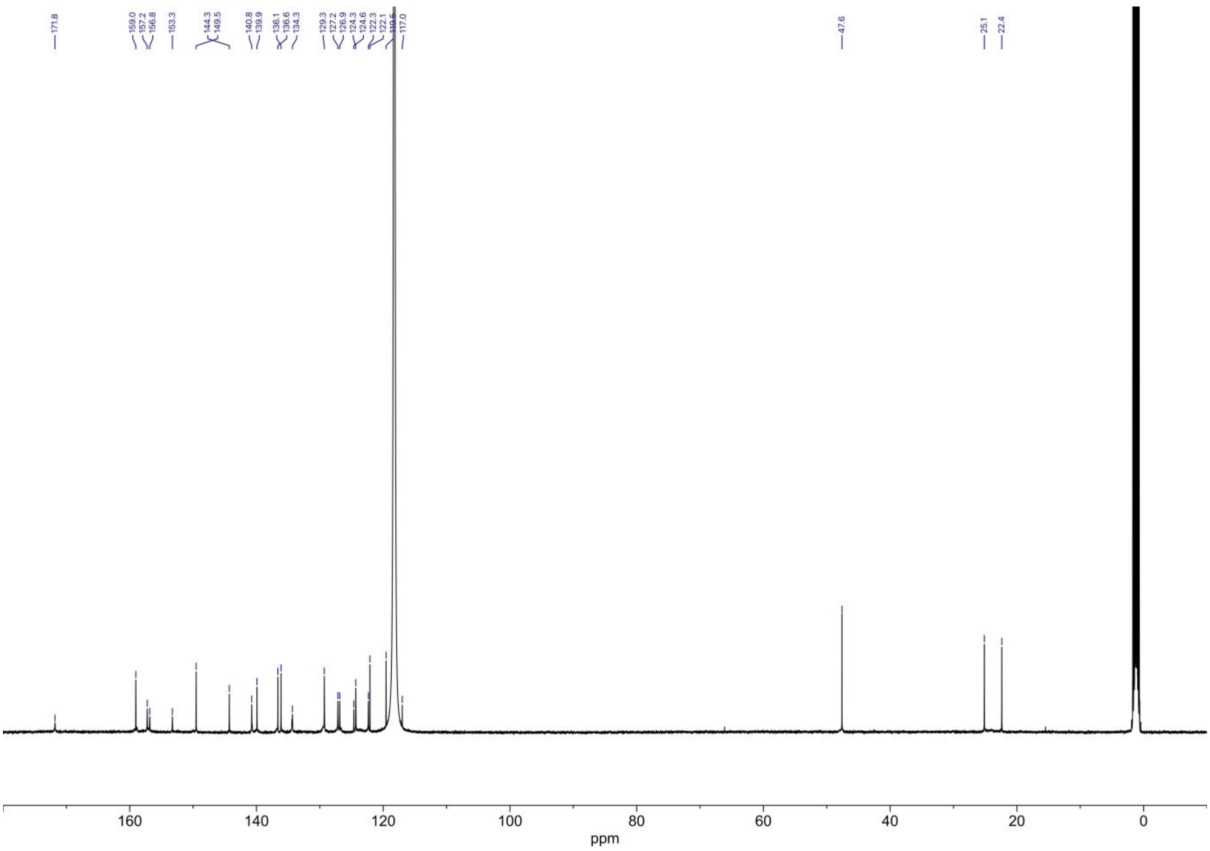

Figure S8. ^{13}C-NMR spectrum of $\mathbf{Fe_4L^{Me}}_4$ (126 MHz, CD_3CN).

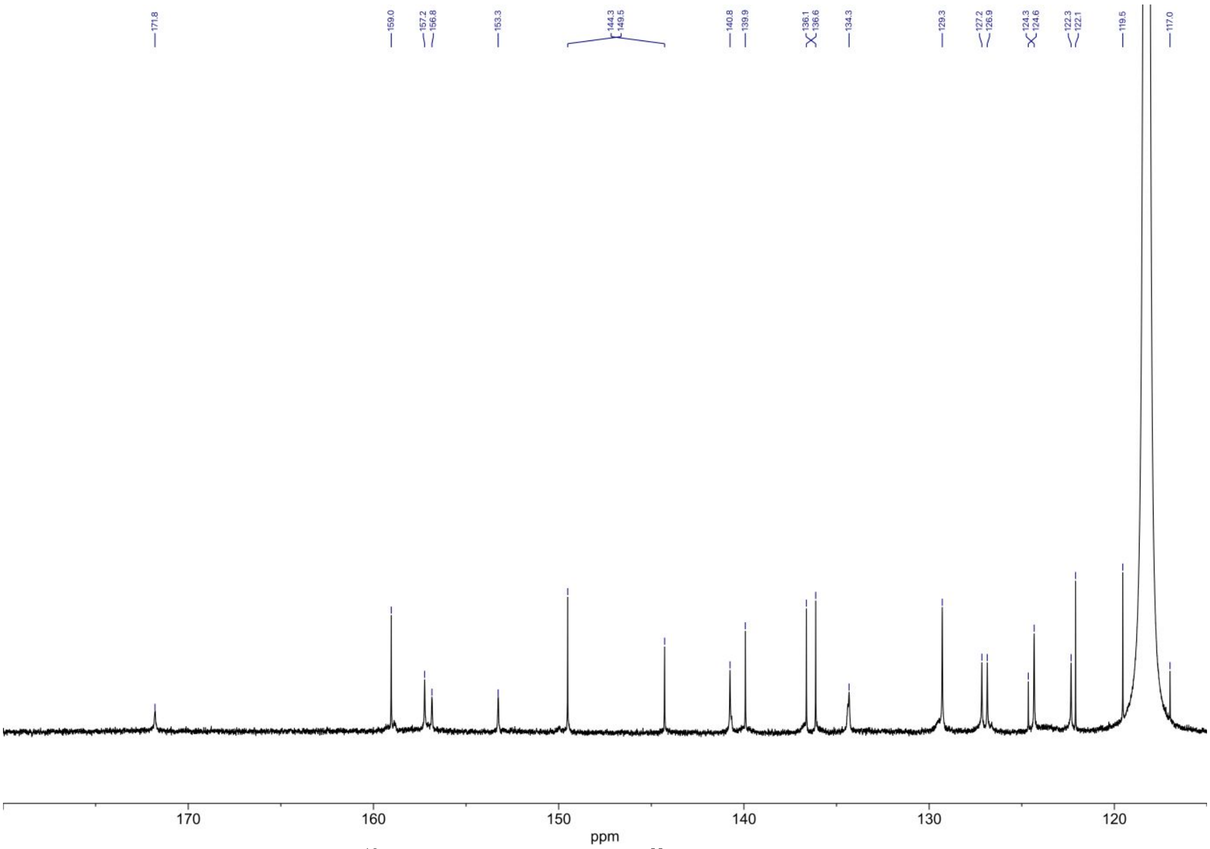

Figure S9. Aromatic region of the ^{13}C-NMR spectrum of $\mathbf{Fe_4L^{Me}}_4$ (126 MHz, CD_3CN).

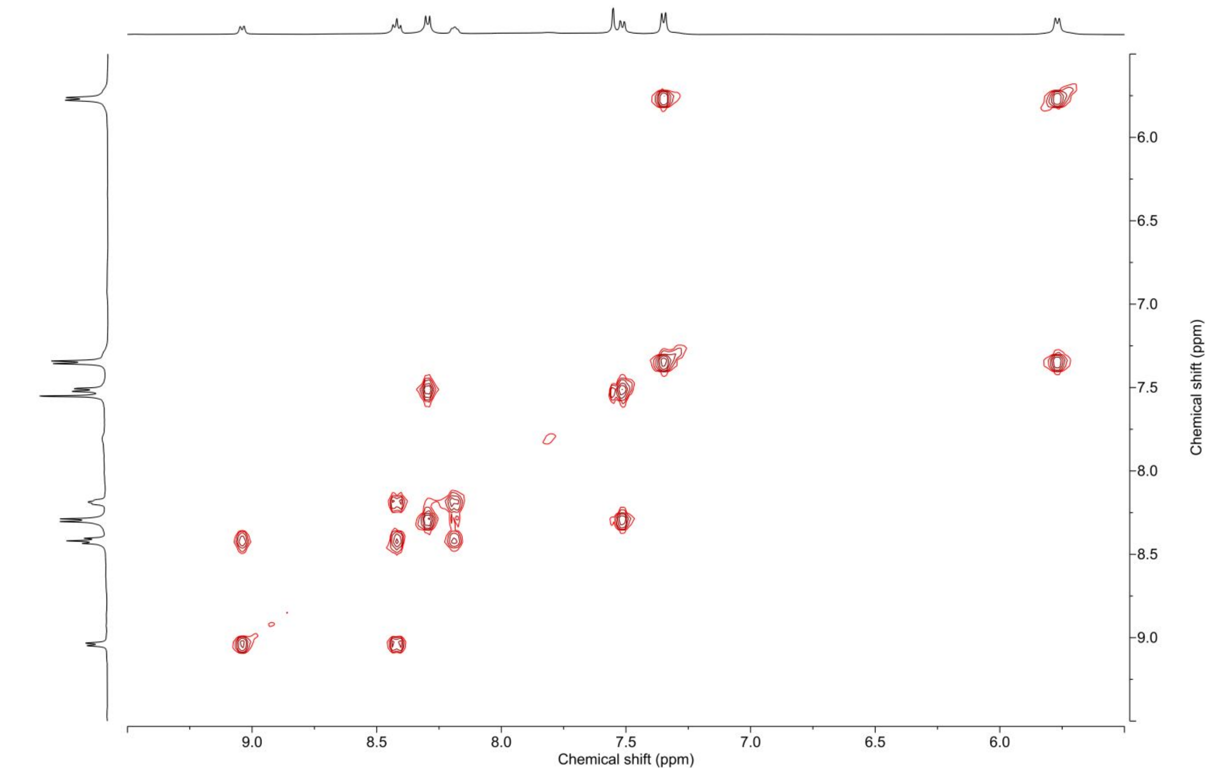

Figure S10. ^{1}H-^{1}H DQF-COSY spectrum of **Fe**$_4$**L**$^{\mathbf{Me}}_4$ (500 MHz, CD_3CN).

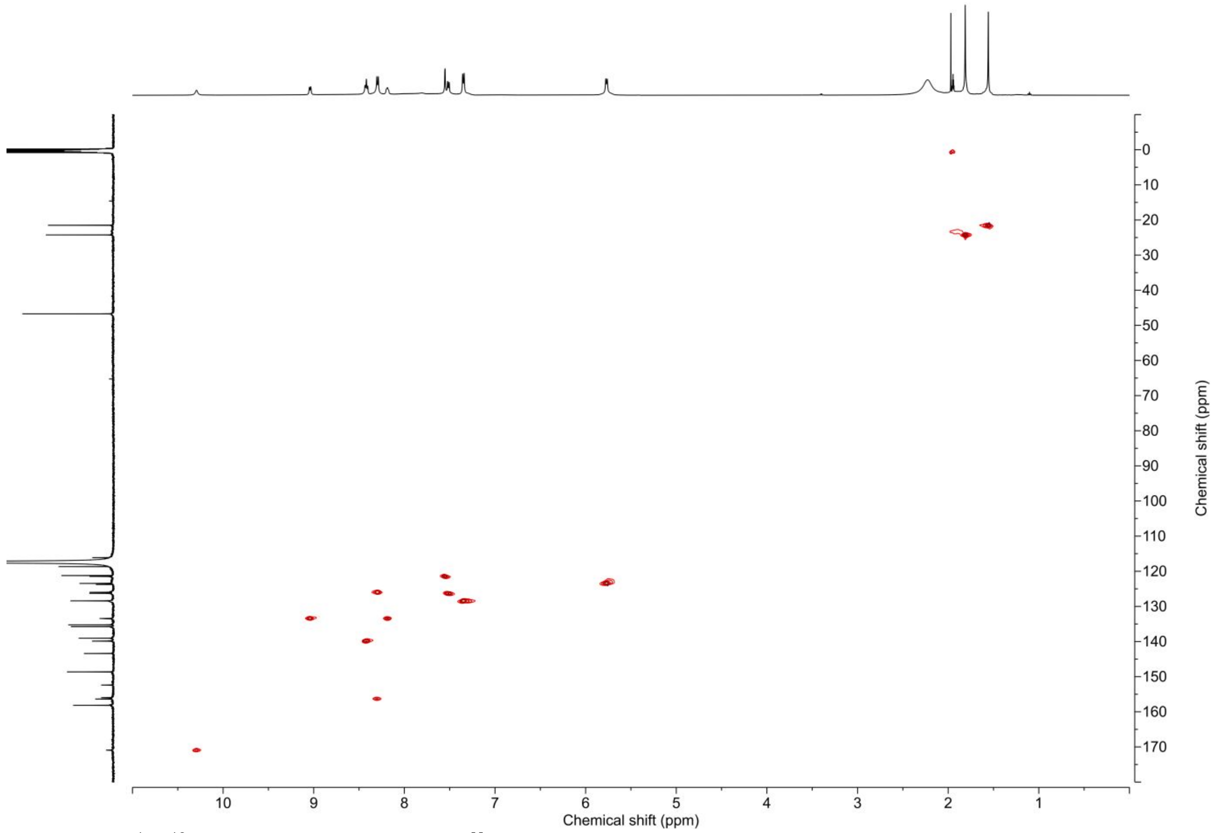

Figure S11. ^{1}H-^{13}C HSQC spectrum of **Fe**$_4$**L**$^{\mathbf{Me}}_4$ (500 MHz, 126 MHz, CD_3CN).

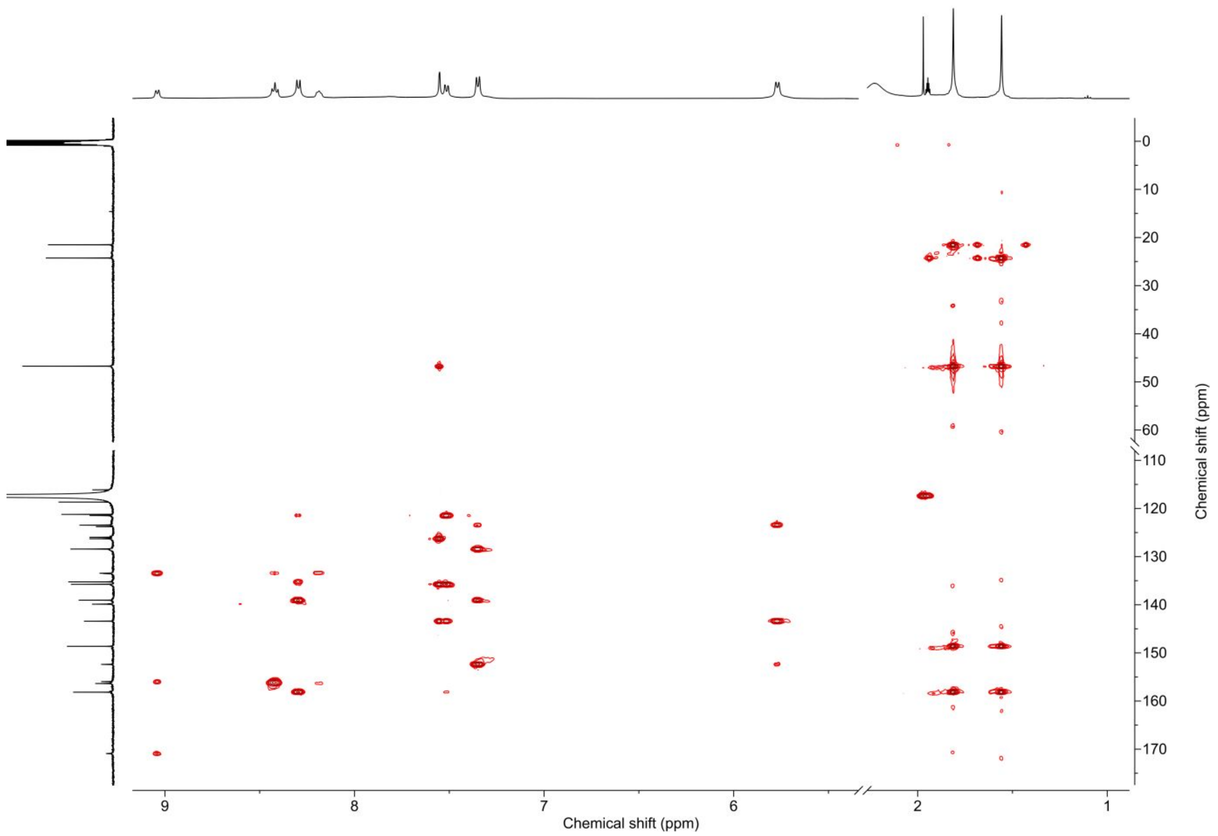

Figure S12. ^{1}H-^{13}C HMBC spectrum of **Fe$_4$L$^{\mathbf{Me}}$$_4$** (500 MHz, 126 MHz, CD_3CN).

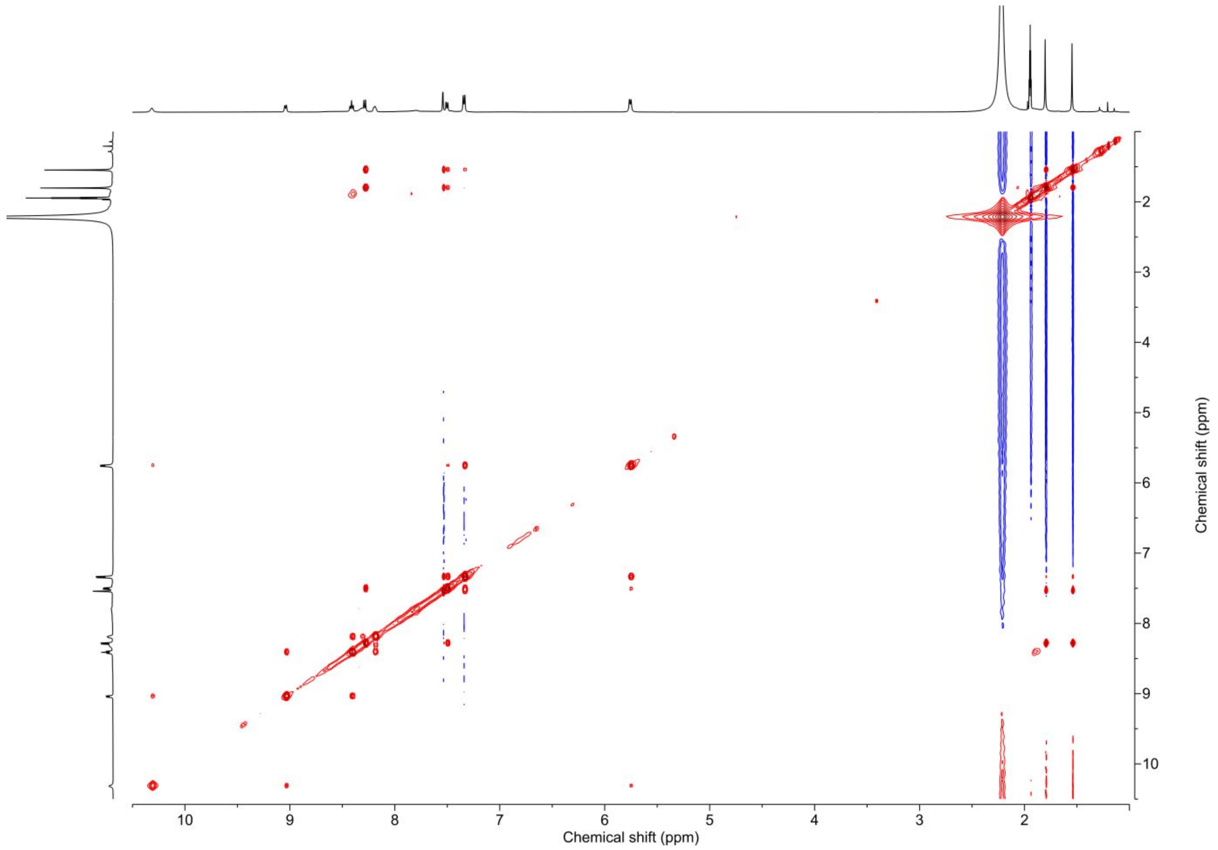

Figure S13. ^{1}H-^{1}H NOESY spectrum of **Fe$_4$L$^{\mathbf{Me}}$$_4$** (500 MHz, CD_3CN).

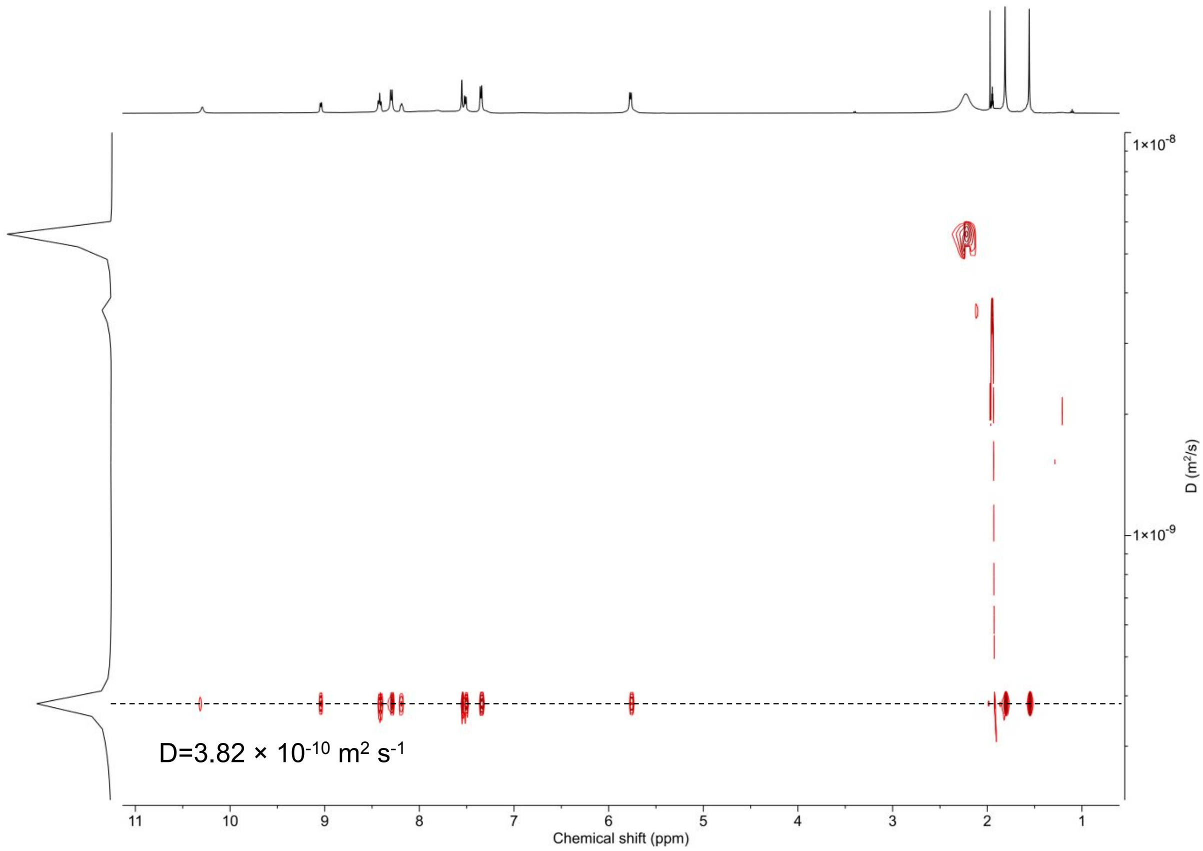

Figure S14. ^{1}H-DOSY of **Fe$_4$L^{Me}$_4$** (500 MHz, CD_3CN).

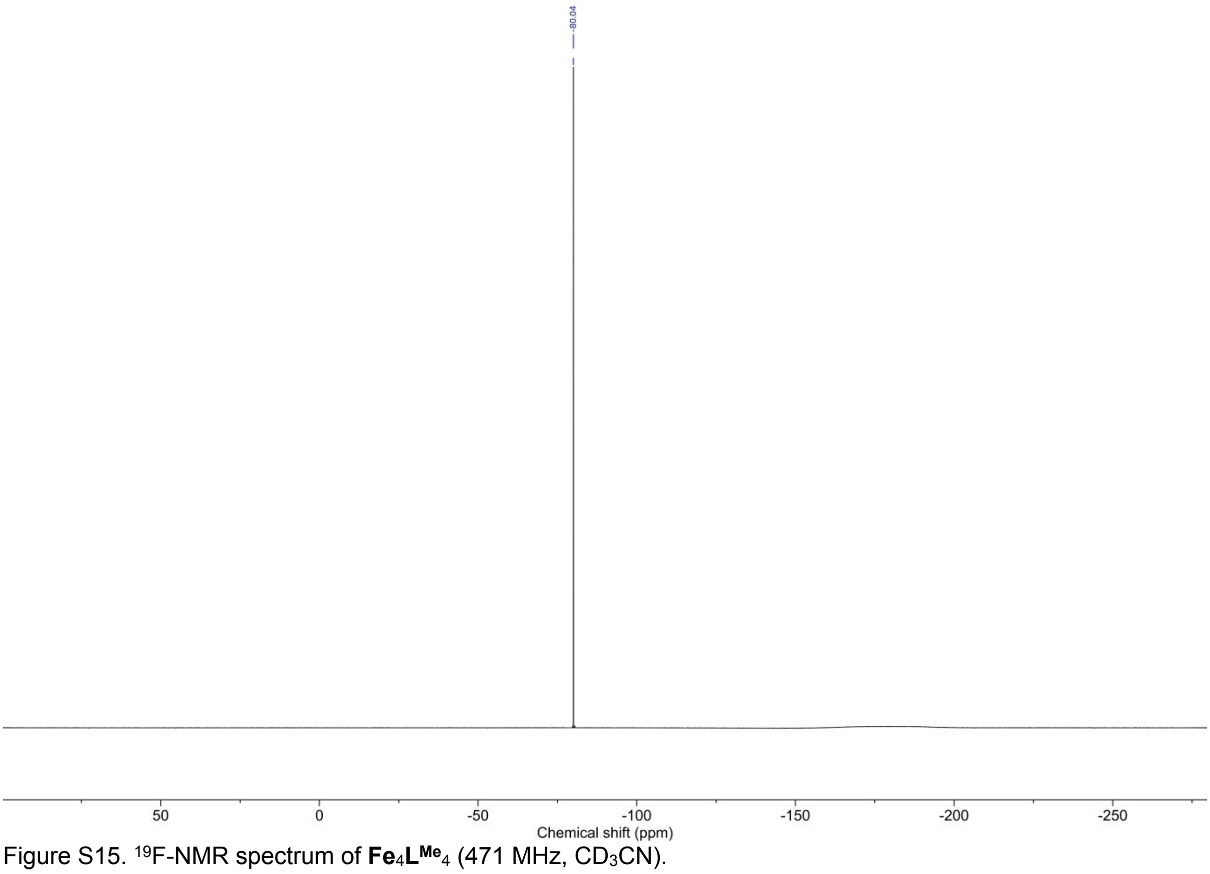

Figure S15. ^{19}F-NMR spectrum of **Fe$_4$L^{Me}$_4$** (471 MHz, CD_3CN).

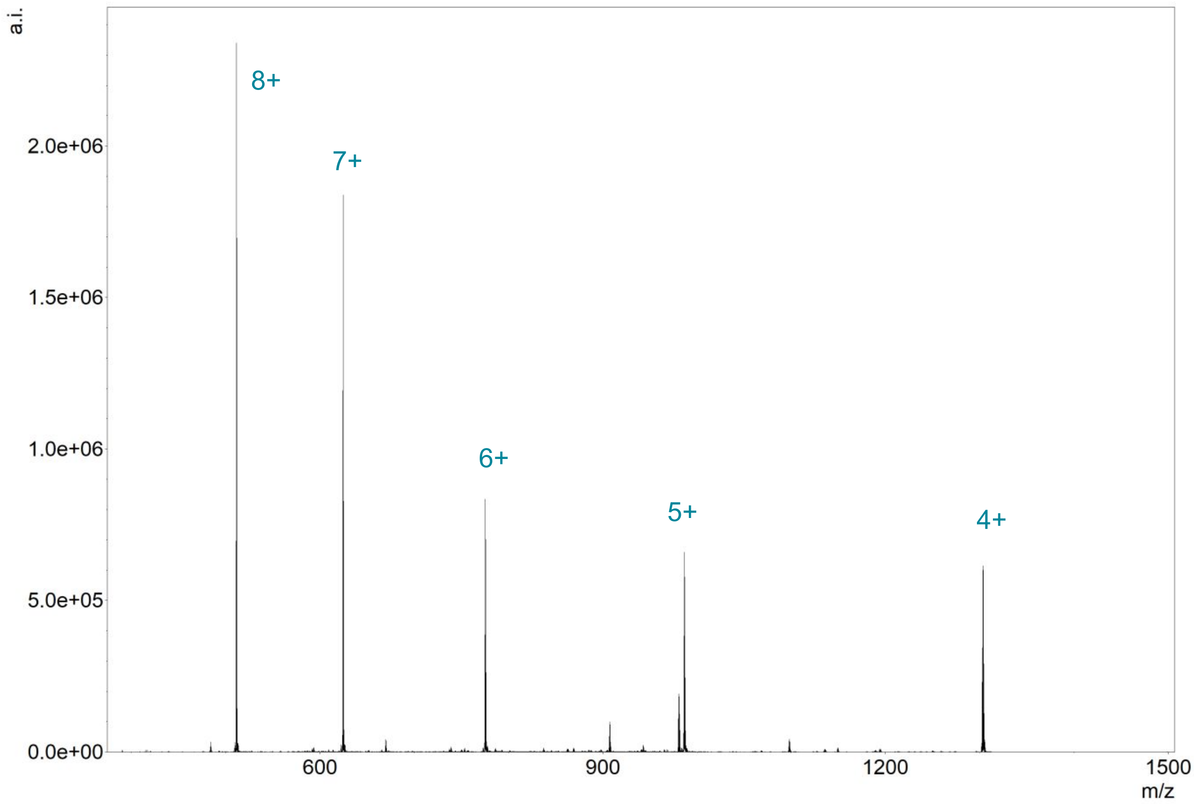

Figure S16. High-resolution ESI-mass spectrum of **$Fe_4L^{Me}{}_4$**.

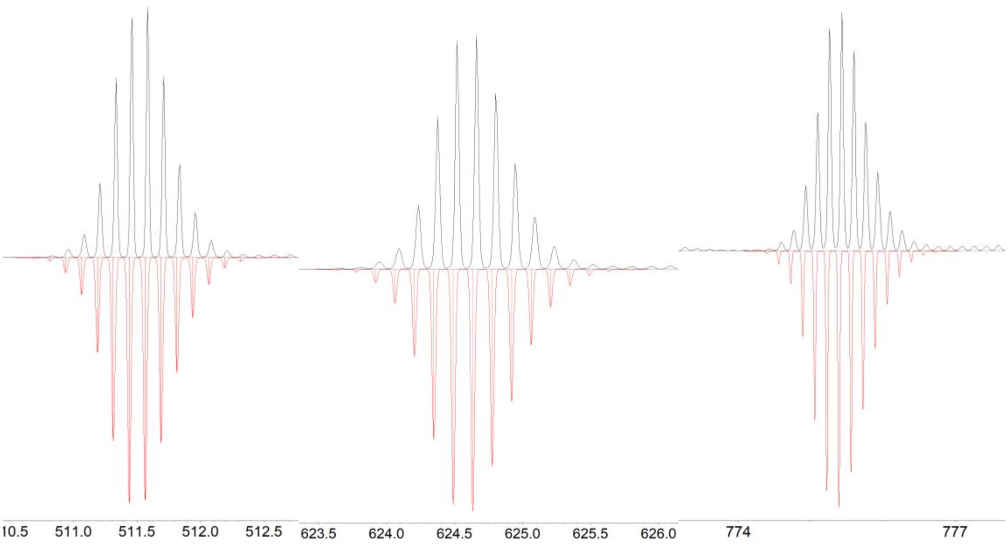

Figure S17. From left to right, peaks corresponding to the 8+, 7+, and 6+ ions of **$Fe_4L^{Me}{}_4$** (black), overlayed with the calculated m/z (red).

$Zn_4L^{Me}_4$

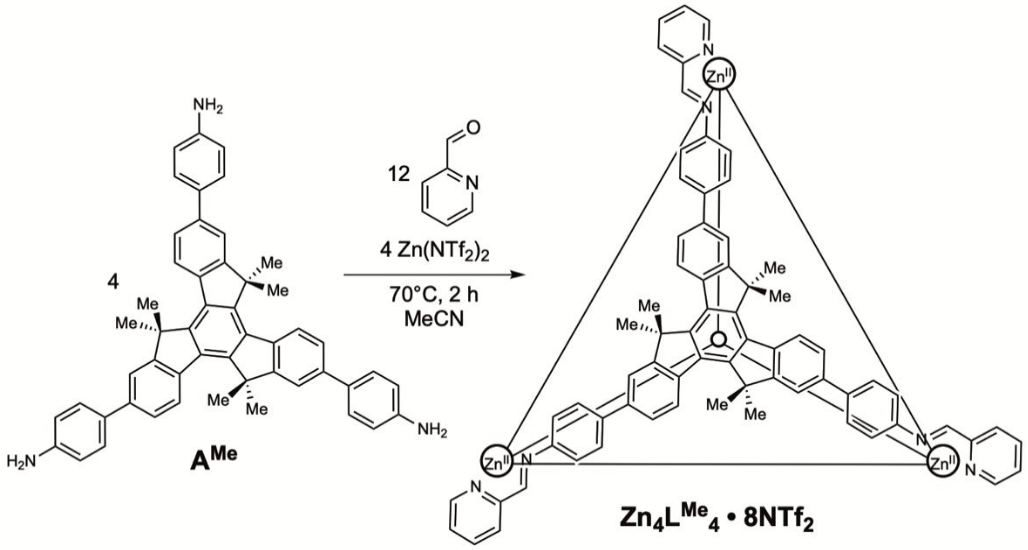

Figure S18. Synthesis of **$Zn_4L^{Me}_4$**.

To a vial, **A^{Me}** (20 mg, 28.6 μmol), $Zn(NTf_2)_2$ (93% purity, 1 equiv., 19.2 mg, 28.6 μmol) and 2-formylpyridine (3 equiv., 8.2 μl, 9.2 mg, 85.7 μmol) were added. Acetonitrile (2 ml) was added, and the mixture was stirred until clear. The resulting solution was then heated to 70 °C for 2 hours. The reaction mixture was allowed to cool to room temperature and was then added to diethyl ether (20 ml). The precipitate was isolated by centrifugation and washed with two more portions of diethyl ether. The resulting solid was dried *in vacuo* to afford **$Zn_4L^{Me}_4$** as an orange crystalline solid (44 mg, 6.9 μmol, 97% yield).

^{1}H-NMR (700 MHz, CD_3CN) δ 8.87 (s, 1H), 8.52 (td, J = 7.4, 2.4 Hz, 1H), 8.40 (d, J = 7.7 Hz, 1H), 8.29 (d, J = 8.3 Hz, 1H), 7.93 (s, 2H), 7.68 (d, J = 1.8 Hz, 1H), 7.57 – 7.54 (m, 1H), 7.51 (d, J = 8.3 Hz, 2H), 6.62 (d, J = 8.3 Hz, 2H), 1.82 (s, 3H), 1.57 (d, J = 3.7 Hz, 3H).

Note: the spectrum includes additional peaks attributed to low-symmetry isomers present in minor amounts.

^{13}C-NMR (126 MHz, CD_3CN) δ 165.1, 159.2, 150.4, 149.6, 147.4, 147.2, 143.6, 142.4, 139.5, 136.8, 136.1, 132.1, 131.6, 129.3, 129.0, 127.1, 126.6, 124.7, 124.0, 123.2, 122.2, 121.8, 119.6, 117.1, 47.7, 24.6, 23.4.

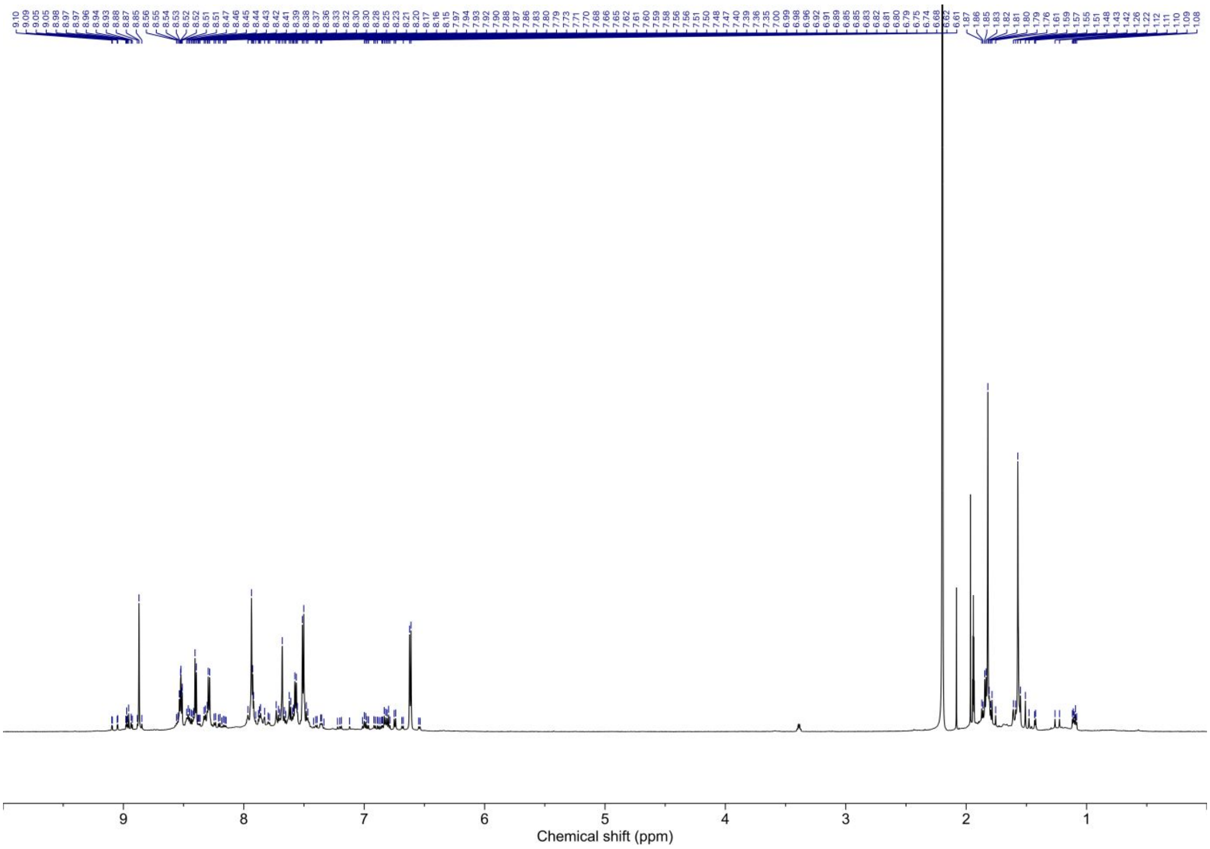

Figure S19. ^{1}H-NMR spectrum of $\mathbf{Zn_4L^{Me}}_4$ (700 MHz, CD_3CN).

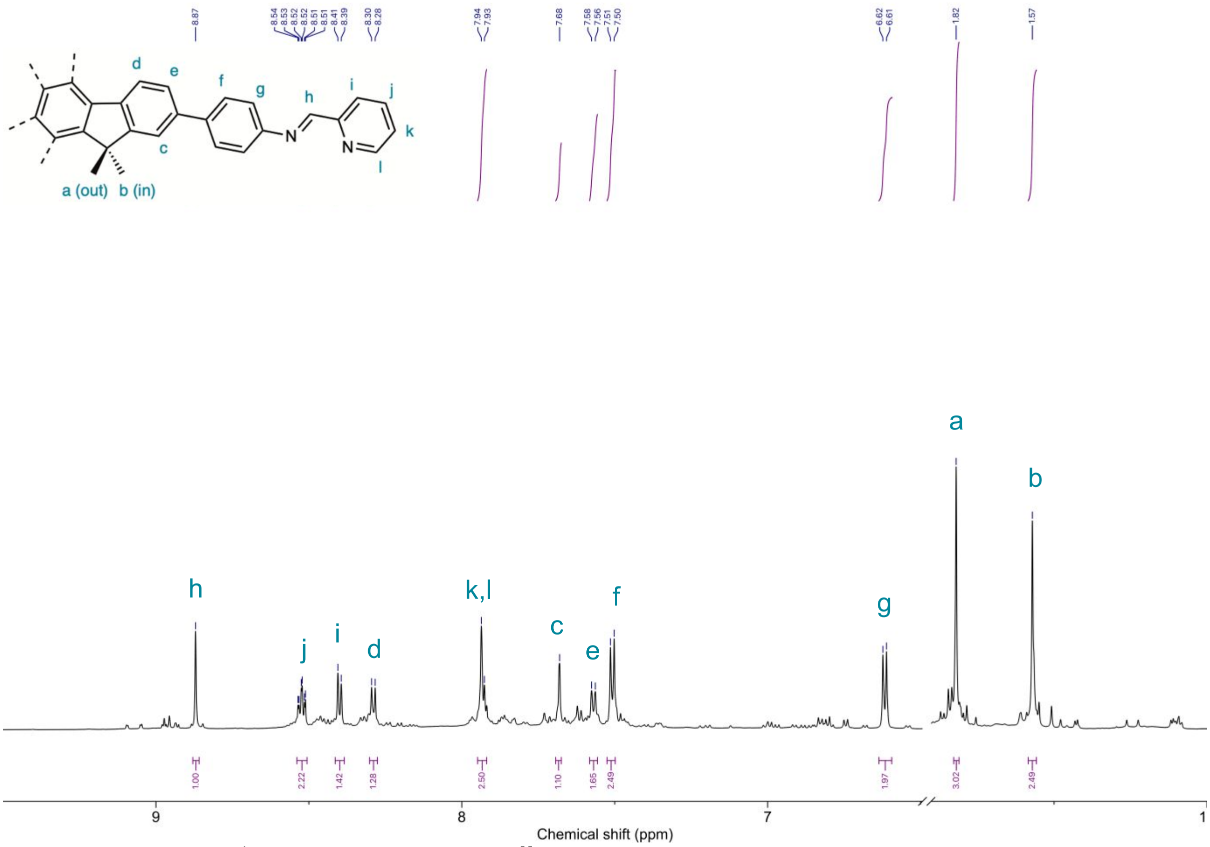

Figure S20. Assigned ^{1}H-NMR spectrum of $\mathbf{Zn_4L^{Me}}_4$ (700 MHz, CD_3CN).

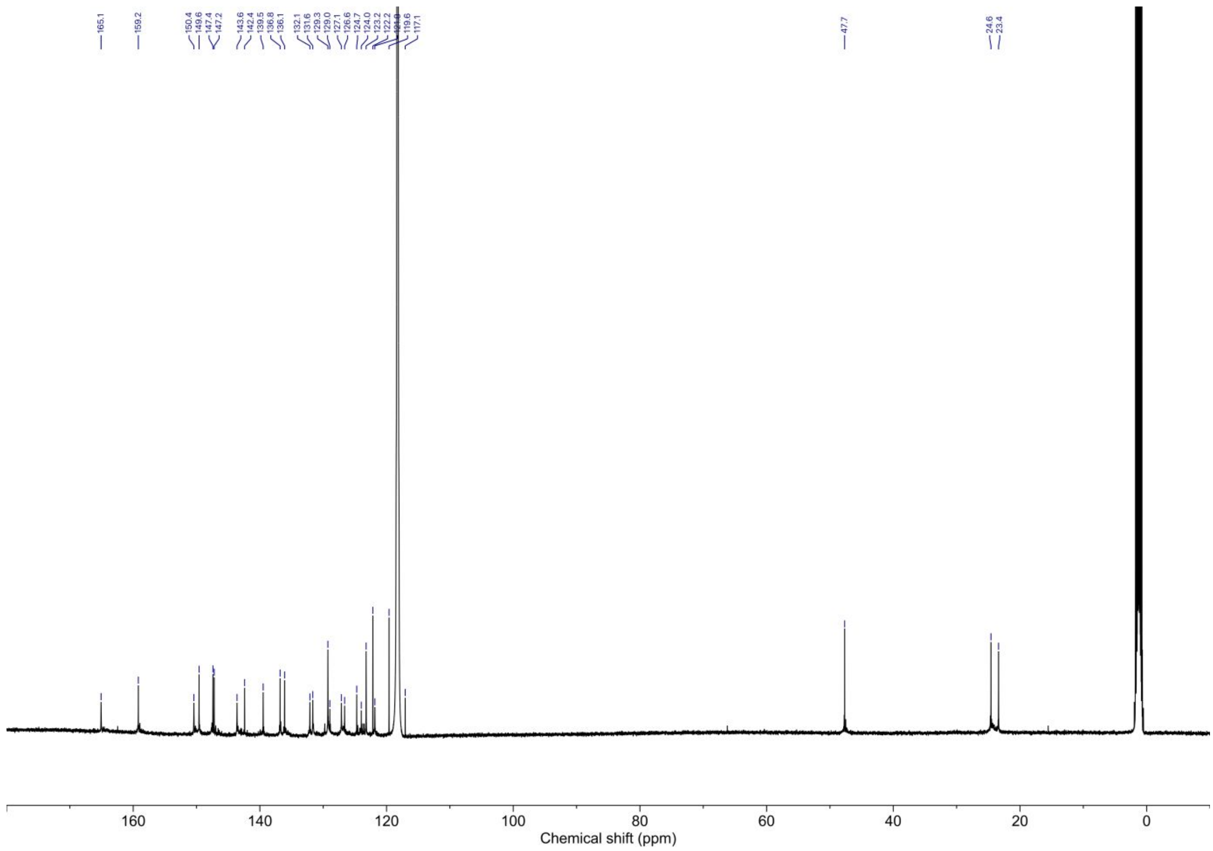

Figure S21. ^{13}C-NMR spectrum of $\mathbf{Zn_4L^{Me}_4}$ (126 MHz, CD_3CN).

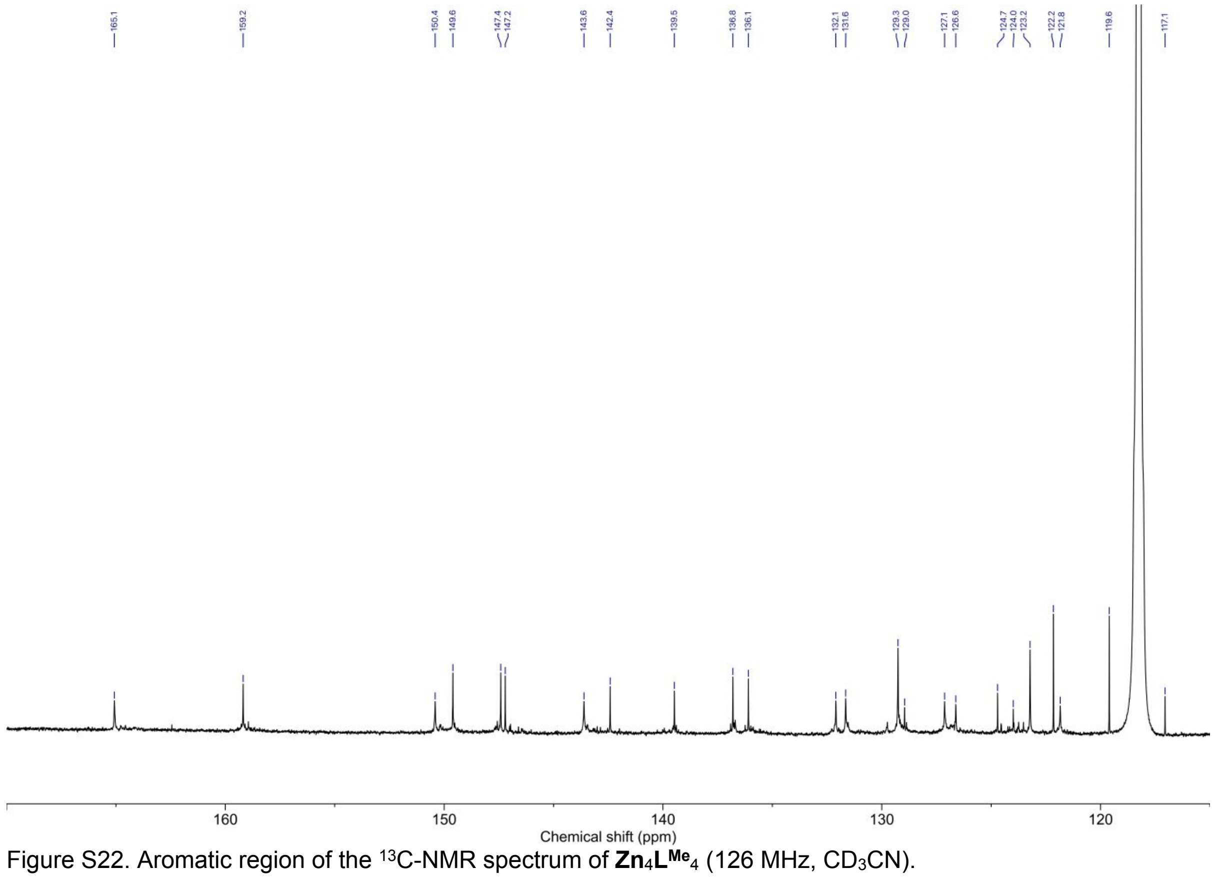

Figure S22. Aromatic region of the ^{13}C-NMR spectrum of $\mathbf{Zn_4L^{Me}_4}$ (126 MHz, CD_3CN).

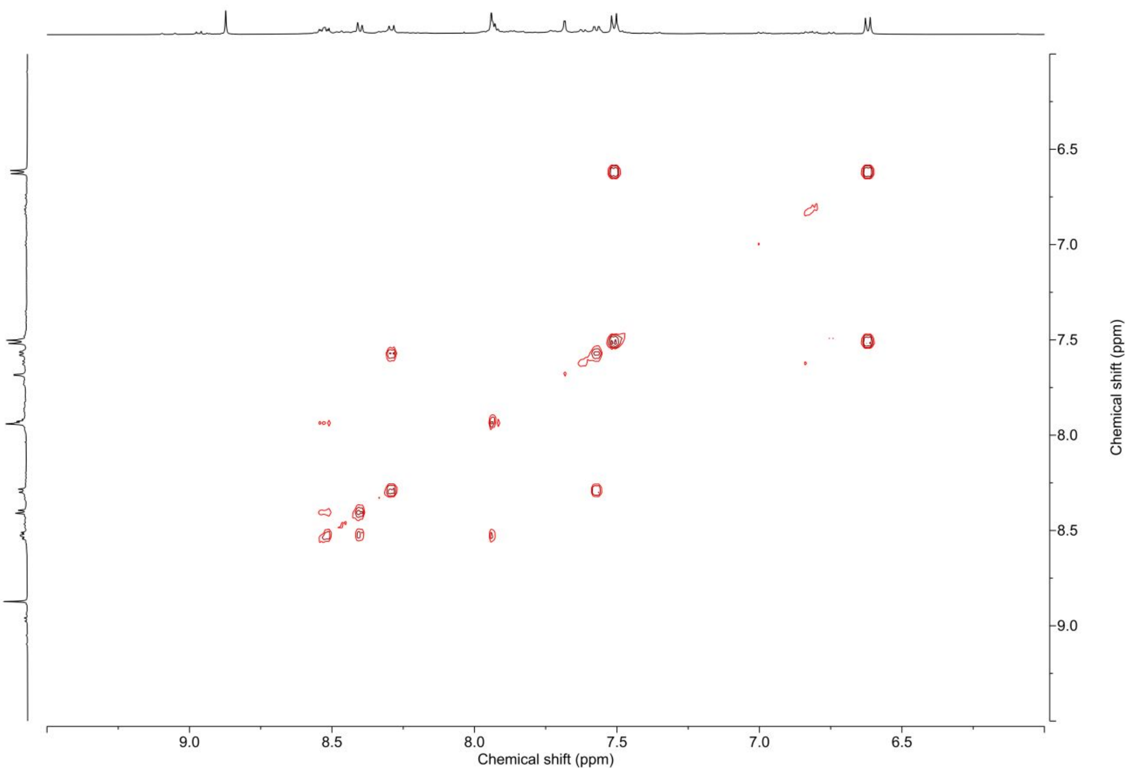

Figure S23. ^{1}H DQF-COSY spectrum of **Zn$_4$L^{Me}**$_4$ (500 MHz, CD_3CN).

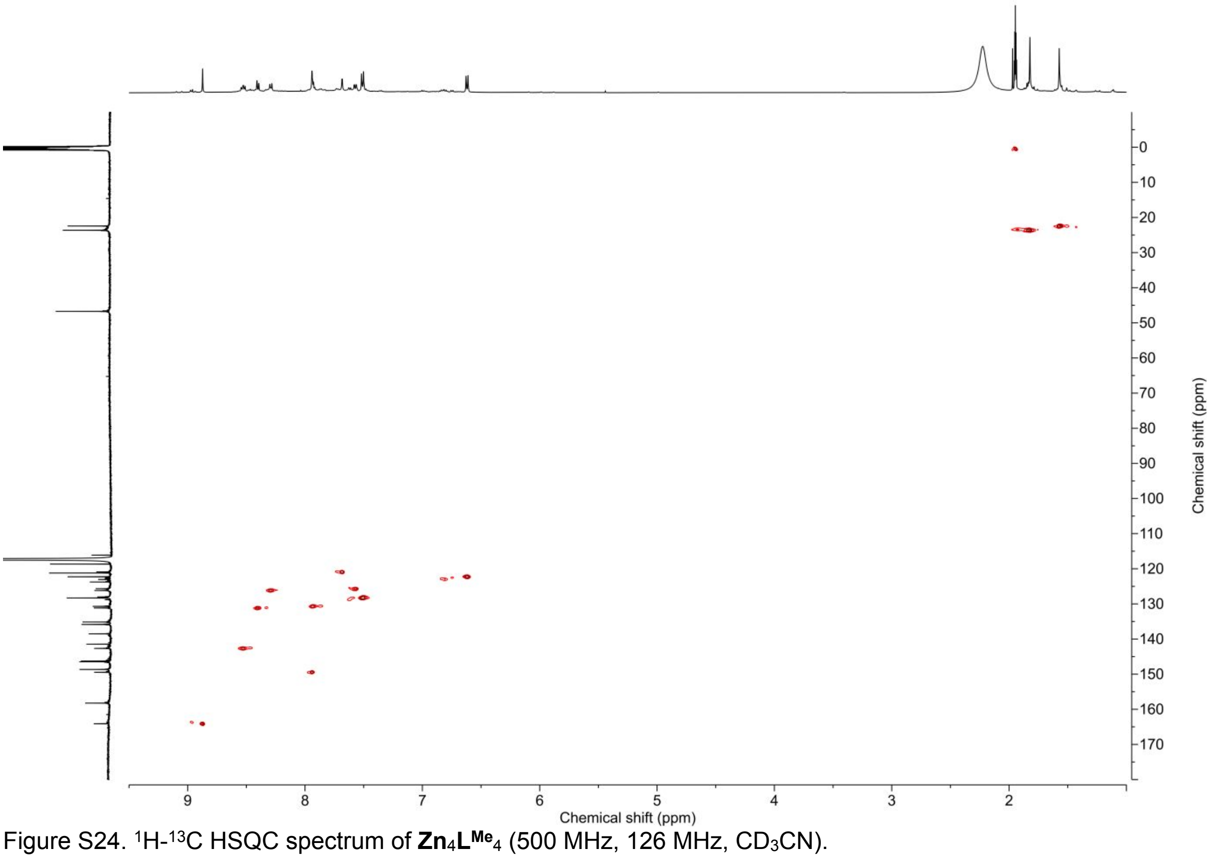

Figure S24. ^{1}H-^{13}C HSQC spectrum of **Zn$_4$L^{Me}**$_4$ (500 MHz, 126 MHz, CD_3CN).

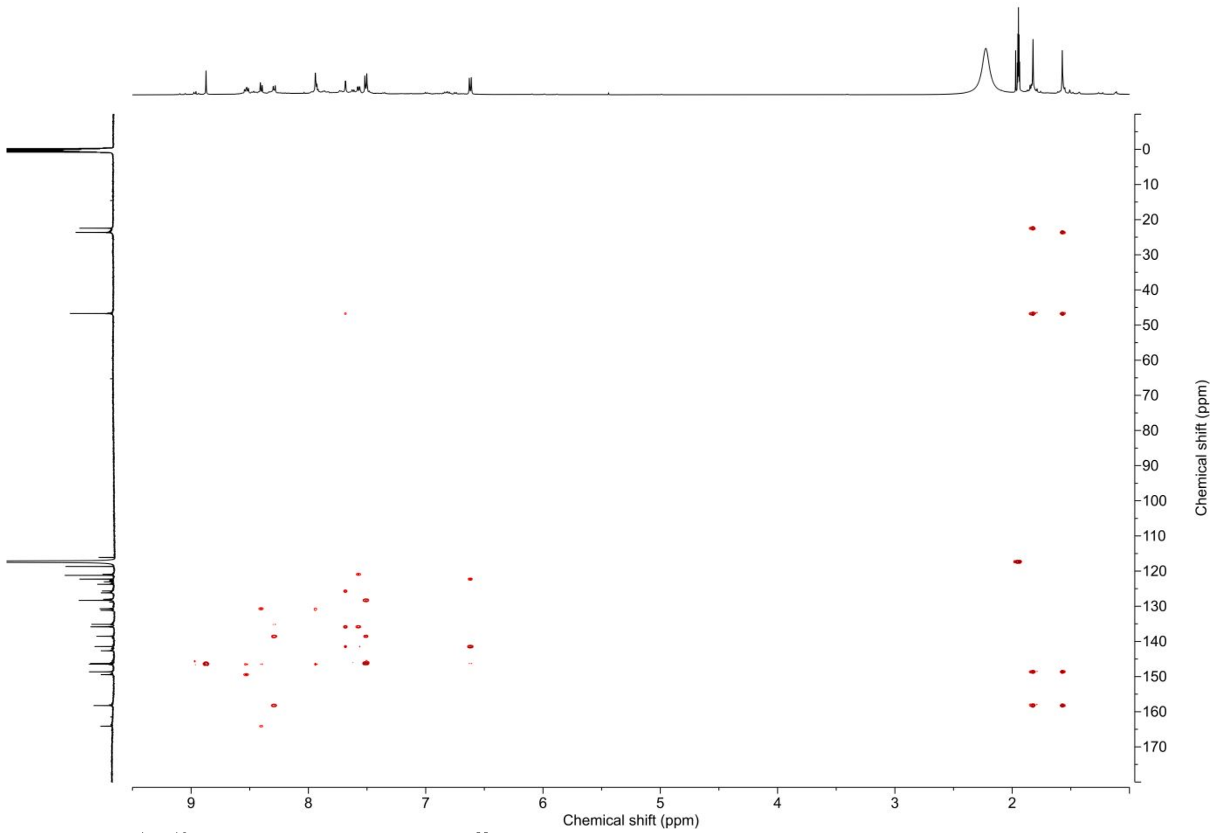

Figure S25. ^{1}H-^{13}C HMBC spectrum of $\mathbf{Zn_4L^{Me}}_4$ (500 MHz, 126 MHz, CD_3CN).

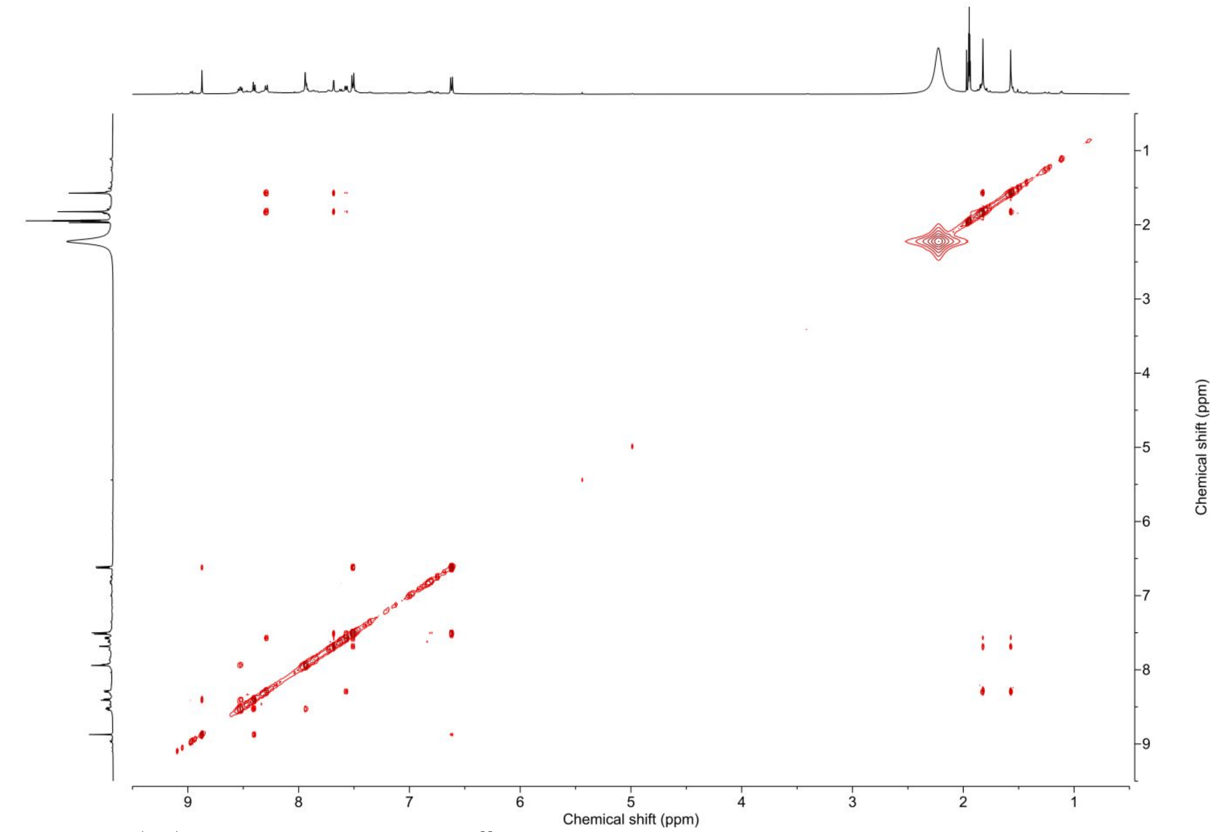

Figure S26. ^{1}H-^{1}H NOESY spectrum of $\mathbf{Zn_4L^{Me}}_4$ (500 MHz, CD_3CN).

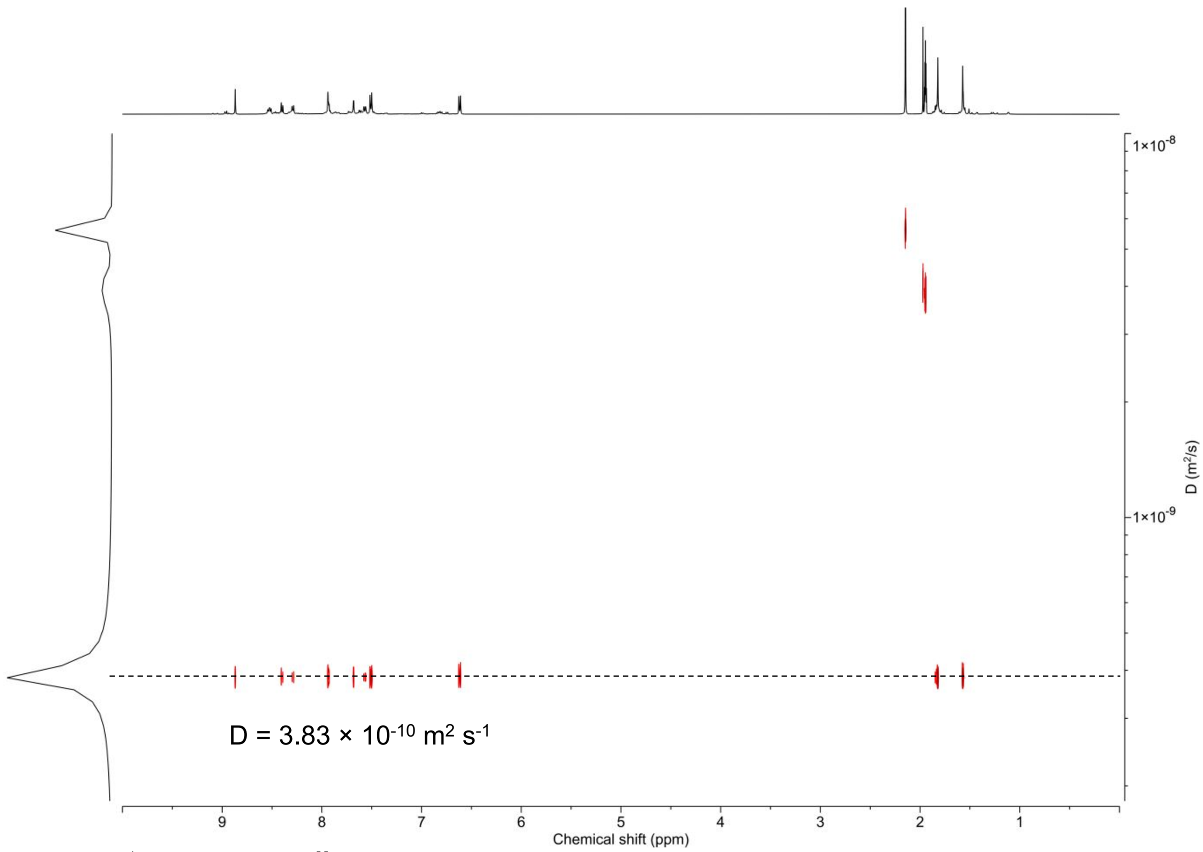

Figure S27. ^{1}H-DOSY of **Zn$_4$L^{Me}$_4$** (500 MHz, CD_3CN).

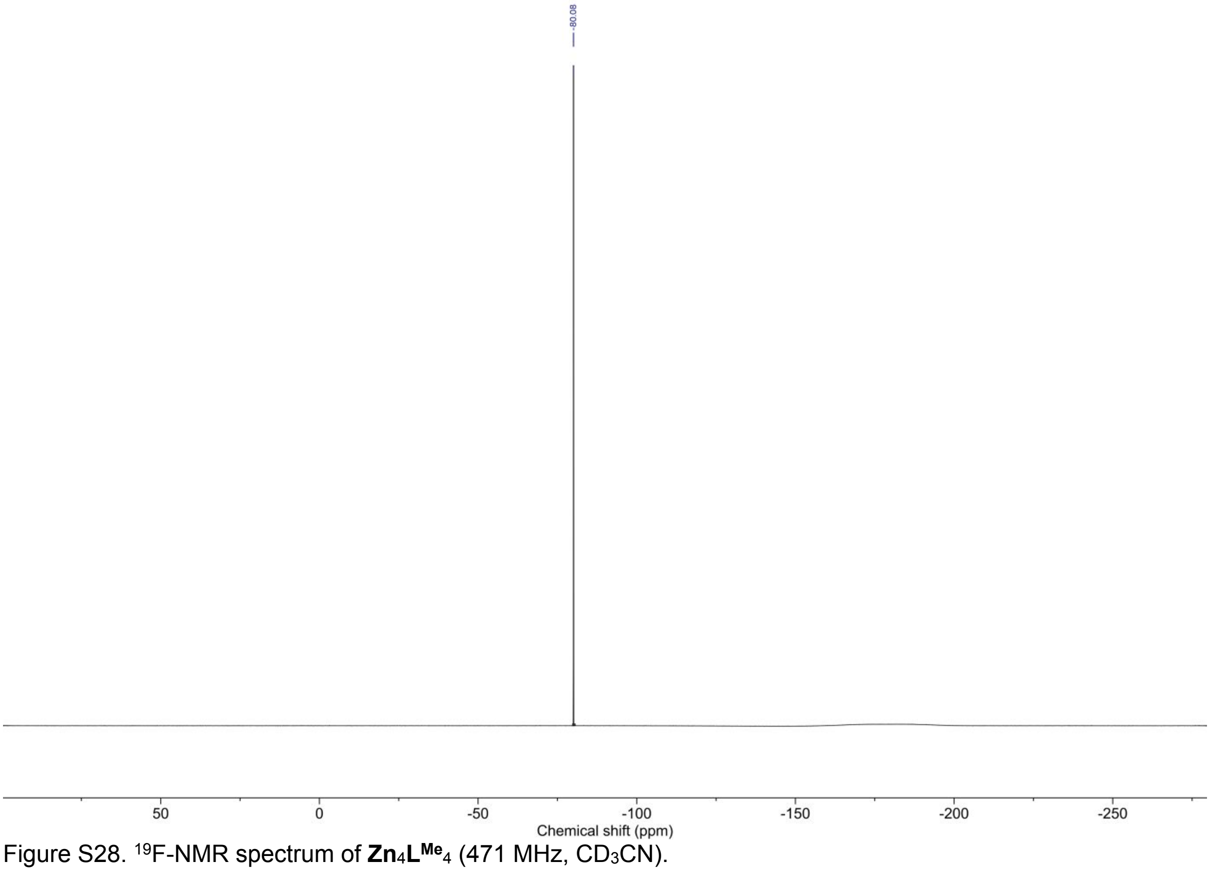

Figure S28. ^{19}F-NMR spectrum of **Zn$_4$L^{Me}$_4$** (471 MHz, CD_3CN).

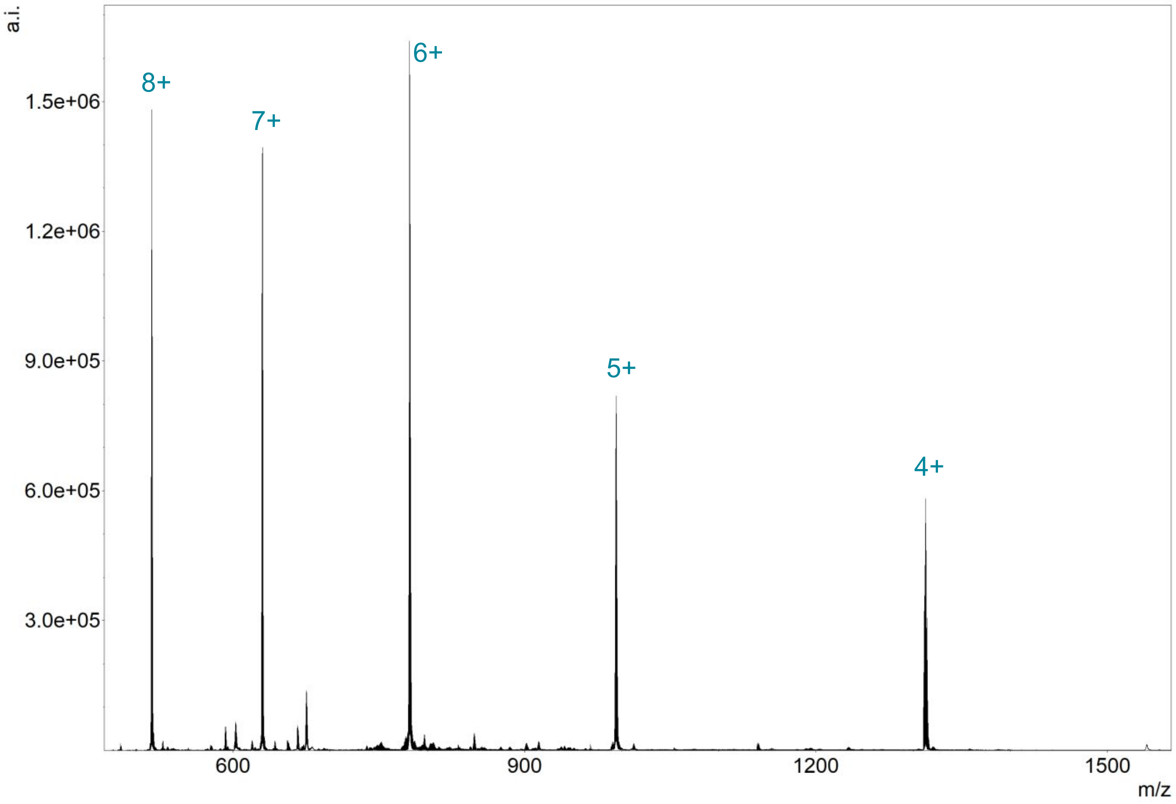

Figure S29. High-resolution ESI-mass spectrum of $\mathbf{Zn_4L^{Me}_4}$.

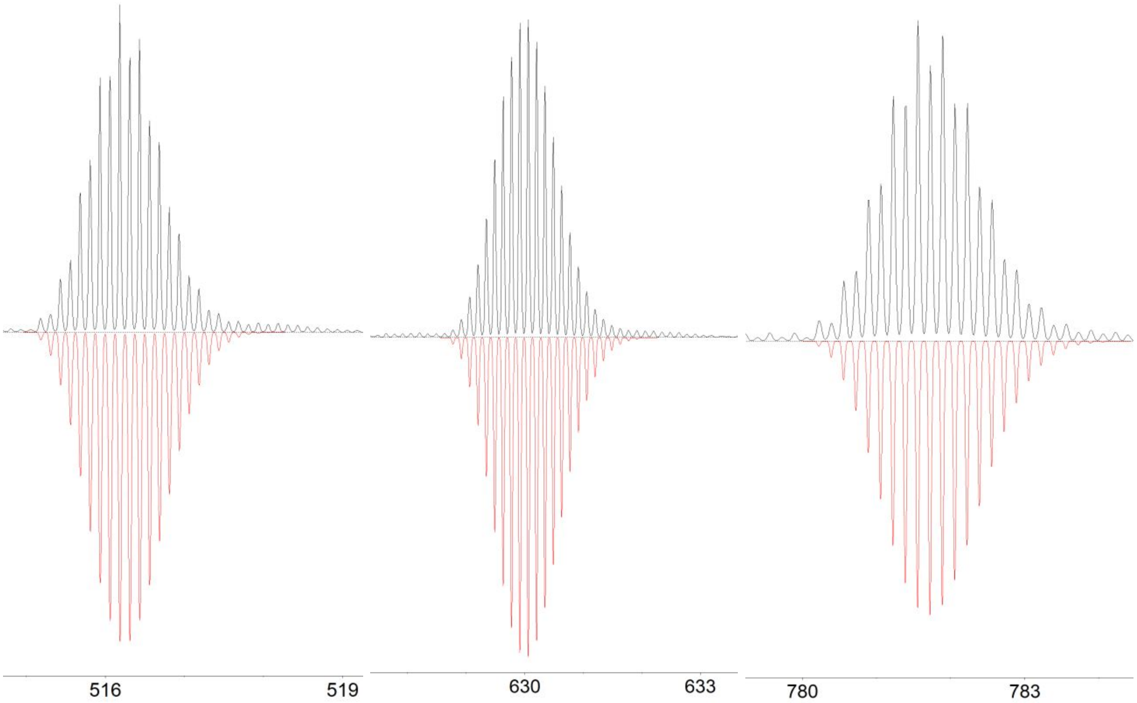

Figure S30. From left to right, peaks corresponding to the 8+, 7+, and 6+ ions of $\mathbf{Zn_4L^{Me}_4}$ (black), overlayed with the calculated m/z (red).

$Fe_4L^{Et}_4$

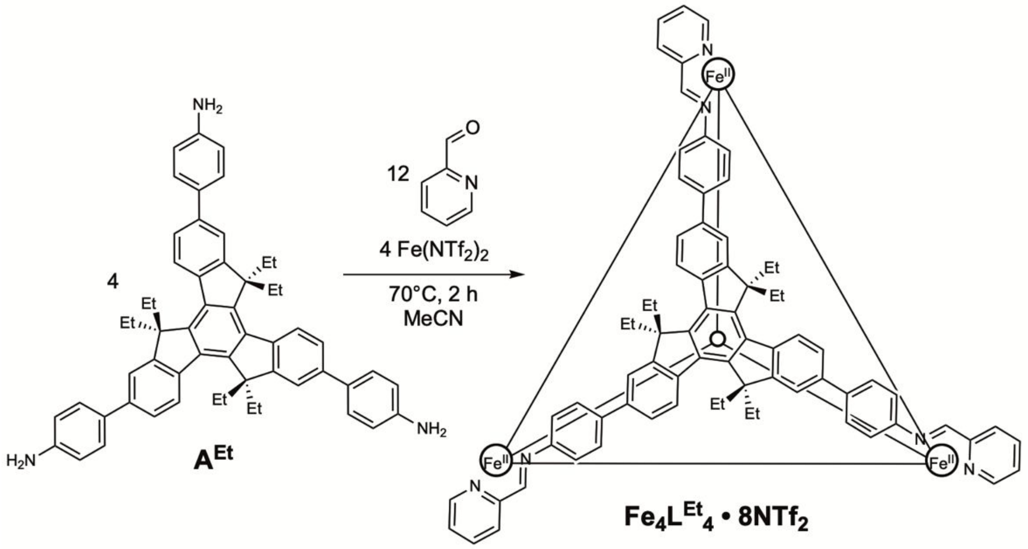

Figure S31. Synthesis of **$Fe_4L^{Et}_4$**.

To a vial, **A^{Et}** (22 mg, 28 µmol), $Fe(NTf_2)_2$ (92% purity, 1 equiv., 19.2 mg, 28.6 µmol) and 2-formylpyridine (3 equiv., 8.2 µl, 9.2 mg, 85.7 µmol) were added. Acetonitrile (2 ml) was added, and the mixture was stirred until clear. The resulting solution was sparged with N_2 for 15 minutes and was then heated to 70 °C for 2 hours. The reaction mixture was allowed to cool to room temperature and was then added to diethyl ether (20 ml). The precipitate was isolated by centrifugation and washed with two more portions of diethyl ether. The resulting solid was dried *in vacuo* to give **$Fe_4L^{Et}_4$** as a black crystalline solid (45 mg, 6.7 µmol, 94% yield).

^{1}H-NMR (500 MHz, CD_3CN) δ 46.80 (s, 1H), 36.22 (s, 1H), 19.83 (s, 1H), 19.69 (s, 1H), 9.14 (s, 2H), 8.43 (d, *J* = 8.3 Hz, 1H), 7.52 (s, 1H), 7.29 (d, *J* = 7.7 Hz, 1H), 6.59 (s, 1H), 3.15 (s, 2H), 2.81 (dq, *J* = 14.5, 7.2 Hz, 1H), 2.60 (dt, *J* = 14.0, 7.4 Hz, 1H), 2.10 – 2.00 (m, 1H), 1.41 (dd, *J* = 14.2, 7.3 Hz, 1H), 0.06 (t, *J* = 7.1 Hz, 3H), -1.26 (t, *J* = 7.3 Hz, 3H).

^{13}C-NMR (126 MHz, CD_3CN) δ 168.0, 158.2, 152.6, 146.3, 146.0, 142.4, 137.6, 133.6, 129.4, 127.0, 124.7, 123.4, 122.1, 119.6, 57.3, 30.3, 30.1, 8.7, 7.9.

Most protons in the ^{1}H-NMR spectrum could be assigned according to the 2D NMR spectra below, but the broad peaks at 3.17, 36.2 and 46.9 ppm did not show 2D correlations. These peaks were therefore assigned by correlating their T1 values with the distances of the respective protons from the paramagnetic Fe centres, in line with previous work.[3, 4] The peaks at 19.71 and 19.86 ppm showed NOESY correlations to proton n (see below), and the match between their T1 values and distances from the Fe centre was consistent with the assignment. The above five peaks have substantially shorter T1 relaxation times than the rest of the peaks in the spectrum and correspond to the five proton environments closest to the spin-crossover Fe^{II} centres. In addition, the shifts of the above peaks in the low-temperature ^{1}H-NMR spectra fit the expected values for a low-spin cage.

T1 values were measures using the t1ir pulse program and the data was processed using Bruker Dynamics Center. Two separate experiments were run on the diamagnetic (-2 to 10 ppm) and the paramagnetic regions (19 to 50 ppm) of the spectrum.

Table S1. T1 relaxation times for $\mathbf{Fe_4L^{Et}_4}$.

chemical shift [ppm]	T1 [s]	error	assignment	distance from Fe (Å)
-1.26	0.129	0.010	d	
0.05	0.573	0.029	a	
0.06	0.576	0.030	a	
0.07	0.572	0.031	a	
1.40	0.337	0.014	e	
1.42	0.334	0.014	e	
2.02	0.371	0.015	c	
2.04	0.365	0.015	c	
2.05	0.353	0.015	c	
2.06	0.350	0.014	c	
2.09	0.366	0.020	c	
2.60	0.301	0.016	f	
2.61	0.297	0.017	f	
2.82	0.364	0.025	b	
2.79	0.367	0.023	b	
2.82	0.357	0.025	b	
2.83	0.340	0.032	b	
3.15	**0.010**	0.001	**k**	**3.4**
6.59	0.332	0.006	g	
7.29	0.514	0.012	i	
7.30	0.515	0.013	i	
7.52	0.172	0.004	n	
8.42	0.403	0.007	h	
8.44	0.404	0.007	h	
9.14	0.147	0.003	j	
19.69	**0.06997**	0.00010	**o**	**5.04**
19.83	**0.05968**	0.00007	**m**	**4.86**
36.22	**0.00502**	0.00005	**p**	**3.12**
46.80	**0.01258**	0.00013	**l**	**3.7**

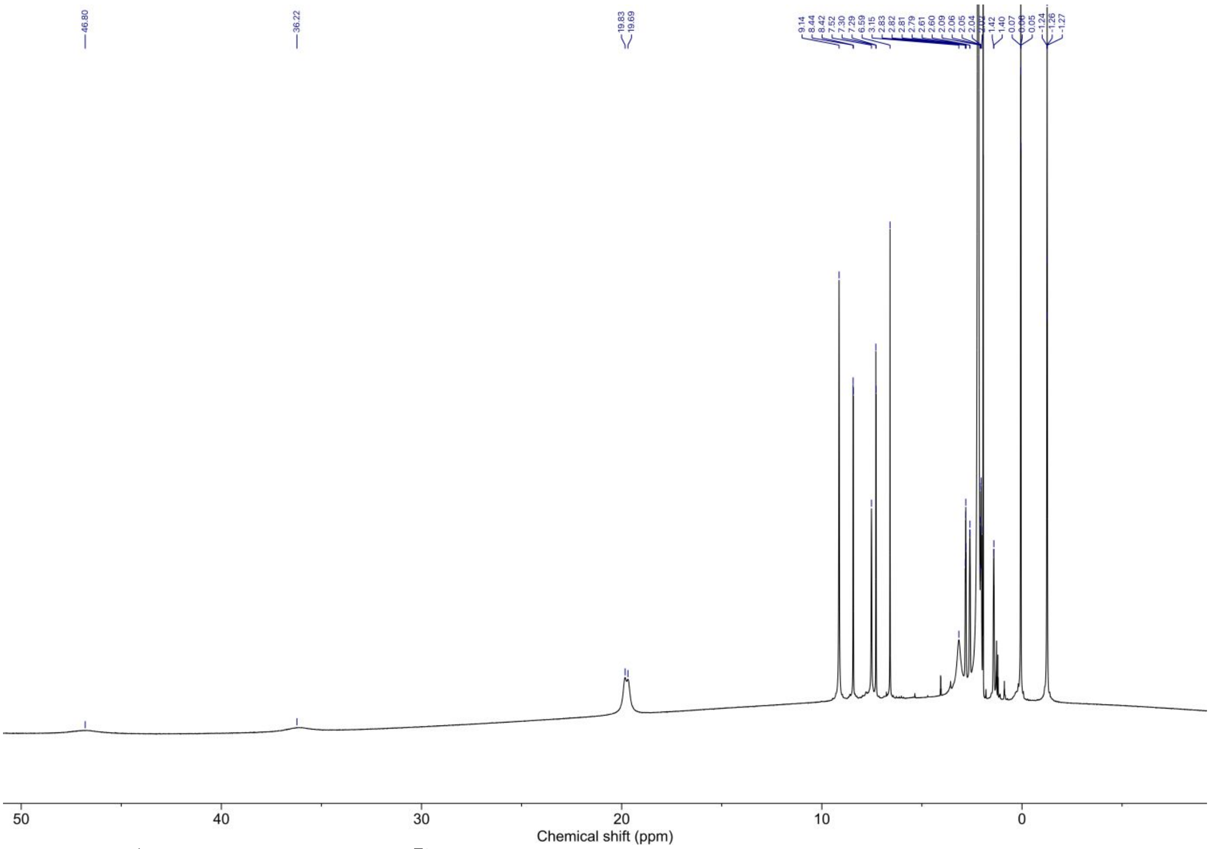

Figure S32. ^{1}H-NMR spectrum of $\mathbf{Fe_4L^{Et}_4}$ (500 MHz, CD_3CN).

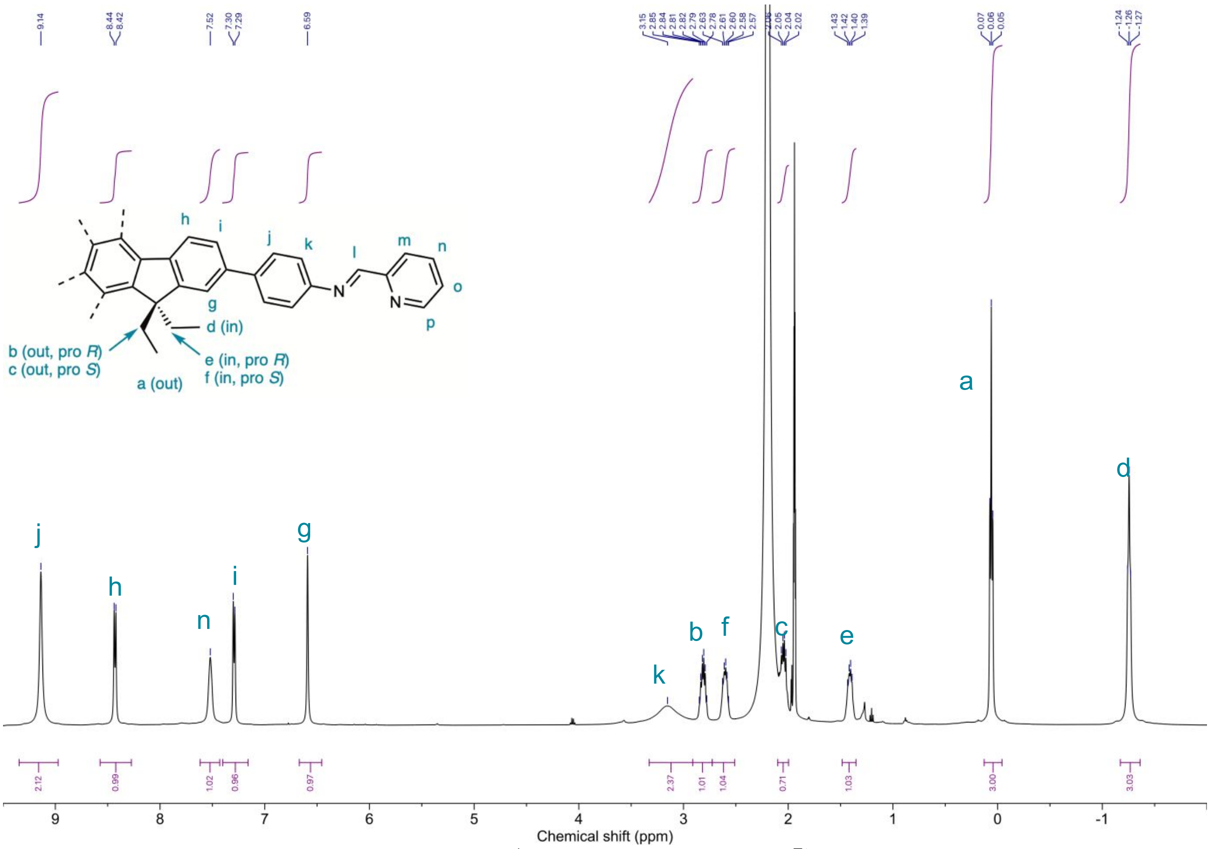

Figure S33. Assigned diamagnetic region of the ^{1}H-NMR spectrum of $\mathbf{Fe_4L^{Et}_4}$ (500 MHz, CD_3CN).

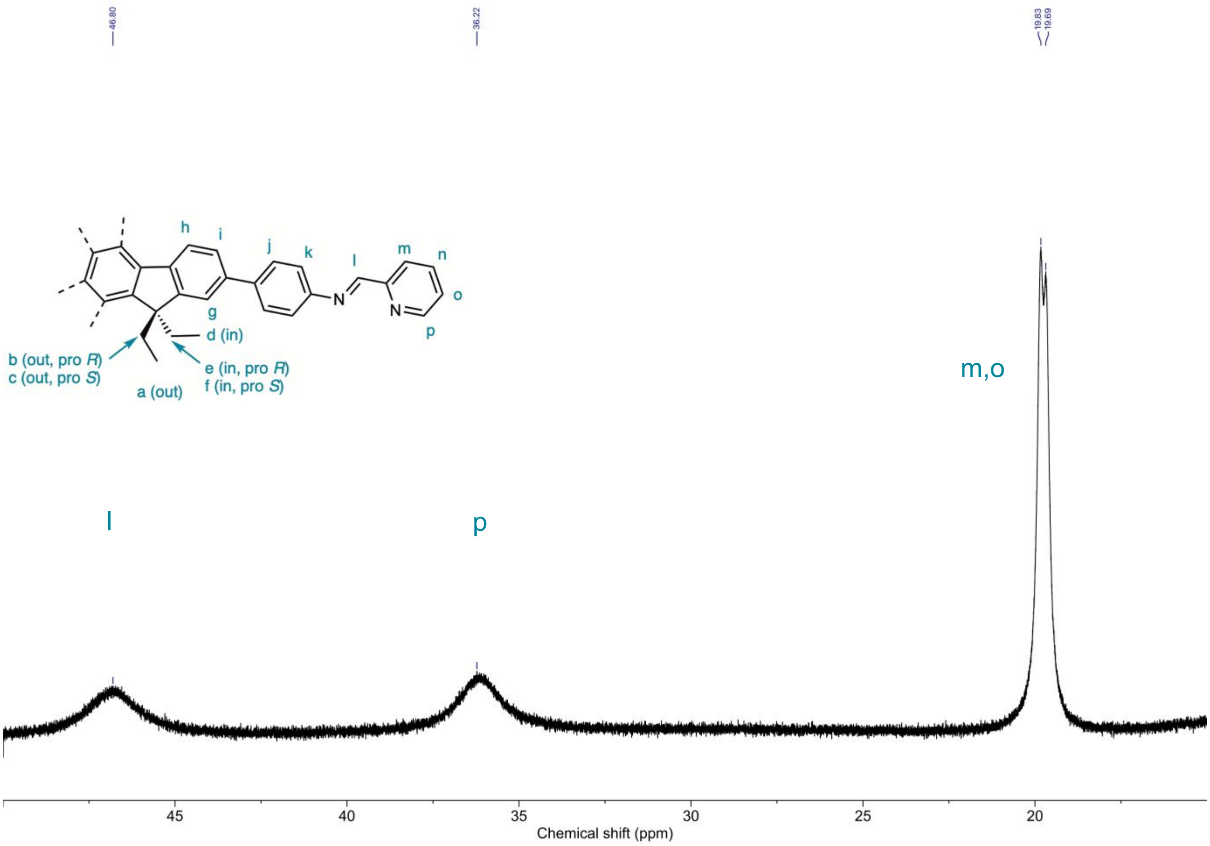

Figure S34. Assigned paramagnetic region of the ^{1}H-NMR spectrum of **Fe$_4$L^{Et}$_4$** (500 MHz, CD_3CN).

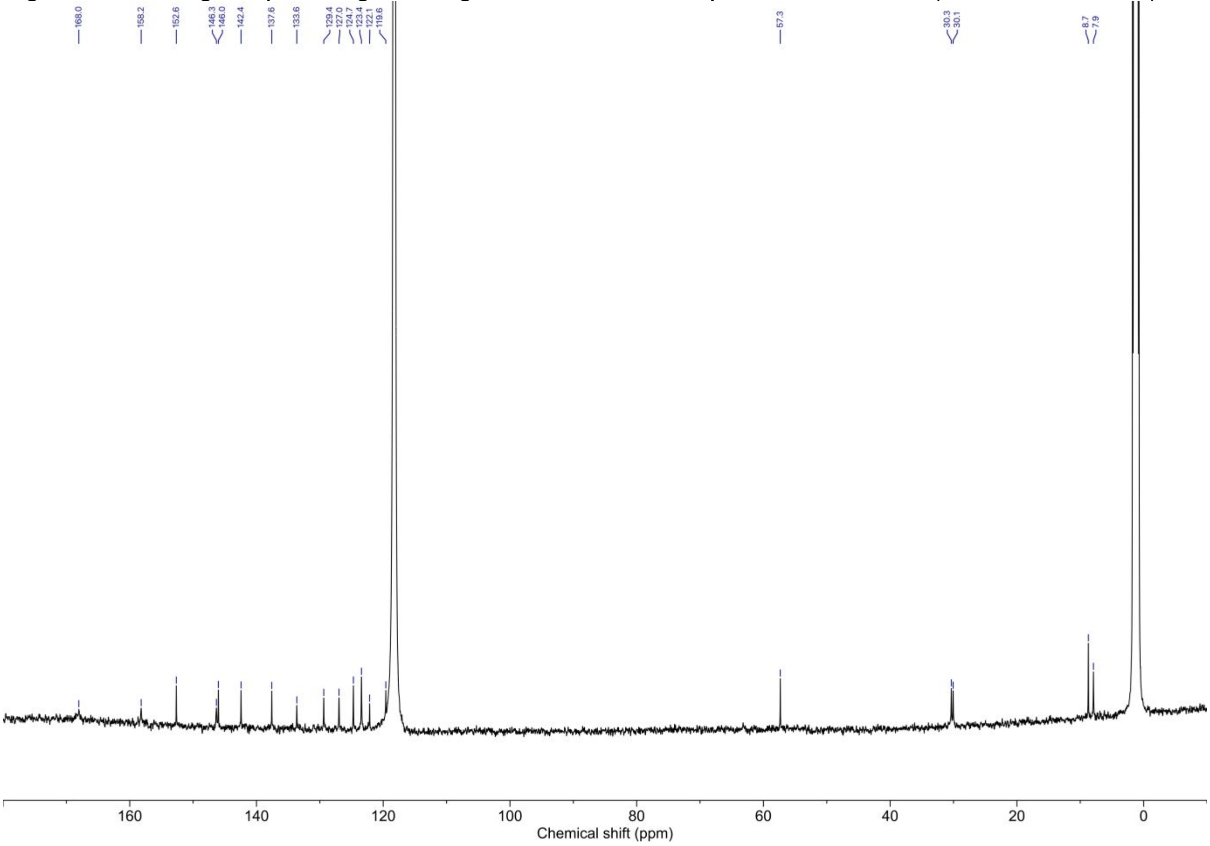

Figure S35. ^{13}C-NMR spectrum of **Fe$_4$L^{Et}$_4$** (126 MHz, CD_3CN).

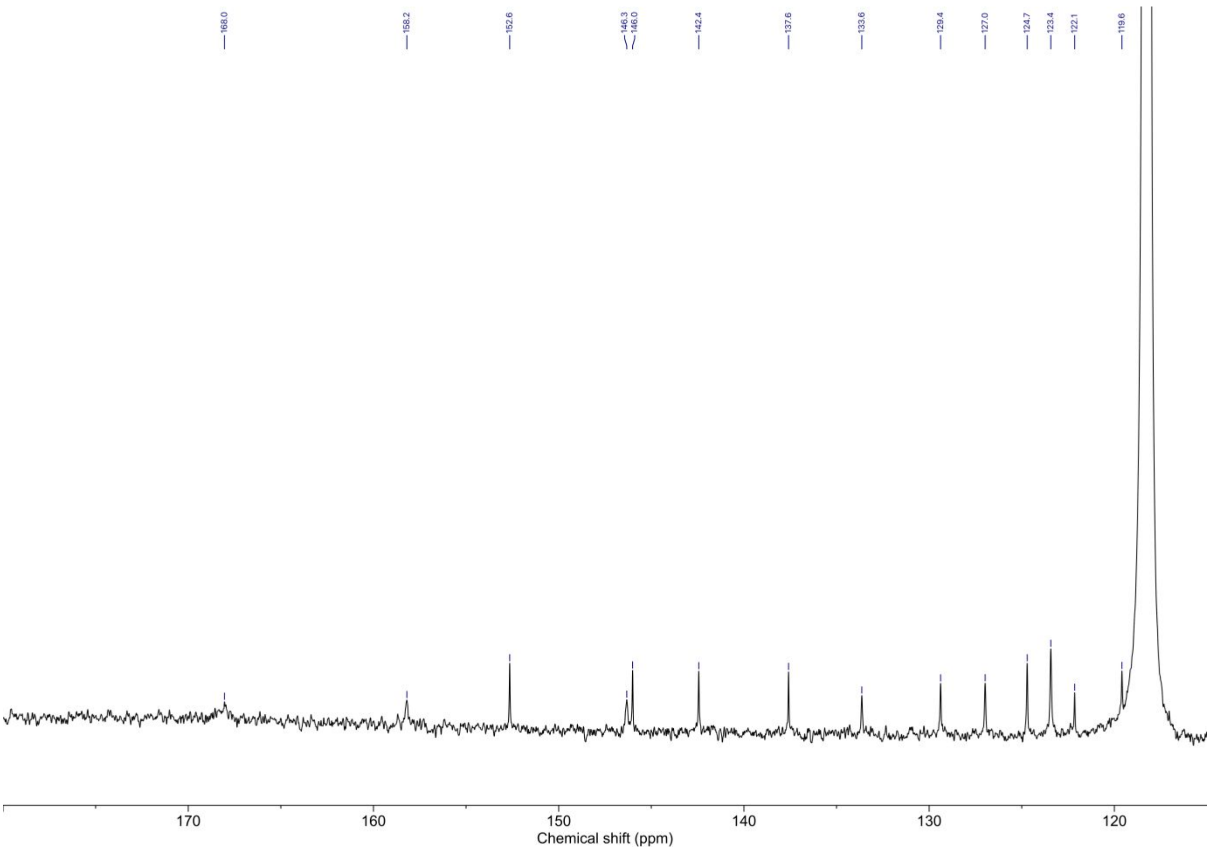

Figure S36. Aromatic region of the ^{13}C-NMR spectrum of $\mathbf{Fe_4L^{Et}}_4$ (126 MHz, CD_3CN).

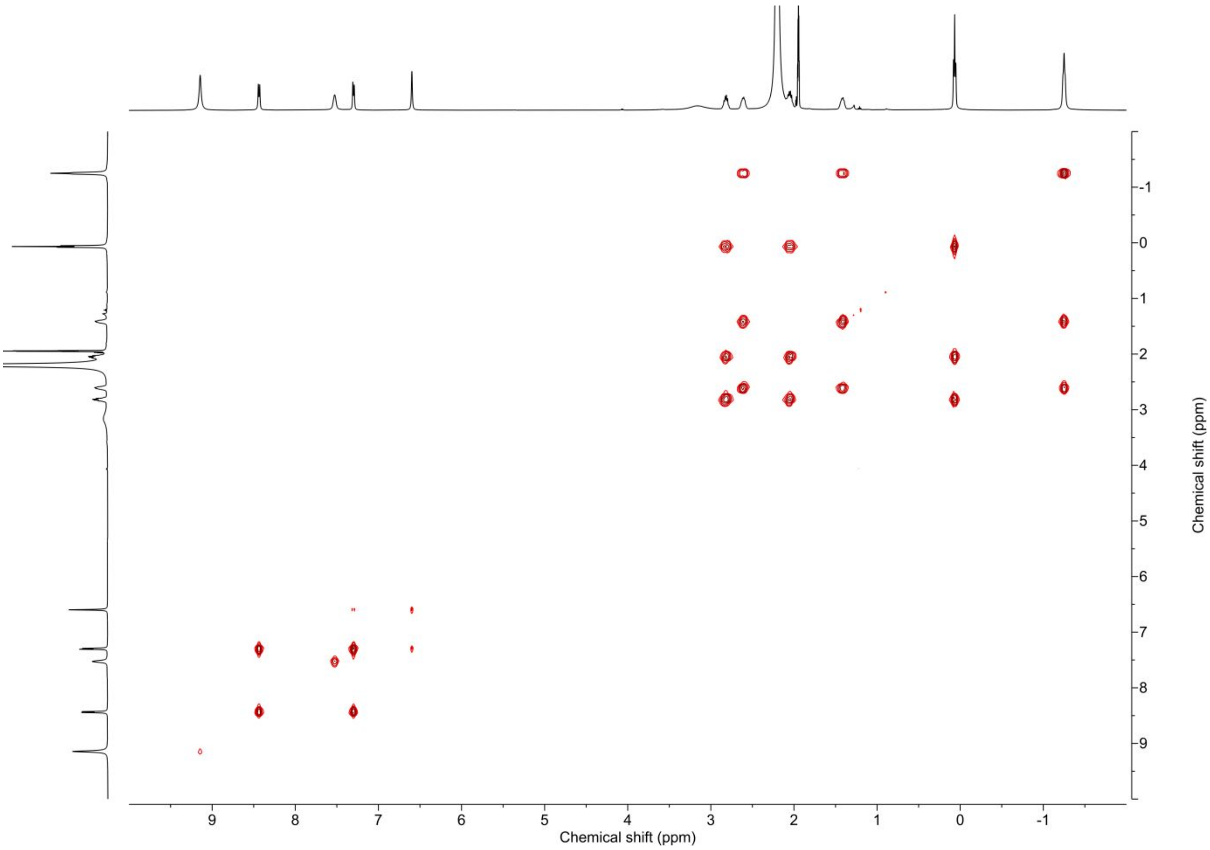

Figure S37. ^{1}H-^{1}H DQF-COSY spectrum of $\mathbf{Fe_4L^{Et}}_4$ (500 MHz, CD_3CN).

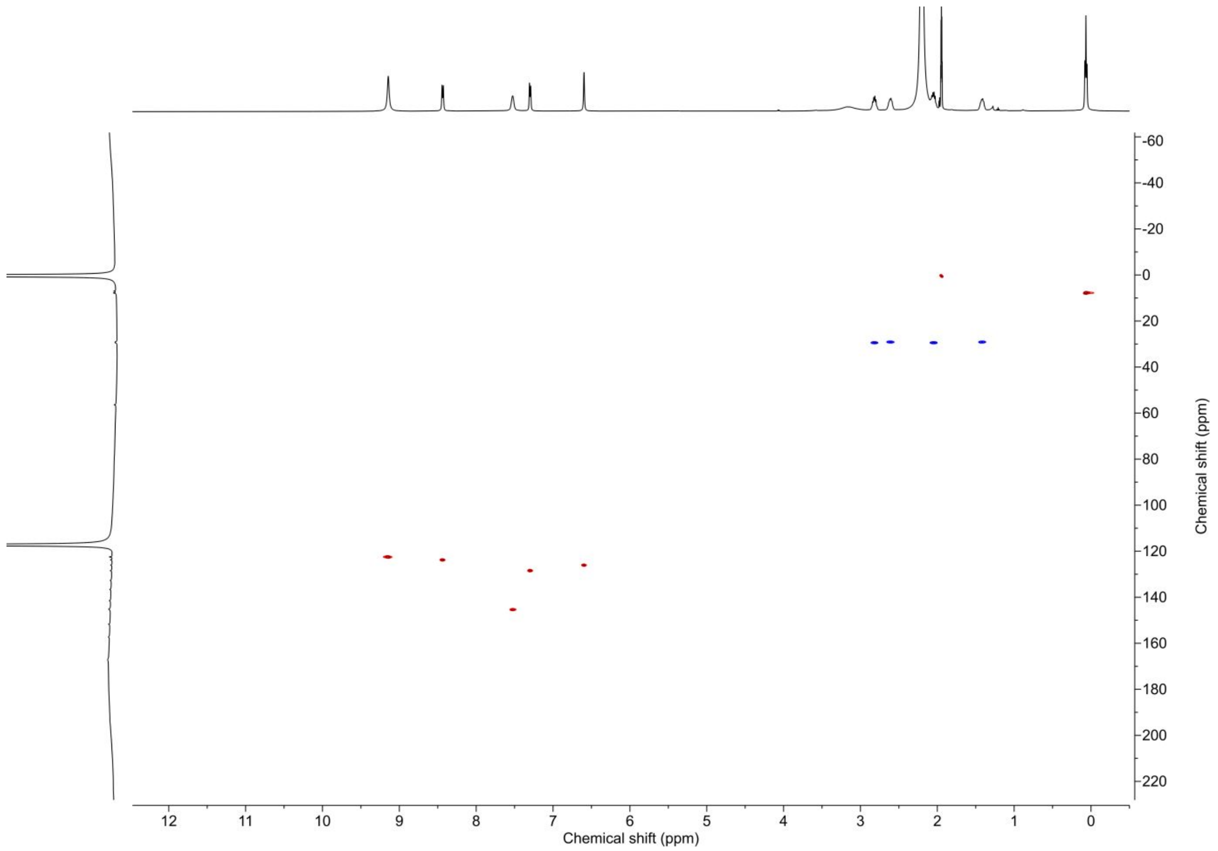

Figure S38. ^{1}H-^{13}C HSQC spectrum of $\mathbf{Fe_4L^{Et}}_4$ with DEPT135 editing (500 MHz, 126 MHz, CD_3CN).

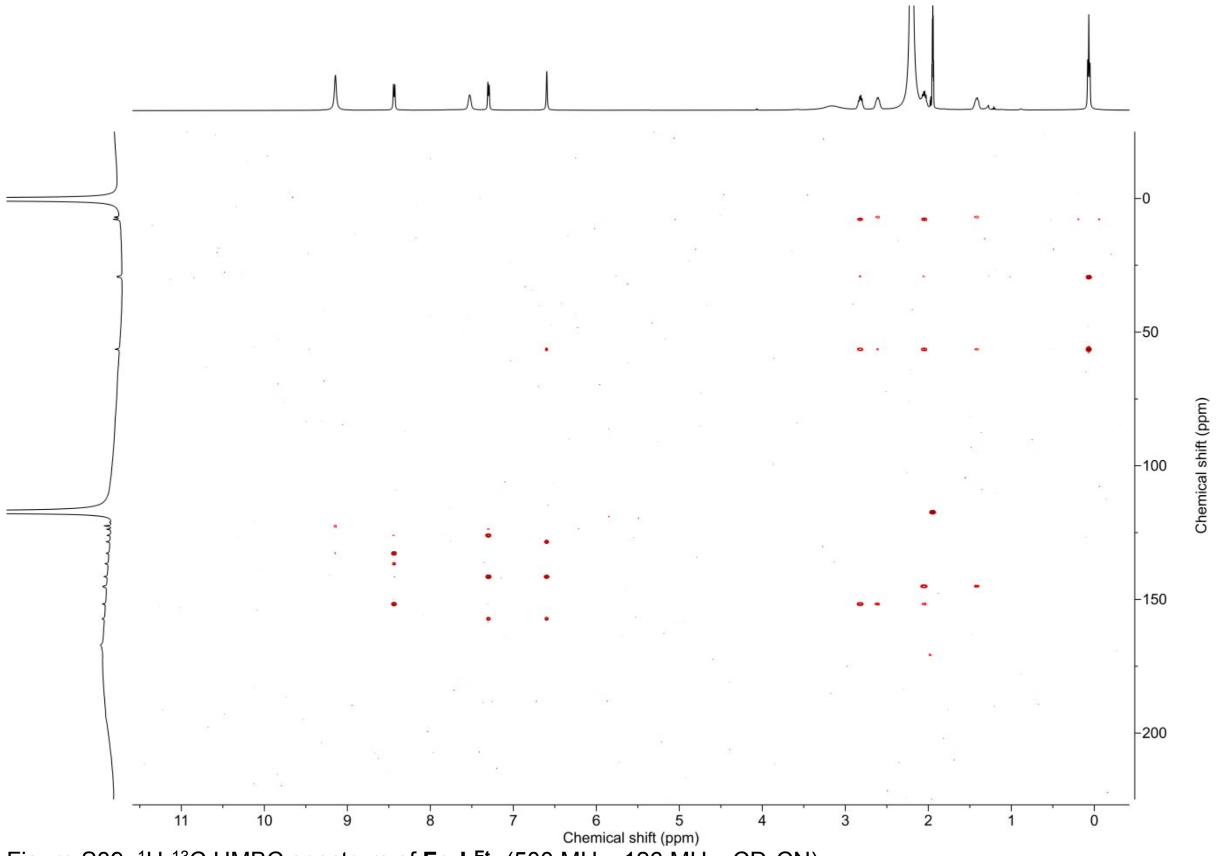

Figure S39. ^{1}H-^{13}C HMBC spectrum of $\mathbf{Fe_4L^{Et}}_4$ (500 MHz, 126 MHz, CD_3CN).

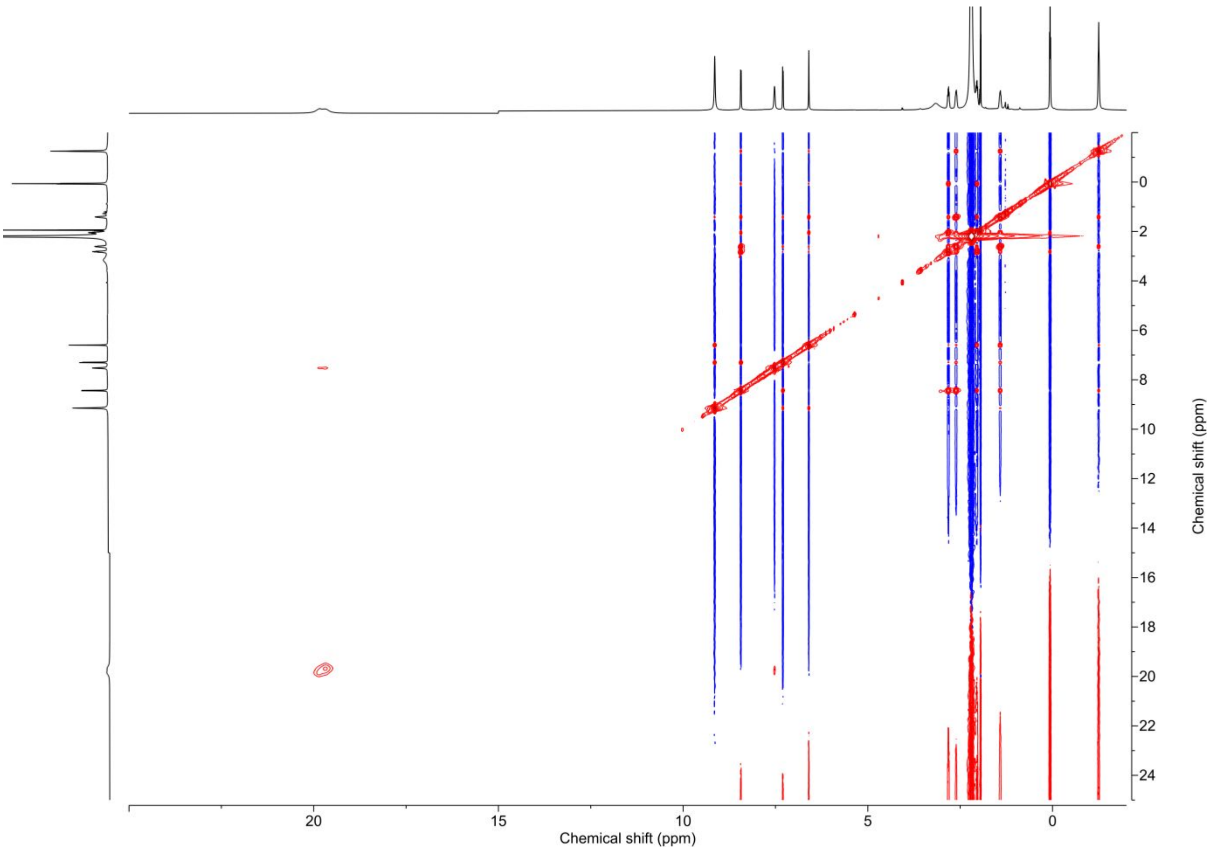

Figure S40. ^{1}H-^{1}H NOESY spectrum of **Fe$_4$L^{Et}$_4$** (500 MHz, CD_3CN).

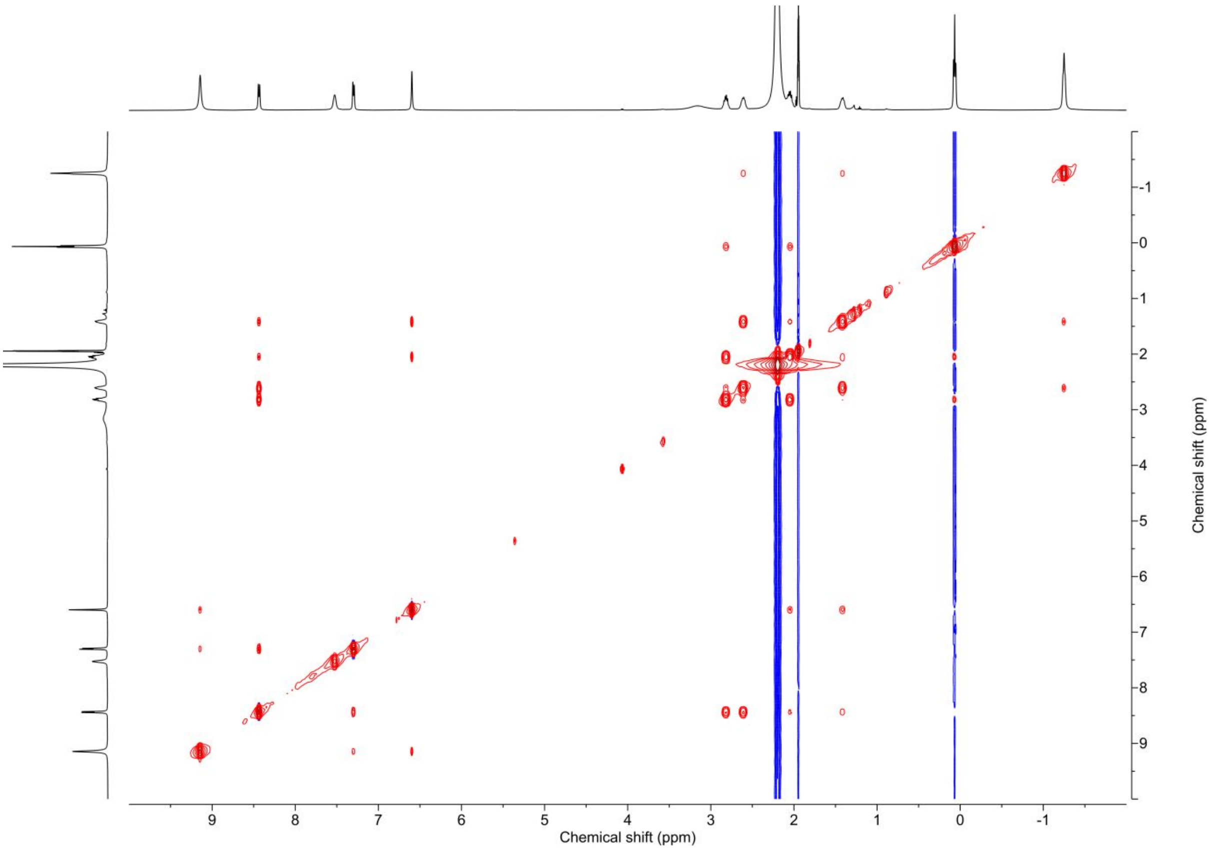

Figure S41. Diamagnetic region of the ^{1}H-^{1}H NOESY spectrum of **Fe$_4$L^{Et}$_4$** (500 MHz, CD_3CN).

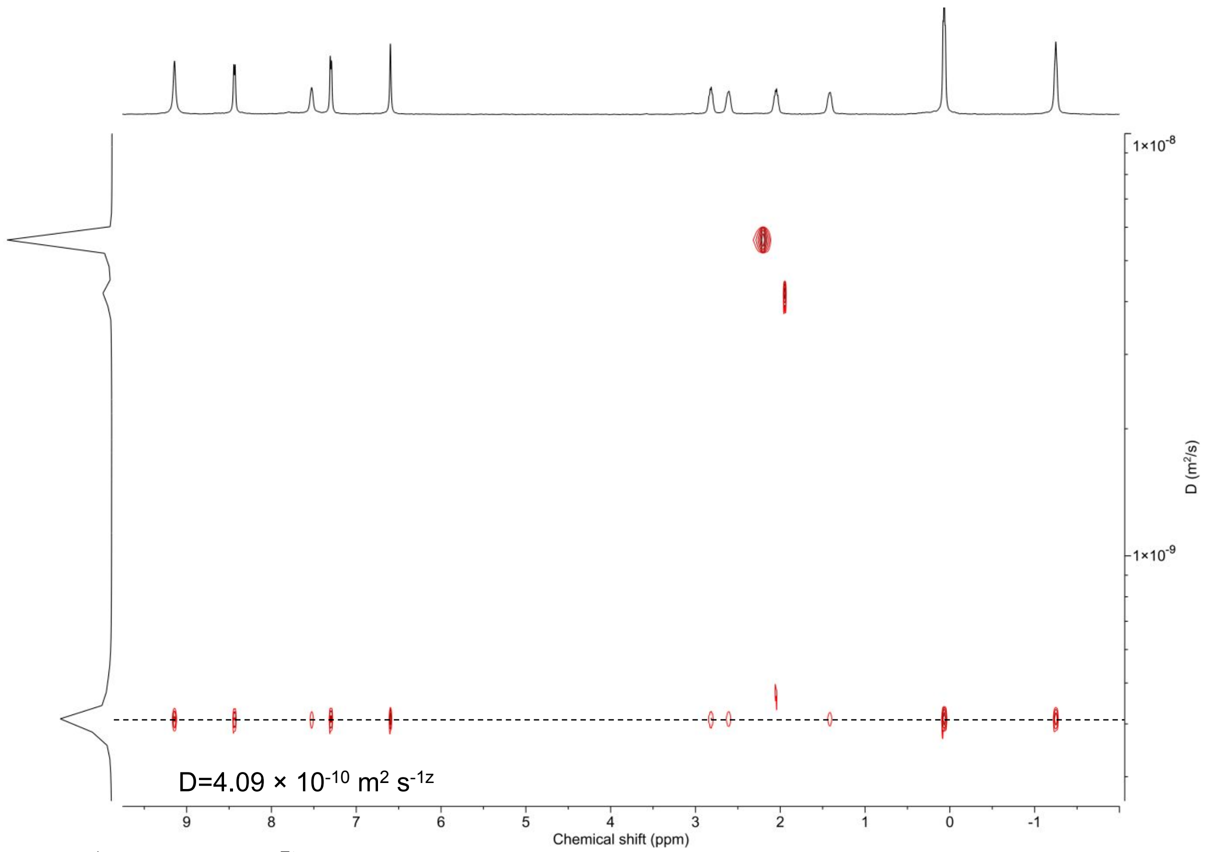

Fig.S42. ^{1}H-DOSY of **$Fe_4L^{Et}{}_4$** (500 MHz, CD_3CN).

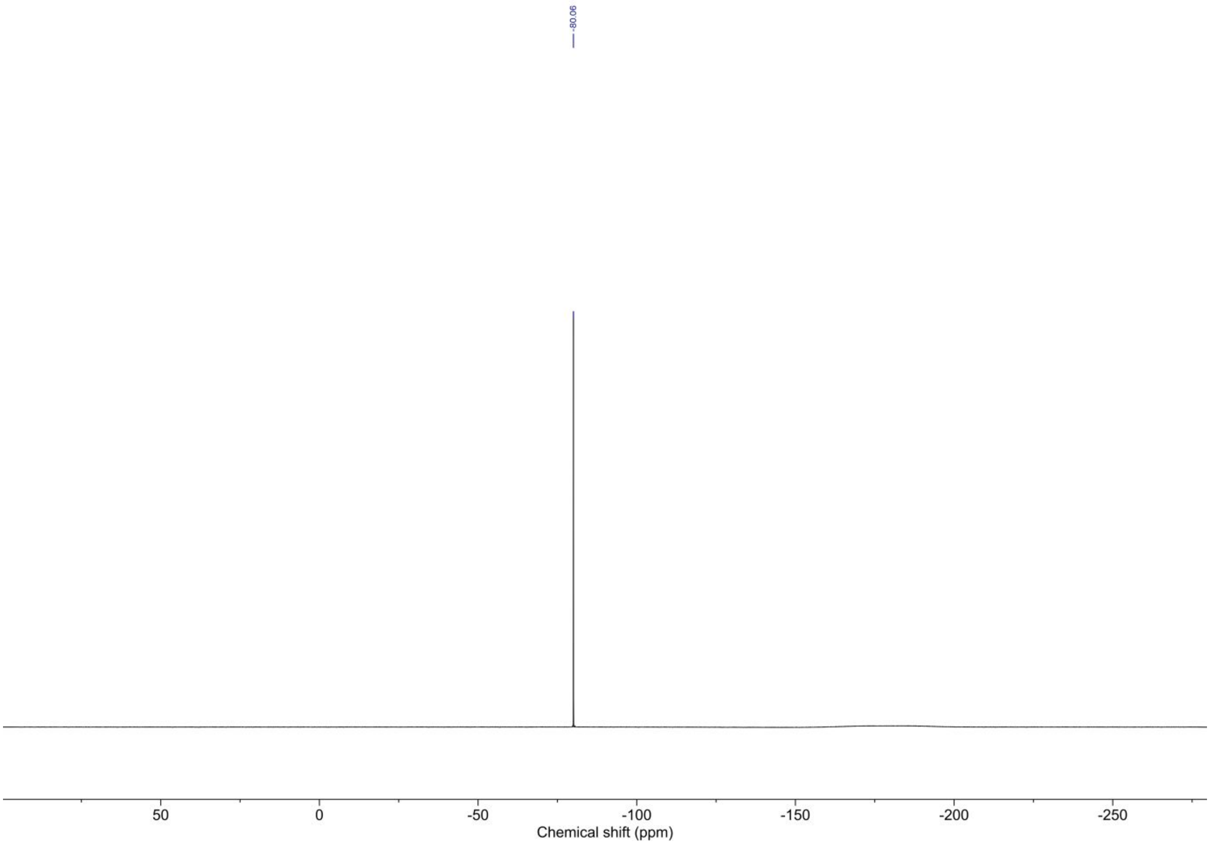

Figure S43. ^{19}F-NMR spectrum of **$Fe_4L^{Et}{}_4$** (471 MHz, CD_3CN).

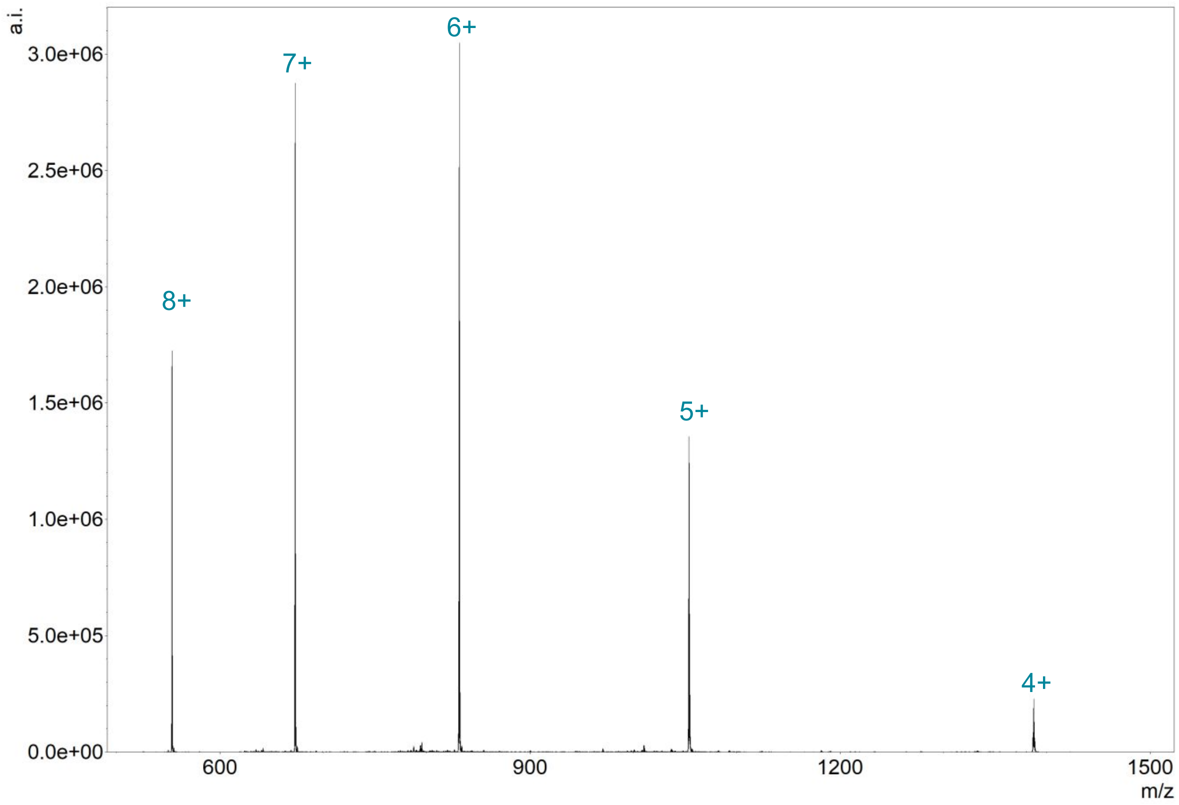

Figure S44. High-resolution ESI-mass spectrum of $\mathbf{Fe_4L^{Et}_4}$.

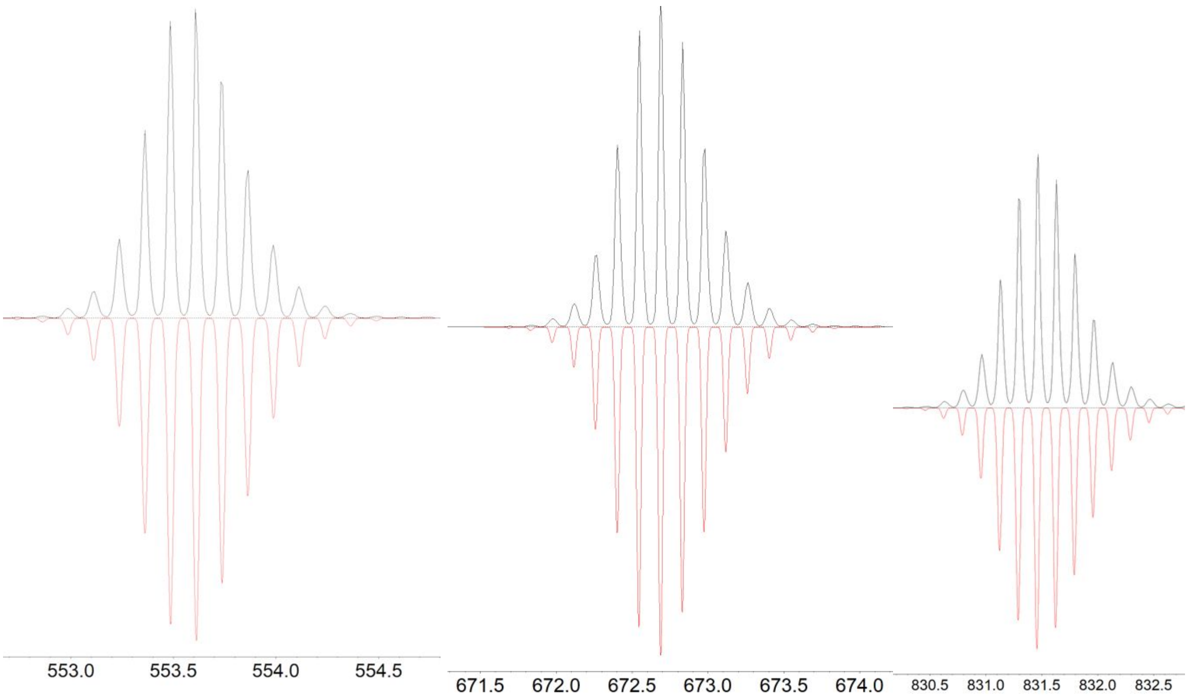

Figure S45. From left to right, peaks corresponding to the 8+, 7+, and 6+ ions of $\mathbf{Fe_4L^{Et}_4}$ (black), overlayed with the calculated m/z (red).

4. Spin-crossover studies on $Fe_4L^{Et}_4$

The chemical shifts of the imine proton in the variable-temperature ^{1}H-NMR spectra of **$Fe_4L^{Et}_4$** (Figure S47) were fitted to Eq. 1:[5]

$$\delta_{imine} = \delta_{imine,LS} + \frac{C}{T + Te^{\frac{\Delta H - T\Delta S}{RT}}} (1)$$

Eq 1. neglects cooperativity between the different Fe centres, and models an equilibrium between an all-low-spin and all-high-spin cage.[4] δ_{imine} is the chemical shift of the imine proton at a given temperature, $\delta_{imine,LS}$ is the shift in the low-spin state, which was approximated as the shift of imine peak in the corresponding Zn cage (**$Zn_4L^{Et}_4$**), C is a constant, T is the temperature (in K), ΔH and ΔS are the enthalpy and entropy change in the low-spin to high-spin transition, respectively, and R is the gas constant.
The data was fitted using Origin 2020.

Table S2. Thermodynamic values for the spin-crossover equilibrium of **$Fe_4L^{Et}_4$**.

Parameter	Value	Standard Error
$C\ (K)$	$5.40 \cdot 10^4$	$0.09 \cdot 10^4$
$\Delta H\ (\frac{kJ}{mol})$	22.60	0.16
$\Delta S\ (\frac{J}{molK})$	64.73	0.69

The ΔH and ΔS values extracted were used to calculate the population of the low- and high-spin states (Eqs. 2-3):

$$\gamma_{LS} = \frac{1}{1 + e^{-\frac{\Delta H - T\Delta S}{RT}}} (2)$$

$$\gamma_{HS} = 1 - \gamma_{LS}\ (3)$$

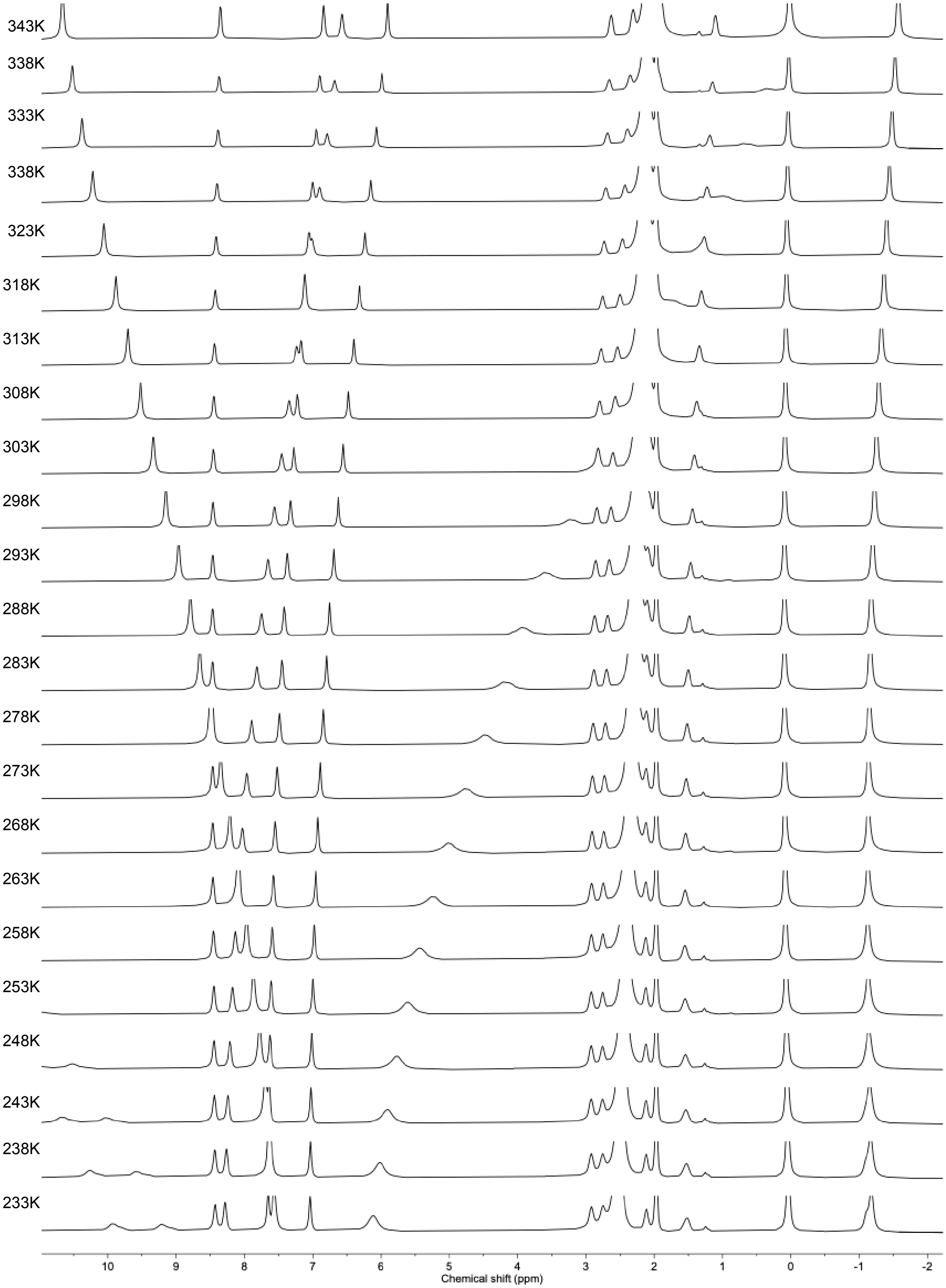

Figure S46. The region between 11 and –2 ppm of the ^{1}H-NMR spectra of **$Fe_4L^{Et}_4$** (500 MHz, CD_3CN) at temperatures from 233 to 343 K.

343 K

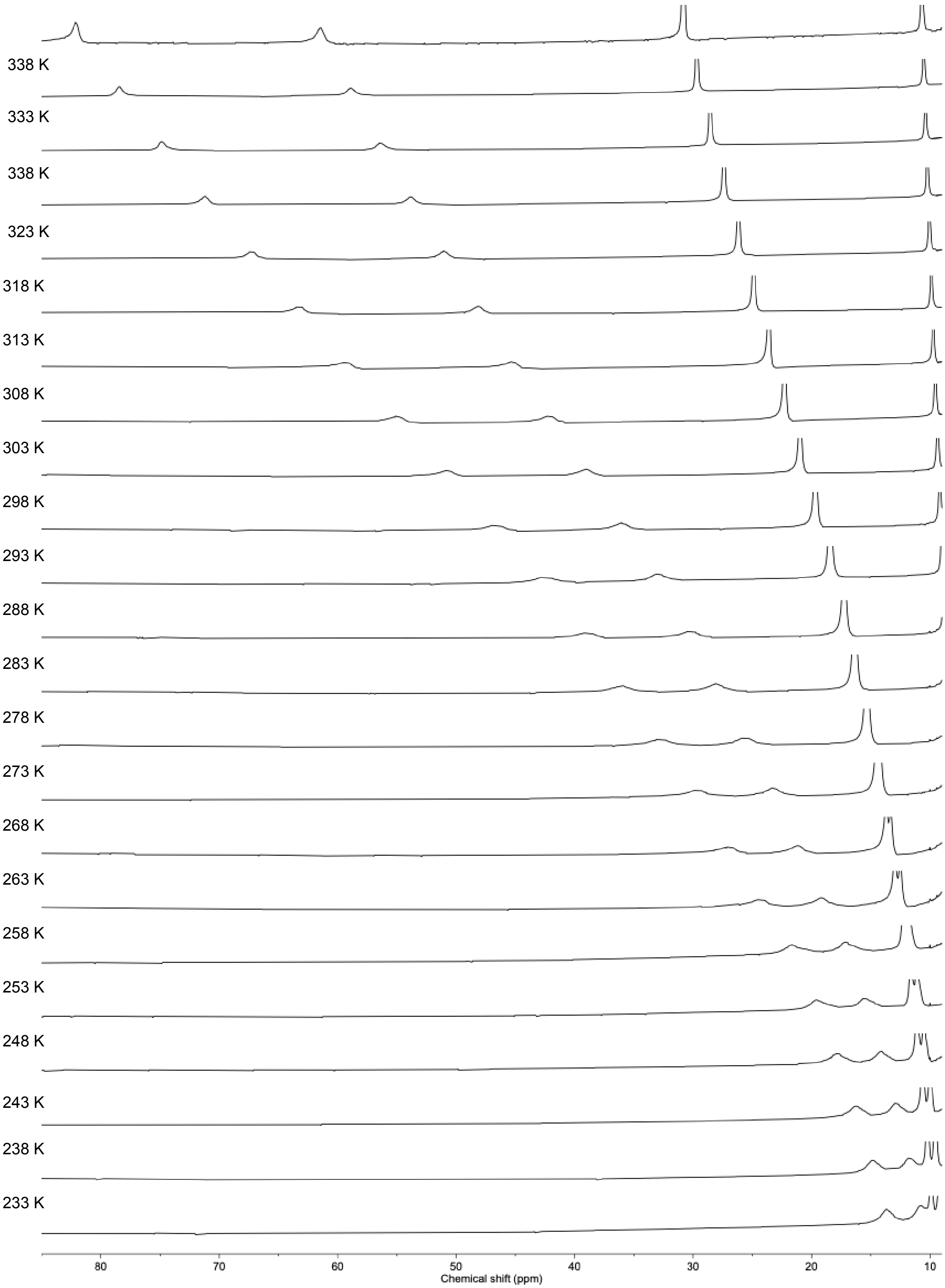

Figure S47. The region between 85 and 10 ppm of the ^{1}H-NMR spectra of $\mathbf{Fe_4L^{Et}_4}$ (500 MHz, CD_3CN) at temperatures from 233 to 343 K.

5. X-ray crystallography

Data were collected at Beamline I19 of Diamond Light Source employing silicon double crystal monochromated synchrotron radiation (0.6889 Å, Dectris PILATUS 2M detector) with ω and ψ scans at 100(2) K.[6] Data integration and reduction were undertaken with Xia2.[7-9] Subsequent computations were carried out using the WinGX-32[10] and ShelXle[11] graphical user interfaces. Empirical absorption corrections were applied to the data using DIALS.[9] The structures were solved by direct methods using SHELXT[12] then refined and extended with SHELXL.[13] In general, non-hydrogen atoms with occupancies greater than 0.5 were refined anisotropically. Carbon-bound hydrogen atoms were included in idealised positions and refined using a riding model. The methyl groups were modelled from electron density using HFIX 137. Disorder was modelled using standard crystallographic methods including constraints, restraints and rigid bodies where necessary. Crystallographic data along with specific details pertaining to the refinement follow. Crystallographic data have been deposited with the CCDC (2497193-2497195).

$Fe_4L^{Me}{}_4$

$[Fe_4L^{Me}{}_4]\cdot 8PF_6\cdot 9C_6H_6\cdot 3CH_3CN$ [+ solvent]

Crystallization conditions:
The crystals of $[Fe_4L^{Me}{}_4]\cdot 8PF_6\cdot 9C_6H_6\cdot 3CH_3CN$ [+ solvent] were grown by diffusion of benzene into an acetonitrile solution of $[Fe_4L^{Me}{}_4]\cdot 8NTf_2$ containing excess $TBAPF_6$.

Formula $C_{336}H_{279}F_{48}Fe_4N_{27}P_8$, *M* 6078.00, Orthorhombic, space group I 2 2 2 (#23), *a* 21.5812(3), *b* 31.3053(3), *c* 32.6045(3) Å, *V* 22027.8(4) Å^3, D_c 0.916 g cm^{-3}, *Z* 2, crystal size 0.050 by 0.040 by 0.030 mm, colour purple, habit block, temperature 100(2) Kelvin, λ(Synchrotron) 0.6889 Å, μ(Synchrotron) 0.201 mm^{-1}, T(Analytical)$_{min,max}$ 0.7859447828428082, 1.0, $2\theta_{max}$ 58.95, *hkl* range -30 24, -44 33, -46 45, *N* 96576, N_{ind} 32426(R_{merge} 0.0248), N_{obs} 18186(I > 2σ(I)), N_{var} 980, residuals* $R1(F)$ 0.0729, $wR2(F^2)$ 0.2059, GoF(all) 0.928, $\Delta\rho_{min,max}$ -0.538, 0.567 e$^-$ Å^{-3}.

*$R1 = \Sigma||F_o| - |F_c||/\Sigma|F_o|$ for $F_o > 2\sigma(F_o)$; $wR2 = (\Sigma w(F_o^2 - F_c^2)^2/\Sigma(wF_c^2)^2)^{1/2}$ all reflections

$w=1/[\sigma^2(F_o^2)+(0.1415P)^2]$ where $P=(F_o^2+2F_c^2)/3$

Specific refinement details:

Rapid handling prior to flash cooling in liquid nitrogen and the use of synchrotron radiation enabled collection of high-resolution data to around 0.7 Å resolution. The asymmetric unit was found to contain one quarter of a $Fe_4L^{Me}{}_4$ assembly (i.e. one metal ion and one complete ligand) and associated counterions and solvent molecules. The structure was refined in the chiral space group I 2 2 2 with the Flack parameter refining to 0.090(5). We assume the bulk sample is a racemic mixture of crystals of the two enantiomers.

The two expected counterions were disordered over five lattice sites, one of which was located on a special position. Substantial bond length and thermal parameter restraints were applied to facilitate stable refinement of the anions and the fluorine atoms of the low occupancy anions were modelled with isotropic thermal parameters. The occupancy of the located hexafluorophosphate anions were allowed to freely refine which resulted in a discrepancy of 2.4 anions per $Fe_4L^{Me}{}_4$ assembly (or 0.6 per asymmetric unit). All benzene solvent molecules were modelled as rigid groups (AFIX 66) and two were refined as disordered over two or three positions.

The remaining anions (included as hexafluorophosphate in the formula) and further solvent within the lattice were significantly disordered and despite numerous attempts at modelling, including with rigid bodies no satisfactory model for the electron-density associated with them could be found. Consequently the SQUEEZE[14] function of PLATON[15] was employed to remove the contribution of the electron density associated with these remaining anions and further highly disordered solvent, which gave a potential solvent accessible void of 2064 Å^3 per unit cell (a total of approximately 9973 electrons). The diffuse solvent molecules could not be assigned to acetonitrile or benzene and were not included in the formula. Consequently, the molecular weight and density given above are underestimated.

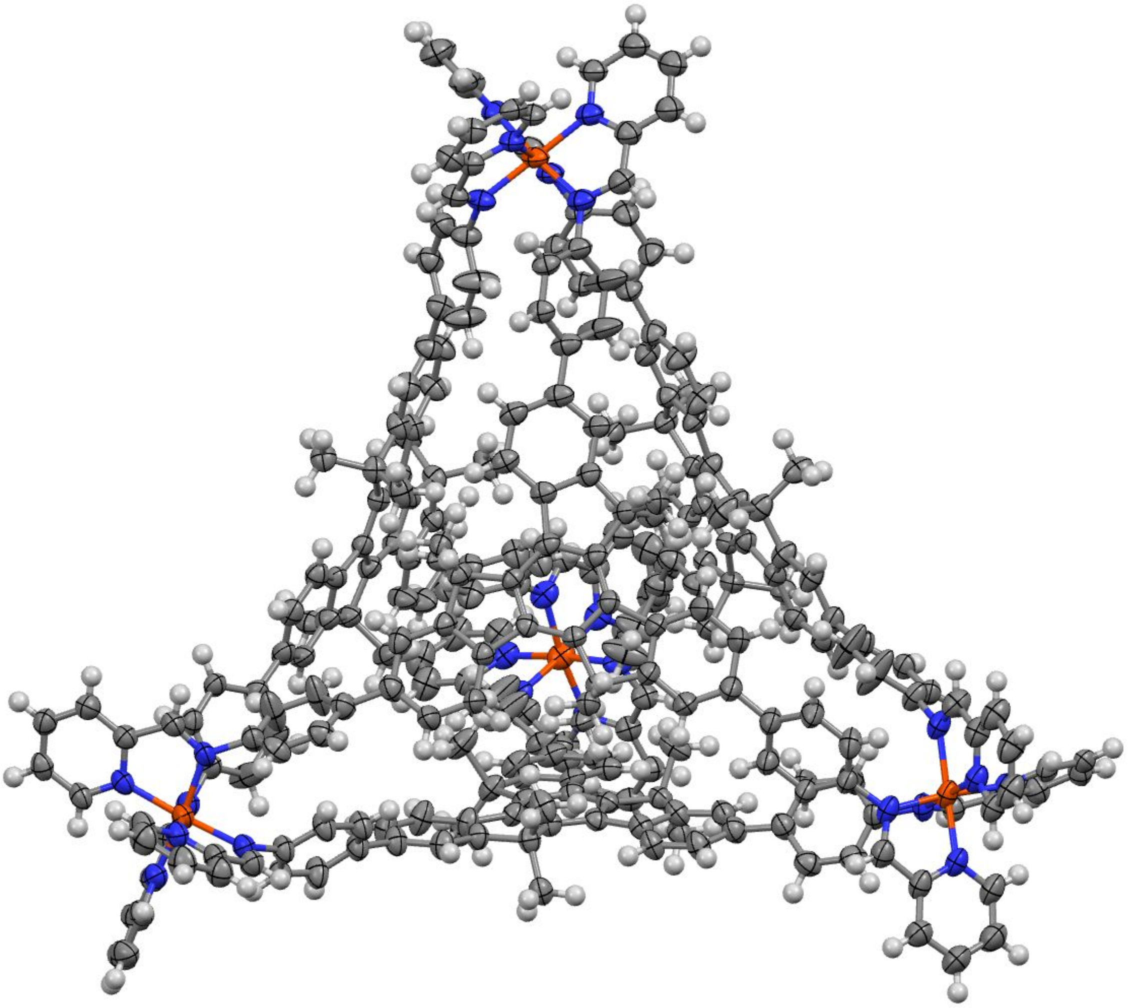

Figure S48. Cationic part of the crystal structure of **[$Fe_4L^{Me}{}_4$]·$8PF_6$·$9C_6H_6$·$3CH_3CN$**, with thermal ellipsoids at 50%.

$Fe_4L^{Et}{}_4$

$[Fe_4L^{Et}{}_4]\cdot 8BPh_4\cdot 5C_6H_6\cdot 9.75CH_3CN$ [+ solvent]

The crystals of $[Fe_4L^{Et}{}_4]\cdot 8BPh_4\cdot 5C_6H_6\cdot 9.75CH_3CN$ [+ solvent] were grown by diffusion of benzene into an acetonitrile solution of $[Fe_4L^{Et}{}_4]\cdot 8NTf_2$ containing excess $NaBPh_4$.

Formula $C_{541.50}H_{483.25}B_8Fe_4N_{33.75}$, *M* 7773.22, Triclinic, space group P -1 (#2), *a* 26.6122(3), *b* 27.1740(3), *c* 36.6968(5) Å, α 71.4350(12), β 71.0000(12), γ 66.7340(10)°, *V* 22494.5(6) Å^3, D_c 1.148 g cm^{-3}, *Z* 2, crystal size 0.030 by 0.020 by 0.015 mm, colour purple, habit block, temperature 100(2) Kelvin, λ(Synchrotron) 0.6889 Å, μ(Synchrotron) 0.173 mm^{-1}, T(Analytical)$_{min,max}$ 0.8851533337960166, 1.0, $2\theta_{max}$ 42.52, *hkl* range -28 28, -28 28, -38 38, *N* 179377, N_{ind} 54619(R_{merge} 0.0758), N_{obs} 21316(I > 2σ(I)), N_{var} 5125, residuals* $R1(F)$ 0.0988, $wR2(F^2)$ 0.2783, GoF(all) 0.874, $\Delta\rho_{min,max}$ -0.579, 0.711 e$^-$ Å^{-3}.

*$R1 = \Sigma||F_o| - |F_c||/\Sigma|F_o|$ for $F_o > 2\sigma(F_o)$; $wR2 = (\Sigma w(F_o^2 - F_c^2)^2/\Sigma(wF_c^2)^2)^{1/2}$ all reflections

$w=1/[\sigma^2(F_o^2)+(0.1635P)^2]$ where $P=(F_o^2+2F_c^2)/3$

Specific refinement details:

The crystals employed immediately lost solvent after removal from the mother liquor and rapid handling prior to flash cooling in liquid nitrogen was required to collect data. Despite these measures and the use of synchrotron radiation few reflections at greater than 0.95 Å resolution were observed and the data were trimmed accordingly. Furthermore, there was a significant drop-off in diffraction intensity after around 1.2 Å resolution resulting in a low ratio of observed/unique reflections but some meaningful reflections were observed to 0.95 Å resolution hence the data were not trimmed further. The quality of the data is far more than sufficient to establish the connectivity of the structure. The asymmetric unit was found to contain one complete Fe_4L_4 assembly and associated counterions and solvent molecules. Due to the limited resolution bond lengths and angles within pairs of chemically identical organic ligands were restrained to be similar to each other (SAME). Thermal parameter restraints (SIMU, RIGU) were applied to all atoms except for iron.

Three of the tetraphenylborate anions were modelled as fully or partly disordered over two locations and some show evidence of further unresolved disorder. Disordered anions were modelled with isotropic thermal parameters. The solvent molecules also show evidence of substantial disorder with many modelled over multiple locations and/or with partial occupancy. All benzene solvent molecules were modelled as rigid groups (AFIX 66) and bond length restraints were applied to most of the acetonitrile solvent molecules. The hydrogen atoms of some disordered acetonitrile molecules could not be located in the electron density map and were therefore not included in the model.

The SQUEEZE[14] function of PLATON[15] was employed to remove the contribution of electron density associated with further highly disordered solvent, which gave a potential solvent accessible void of 711 Å^3 per unit cell (a total of approximately 126 electrons). Diffuse solvent molecules could not be assigned to acetonitrile or benzene and were therefore not included in the formula. Consequently, the molecular weight and density given above are slightly underestimated.

CheckCIF gives three A and two B level alerts. One A and one B level alert result from the limited resolution of the data (low sine(theta_max)/wavelength and low ratio of observed/unique reflections) as described above. The other alerts all result from short contacts involving hydrogens of the ethyl substituents. One contact is with a tetraphenylborate anion which might be further disordered and two contacts are with protons of the truxene core. These contacts may be a genuine feature of the structure or due to unresolved disorder of the flexible ethyl groups resulting in their hydrogen atoms not being very precisely located.

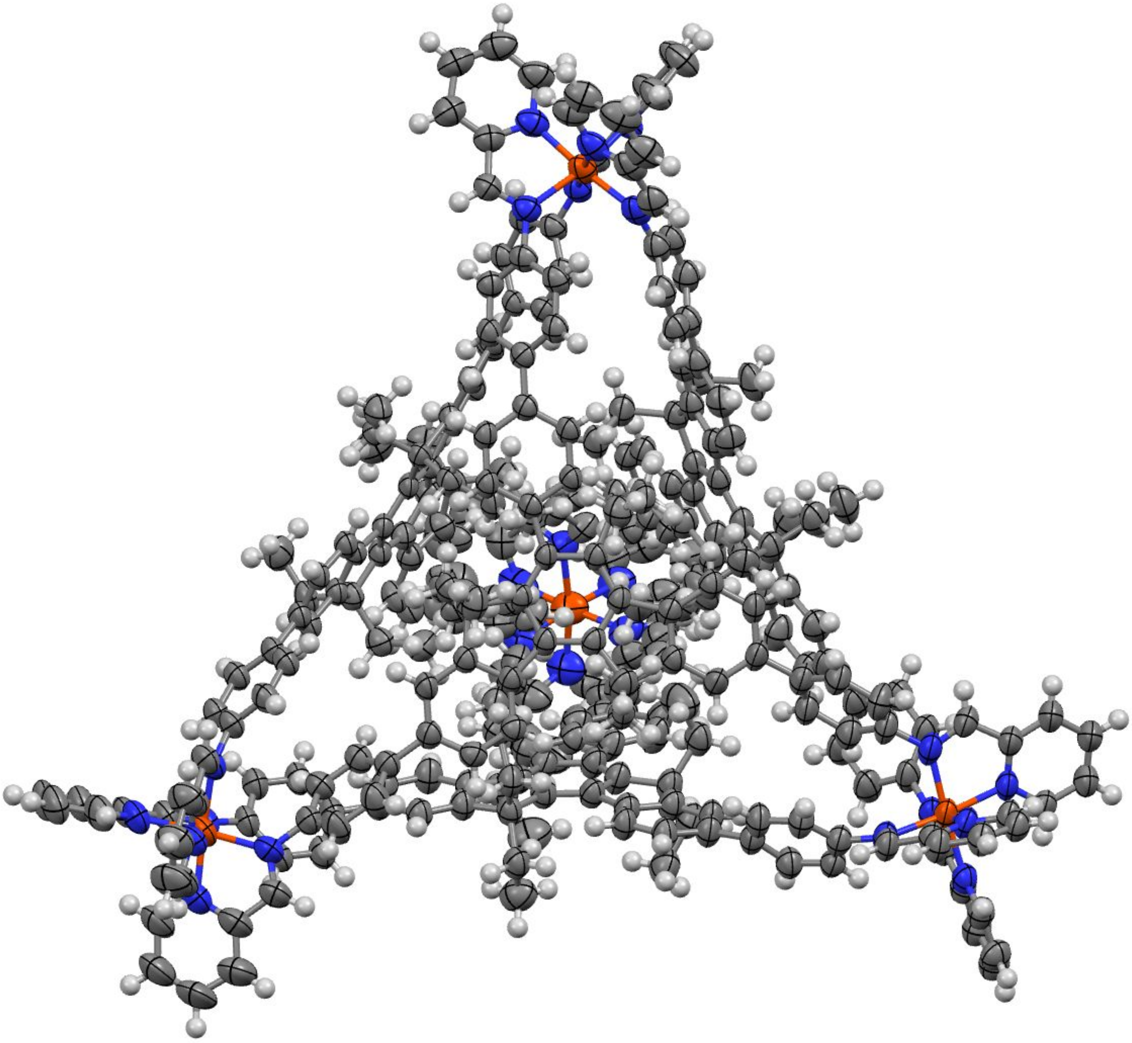

Figure S49. Cationic part of the crystal structure of **$[Fe_4L^{Et}{}_4]\cdot 8BPh_4\cdot 5C_6H_6\cdot 9.75CH_3CN$**, with thermal ellipsoids at 50%.

$Zn_4L^{Me}{}_4$

$[Zn_4L^{Me}{}_4]\cdot 8AsF_6$ [+ solvent]

The crystals of $[Zn_4L^{Me}{}_4]\cdot 8AsF_6$ [+ solvent] were grown by diffusion of diethyl ether into an acetonitrile solution of $[Zn_4L^{Me}{}_4]\cdot 8NTf_2$ containing excess $KAsF_6$.

Formula $C_{276}H_{216}As_8F_{48}N_{24}Zn_4$, *M* 5641.56, Monoclinic, space group C 2 (#5), *a* 37.8099(5), *b* 32.5394(3), *c* 26.3489(4) Å, β 133.6370(10), *V* 23461.3(6) Å^3, D_c 0.799 g cm^{-3}, *Z* 2, crystal size 0.080 by 0.060 by 0.060 mm, colour yellow, habit prism, temperature 100(2) Kelvin, λ(Synchrotron) 0.6889 Å, μ(Synchrotron) 0.747 mm^{-1}, T(Analytical)$_{min,max}$ 0.7484381670851683, 1.0, $2\theta_{max}$ 40.30, *hkl* range -37 37, -32 32, -26 26, *N* 39764, N_{ind} 23093(R_{merge} 0.0324), N_{obs} 14983(I > 2σ(I)), N_{var} 1439, residuals* $R1(F)$ 0.0544, $wR2(F^2)$ 0.1565, GoF(all) 0.906, $\Delta\rho_{min,max}$ -0.243, 0.228 e$^-$ Å^{-3}.

*$R1 = \Sigma||F_o| - |F_c||/\Sigma|F_o|$ for $F_o > 2\sigma(F_o)$; $wR2 = (\Sigma w(F_o^2 - F_c^2)^2/\Sigma(wF_c^2)^2)^{1/2}$ all reflections
$w=1/[\sigma^2(F_o^2)+(0.1057P)^2]$ where $P=(F_o^2+2F_c^2)/3$

Specific refinement details:

The crystals employed immediately lost solvent after removal from the mother liquor and rapid handling prior to flash cooling in liquid nitrogen was required to collect data. Despite these measures and the use of synchrotron radiation few reflections at greater than 1 Å resolution were observed and the data were trimmed accordingly. The quality of the data is far more than sufficient to establish the connectivity of the structure. The asymmetric unit was found to contain one half of a Zn_4L_4 assembly and associated counterions. The structure was refined as a racemic twin in space group C2 with the Flack parameter refining to 0.475(13).

Due to the limited resolution bond lengths and angles within the two chemically identical organic ligands were restrained to be similar to each other (SAME). Some additional DFIX and DANG restraints were applied to parts of the structure displaying a higher degree of thermal motion. Thermal parameter restraints (SIMU, RIGU) were applied to all atoms except for zinc. One phenyl ring was modelled as disordered over two orientations.

The hexafloroarsenate anions show evidence of unresolved disorder. Substantial bond length and thermal parameter restraints were applied to facilitate stable refinement of the anions. The occupancy of the located hexafloroarsenate anions were allowed to freely refine which resulted in a discrepancy of 5.6 anions per $Zn_4L^{Me}{}_4$ assembly (or 2.8 per asymmetric unit). The fluorine atoms of the partial occupancy anions were modelled with isotropic thermal parameters. Attempts to resolve further minor occupancy positions of the anions were unsuccessful due to the limited resolution of the data.

The remaining anions (included as hexafloroarsenate in the formula) and solvent within the lattice were significantly disordered and despite numerous attempts at modelling, including with rigid bodies no satisfactory model for the electron-density associated with them could be found. Consequently the SQUEEZE[14] function of PLATON[15] was employed to remove the contribution of the electron density associated with these remaining anions and further highly disordered solvent, which gave a potential solvent accessible void of 13871 Å^3 per unit cell (a total of approximately 3425 electrons). The diffuse solvent molecules could not be assigned to acetonitrile or diethyl ether and were not included in the formula. Consequently, the molecular weight and density given above are underestimated.

CheckCIF gives an A level alert resulting from the limited resolution of the data and a B level alert for low bond precision due to both the limited resolution of the data and the relatively high thermal motion in parts of the structure.

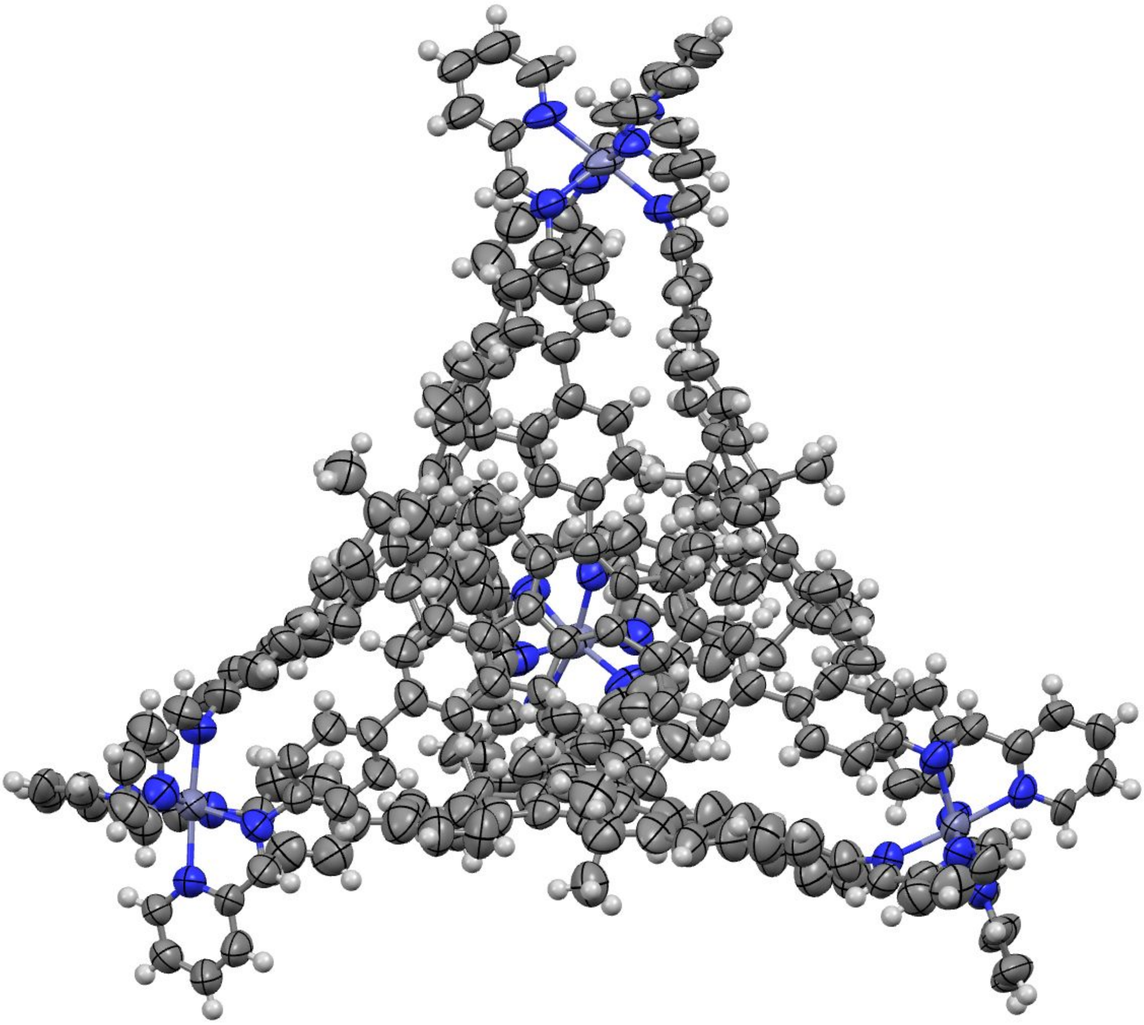

Figure S50. Cationic part of the crystal structure of **[Zn$_4$L^{Me}$_4$]·8AsF$_6$**, with thermal ellipsoids at 50%.

6. Distance, angle and volume comparisons

The following measurements were obtained from the crystal structures of the four $\mathbf{M_4L^R_4}$ cages.

Table S3. Comparison between distances in the crystal structures of the four cages.

**Cage** (CCDC number)	$\mathbf{Fe_4L^{Me}_4}$ (2497194)	$\mathbf{Zn_4L^{Me}_4}$ (2497195)	$\mathbf{Fe_4L^{Et}_4}$ (2497193)	$\mathbf{Zn_4L^{Et}_4}$ (2060406)
Average M-M distance (Å)	21.66	21.75	21.29	21.45
Average M-N bond length (Å)	1.98	2.17	1.99	2.19
Average truxene centroid-M centroid distance (Å)	5.94	5.85	5.44	5.36

Several trends can be observed:

- For each R group, the M-M distances are shorter in the Fe cage than the Zn cage, in line with shorter M-N bonds to Fe^{II}.
- In contrast, for each R group, the framework of the Zn cage is more compact than the Fe cage, reflected in shorter distances between the centres of the panels (defined as the centroid of the central truxene benzene ring) and the centre of the cage (defined as the centroid between the four metal centres).
- For each metal, the Et cage is more compact than the Me cage, as reflected in both shorter M-M distances and truxene centroid-metal centroid distances.

Next, the alkyl-alkyl contacts in the four structures were compared:

Table S4. Comparison between Me-Me contacts in the cavities of the four cages.

**Cage** (CCDC number)	$\mathbf{Fe_4L^{Me}_4}$ (2497194)	$\mathbf{Zn_4L^{Me}_4}$ (2497195)	$\mathbf{Fe_4L^{Et}_4}$ (2497193)	$\mathbf{Zn_4L^{Et}_4}$ (2060406)
Average Me-Me C-C distance (Å)	3.69	3.53	3.94	3.87
Average R-R H-H distance (Å)	3.18 (from H-H contacts <3.5 Å)	3.05 (from H-H contacts <3.5 Å)	2.92 (from H-H contacts <3.2 Å)	2.79 (from H-H contacts <3.2 Å)
Number of close (<3.2 Å) H-H contacts	16	34	61	58

In the Me cages, Me-Me contacts at every edge were considered. In the Et cages, both the methyl and methylene hydrogens form close contacts (<3.2 Å), 61 in $\mathbf{Fe_4L^{Et}_4}$ and 58 in $\mathbf{Zn_4L^{Et}_4}$. For simplicity, only the C-C distances of methyl-methyl (not methylene) contacts were compared in Table S4. Alkyl-alkyl H-H contacts below 3.2 Å were used to calculate the average distances in the Et cages. Note that this is a shorter range than used in the Me cages (which includes Me-Me contacts at all edges), since the dense network of alkyl hydrogens in the Et cages gives rise to many long-distance H-H contacts.

Despite the shorter M-M distances in the Fe cages, for each R, the Zn cage features shorter C-C contacts than the Fe cage, in line with the more compact arrangement of the truxene panels. Furthermore, the Me-Me C-C distances in $\mathbf{Zn_4L^{Me}_4}$ are more uniform than those in $\mathbf{Fe_4L^{Me}_4}$, as reflected in the standard deviations of 0.055 Å and 0.27 Å, respectively.

To gauge the distortion of the metal coordination sphere from optimal octahedral geometry, the deviation of the ∠N-M-N angles of all cis linkages from 90° was calculated (Table S5).

Table S5. Average deviation of the N-M-N angles from 90°, as a measure for strain around the coordination sphere.

**Cage** (CCDC number)	**Fe**$_4$**L**$^{\mathbf{Me}}$$_4$ (2497194)	**Zn**$_4$**L**$^{\mathbf{Me}}$$_4$ (2497195)	**Fe**$_4$**L**$^{\mathbf{Et}}$$_4$ (2497193)	**Zn**$_4$**L**$^{\mathbf{Et}}$$_4$ (2060406)	**Fe**$_4$**L**$^{\mathbf{triazine}}$$_4$ (976921)	**Zn**$_4$**L**$^{\mathbf{triazine}}$$_4$ (1896144)
Average deviation of ∠N-M-N from 90°	5.29	7.18	5.27	7.19	5.59	8.32

For each metal, the average deviations for the two R groups are comparable. Furthermore, the deviations in the Fe cages are comparable to those in low-spin **Fe**$_4$**L**$^{\mathbf{triazine}}$$_4$[16], and those in the Zn cages are slightly smaller than those in **Zn**$_4$**L**$^{\mathbf{triazine}}$$_4$[17]. These measurements indicate that there is no substantial angle strain around the metal coordination sphere, compared to the known triazine cages, which selectively bind Fe^{II} over Zn^{II} (see Section 8).

Finally, the cavity volumes of the four cages were calculated using Molovol.[18] In addition, the cavities of MM3-minimized models of the disfavoured diastereomers were calculated.
Cavity volumes were calculated with a probe radius of 1.8 Å (single-probe mode).

Table S6. Calculated cavity volumes for the four cages and their diastereomers.

Cage	Cavity volume [Å^3]
**Fe$_4$L^{Me}$_4$ T_S (Δ_4/AC_4)** (MM3 model)	392
**Fe$_4$L^{Me}$_4$ T_L (Δ_4/C_4)** (crystal structure)	656
**Zn$_4$L^{Me}$_4$ T_S (Δ_4/AC_4)** (MM3 model)	377
**Zn$_4$L^{Me}$_4$ T_L (Δ_4/C_4)** (crystal structure)	659
**Fe$_4$L^{Et}$_4$ T_S (Δ_4/AC_4)** (crystal structure)	61
**Fe$_4$L^{Et}$_4$ T_L (Δ_4/C_4)** (MM3 model)	211
**Zn$_4$L^{Et}$_4$ T_S (Δ_4/AC_4)** (crystal structure)	54
**Zn$_4$L^{Et}$_4$ T_L (Δ_4/C_4)** (MM3 model)	210

For each R, the cavity of the $\boldsymbol{T_L}$ diastereomer is substantially larger than the $\boldsymbol{T_S}$ diastereomer. This results from the more compact $\boldsymbol{T_S}$ framework, and from the fact that the alkyl groups in $\boldsymbol{T_S}$ enclose a central cavity, whereas the alkyl groups in $\boldsymbol{T_L}$ are oriented in a way that allows the cavity to extend toward the vertices (Figure S51).

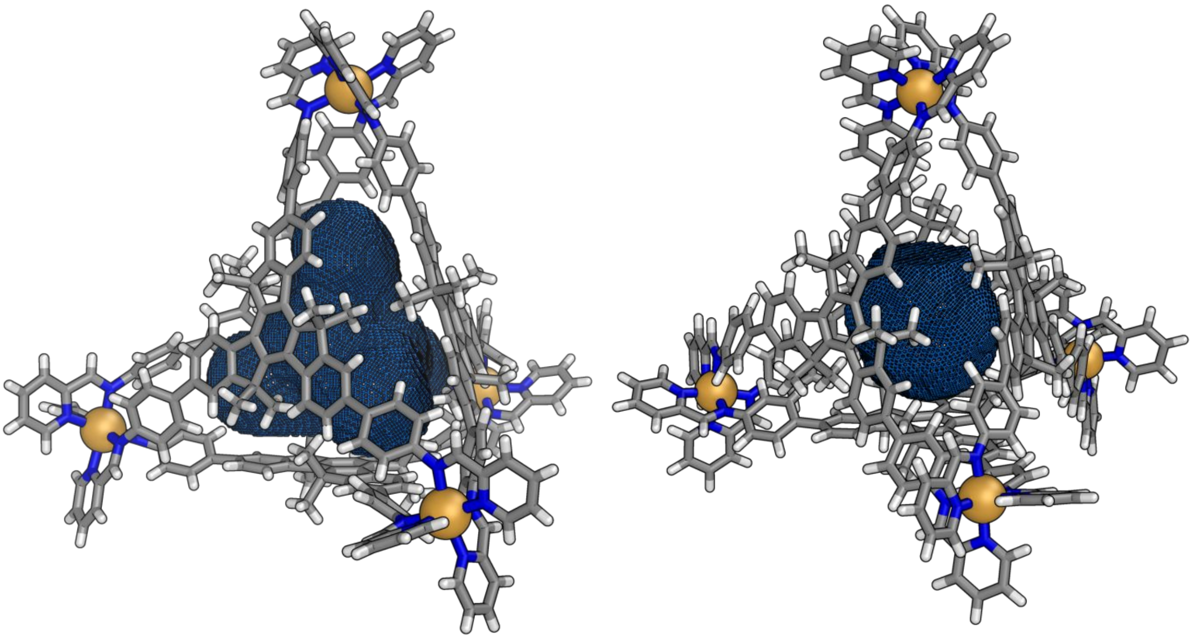

Figure S51. Left: the crystal structure of **Zn$_4$L^{Me}$_4$** ($\boldsymbol{T_L}$) with the cavity outlined in dark blue. Right: MM3-optimized structure of the $\boldsymbol{T_S}$ diastereomer of **Zn$_4$L^{Me}$_4$** with the cavity outlined in dark blue.

7. Computational studies

Quantum mechanical calculations were carried out on two models representing the $\boldsymbol{T}_{\mathrm{L}}$ ($\boldsymbol{\Delta}_4$,$\boldsymbol{C}_4$) and $\boldsymbol{T}_{\mathrm{S}}$ ($\boldsymbol{\Lambda}_4$,$\boldsymbol{C}_4$) diastereomers of a non-alkylated cage, $\mathbf{Zn_4L^H_4}$. The input structures were derived from the crystal structures of $\mathbf{Fe_4L^{Me}_4}$ and $\mathbf{Fe_4L^{Et}_4}$, with the Me and Et side groups replaced by hydrogens and the Fe^{II} ions substituted with Zn^{II}. Semi-empirical methods such as GFN2-xTB often struggle to accurately describe open-shell transition metals like the Fe^{II} centres in $\mathbf{Fe_4L^{Et}_4}$. We therefore only investigated the Zn-containing cages (which displayed analogous stereochemical behaviour to their Fe congeners). Counterions and explicit solvent were not included in the calculations.

Semi-empirical calculations were performed using the OPTIM program,[19] which contains a variety of geometry optimization tools for locating stationary points on potential energy surfaces and calculating reaction pathways. Geometry optimizations were performed on $\boldsymbol{T}_{\mathrm{L}}$-$\mathbf{Zn_4L^H_4}$ and $\boldsymbol{T}_{\mathrm{S}}$-$\mathbf{Zn_4L^H_4}$ using the OPTIM interface to the *xtb* program,[20] which was employed to calculate the energy and gradient at the GFN2-xTB[21-23] level of theory. For each calculation, the overall charge was 8+, the accuracy parameter was set to 0.01, and implicit acetonitrile (MeCN) solvation was included using the *alpb* method.[24] In both cases a well-converged structure was obtained.

The GFN2-xTB optimized structures were further optimized using Density Functional Theory (DFT) via the ORCA program.[25] Geometry optimizations were performed using the r^2SCAN-3c method[26] in the gas-phase, which includes D4 dispersion and geometrical counter-poise corrections for London dispersion. A tight self-consistent field (*TightSCF*) convergence criterion, the RI-J approximation (*def2/J*[27]), a charge of 8+, and a spin multiplicity of 1 were applied. Additionally, the *SlowConv* keyword was applied to circumvent convergence issues. The r^2SCAN-3c optimization of $\boldsymbol{T}_{\mathrm{S}}$-$\mathbf{Zn_4L^H_4}$ produced a geometry slightly different from the GFN2-xTB result. Re-optimizing this r^2SCAN-3c geometry at the GFN2-xTB level produced a structure equivalent to the r^2SCAN-3c geometry. The lower GFN2-xTB energy of this new structure indicates that it is more stable than the previous structure, suggesting that it provides a more reliable representation of the ground-state geometry of $\boldsymbol{T}_{\mathrm{S}}$-$\mathbf{Zn_4L^H_4}$. The r^2SCAN-3c optimized geometry of $\boldsymbol{T}_{\mathrm{L}}$-$\mathbf{Zn_4L^H_4}$ closely resembled that of the GFN2-xTB optimized geometry of $\boldsymbol{T}_{\mathrm{S}}$-$\mathbf{Zn_4L^H_4}$. Attempts to optimize the $Zn_4L^H_4$ structures at the r^2SCAN-3c level with implicit acetonitrile solvation did not lead to convergence. Therefore, single-point r^2SCAN-3c calculations with implicit acetonitrile solvation (with the *defgrid3* keyword) were performed on the previously obtained GFN2-xTB optimized structures to account for solvent effects (denoted as r^2SCAN-3c//GFN2-xTB). The relative GFN2-xTB (MeCN), r^2SCAN-3c//GFN2-xTB (MeCN) and r^2SCAN-3c (g) energies for $\boldsymbol{T}_{\mathrm{L}}$-$\mathbf{Zn_4L^H_4}$ and $\boldsymbol{T}_{\mathrm{S}}$-$\mathbf{Zn_4L^H_4}$ are summarized in Table S7 and the corresponding optimized geometries are shown in Figure S52.

Table S7. Comparison of the energies in kcal/mol for the two diastereomers $\boldsymbol{T}_{L}$-$\mathbf{Zn_4L^H_4}$ and $\boldsymbol{T}_{S}$-$\mathbf{Zn_4L^H_4}$. With all methods, $\boldsymbol{T}_{S}$-$\mathbf{Zn_4L^H_4}$ was found to be lower in energy.

**Method**	**Relative energy (kcal/mol)**	
	$\boldsymbol{T}_{\mathrm{L}}$-$\mathbf{Zn_4L^H_4}$	$\boldsymbol{T}_{\mathrm{S}}$-$\mathbf{Zn_4L^H_4}$
GFN2-xTB (MeCN)	0.00	-1.99
r^2SCAN-3c//GFN2-xTB (MeCN)	0.00	-14.24
r^2SCAN-3c (g)	0.00	-12.08

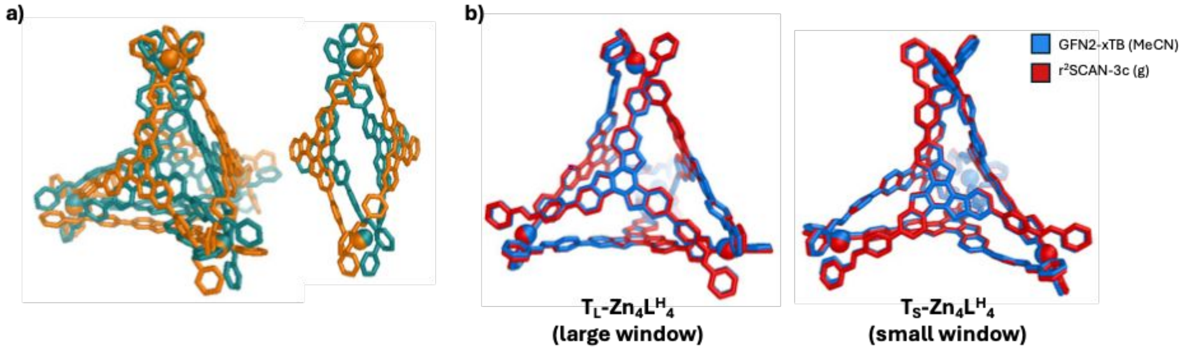

Figure S52. a. Comparison of the GFN2-xTB optimized structures of $\boldsymbol{T}_{L}$-$\mathbf{Zn_4L^H_4}$ (orange) and $\boldsymbol{T}_{S}$-$\mathbf{Zn_4L^H_4}$ (teal),

highlighting the difference in window apertures. b. Comparison of the geometry-optimized structures of ***T**$_L$***-Zn$_4$L^H$_4$** (left) and ***T**$_S$***-Zn$_4$L^H$_4$** (right) at the GFN2-xTB and r^{2}SCAN-3c levels of theory.

Vibrational analyses at the DFT level were unsuccessful for either diastereomer, preventing extra confirmation that true minima were achieved and precluding the calculation of Gibbs free energies. Nevertheless, the DFT-optimized structures are in good structural agreement with those found at the GFN2-xTB level of theory. We therefore consider the DFT geometries to be sufficiently reliable for our purpose of comparing relative stabilities. From the relative energies derived from several different methods, it can be concluded that the ***T**$_S$***-Zn$_4$L^H$_4$** diastereomer is significantly more stable than the ***T**$_L$***-Zn$_4$L^H$_4$** diastereomer. Similar behaviour is expected for the Fe^{II} analogue structures, which is supported by their comparable behaviour observed in experiments.

8. Competition experiments

L^{Me} vs. L^{Et} with limiting M^{II}

Competition with a mixture of subcomponents

To a J-Young NMR tube, $\mathbf{A^{Me}}$ (1 equiv., 2 mg, 2.9 µmol), $\mathbf{A^{Et}}$ (1 equiv., 2.2 mg, 2.9 µmol), $Fe(NTf_2)_2$ (92% purity, 1 equiv., 1.9 mg, 2.9 µmol) and 2-formylpyridine (3 equiv., 8.7 µmol, 10 µl of a stock solution containing 100 µl acetonitrile and 8.3 µl 2-formylpyridine) were added. Acetonitrile (500 µl) was added, and the tube was sealed and heated to 70 °C for 24 hours. A 20 µl aliquot was diluted with 200 µl of acetonitrile, filtered on glass fibre, and analysed by ESI-HRMS, which indicated the selective formation of $\mathbf{Fe_4L^{Et}_4}$ (Figure S54).

An analogous experiment was carried out using $Zn(NTf_2)_2$ (93% purity, 1 equiv., 1.9 mg, 2.9 µmol) in place of $Fe(NTf_2)_2$, resulting in the formation of $\mathbf{Zn_4L^{Et}_4}$ with minor amounts of $\mathbf{Zn_4L^{Me}_1L^{Et}_3}$ (Figure S58).

Subcomponent displacement from a pre-formed cage

To a J-young NMR tube, $\mathbf{Fe_4L^{Me}_4}$ (1 equiv., 2.26 mg, 0.36 µmol) and $\mathbf{A^{Et}}$ (4 equiv., 1.12 mg, 1.43 µmol) were added. Acetonitrile (500 µl) was added, and the tube was sealed and heated to 70 °C for 24 hours. A 20 µl aliquot was diluted with 200 µl of acetonitrile, filtered on glass fibre, and analysed by ESI-HRMS, which indicated the selective formation of $\mathbf{Fe_4L^{Et}_4}$ (Figure S55).

An analogous experiment starting from $\mathbf{Fe_4L^{Et}_4}$ (1 equiv., 2.38 mg, 0.36 µmol) and adding $\mathbf{A^{Me}}$ (4 equiv., 1 mg, 1.43 µmol) led to the same observed mass spectrum, namely complete selectivity for $\mathbf{Fe_4L^{Et}_4}$ (Figure S56).

The above experiments were repeated with the corresponding Zn-cages, resulting in the exclusive formation of $\mathbf{Zn_4L^{Et}_4}$ (Figures S59-S60).

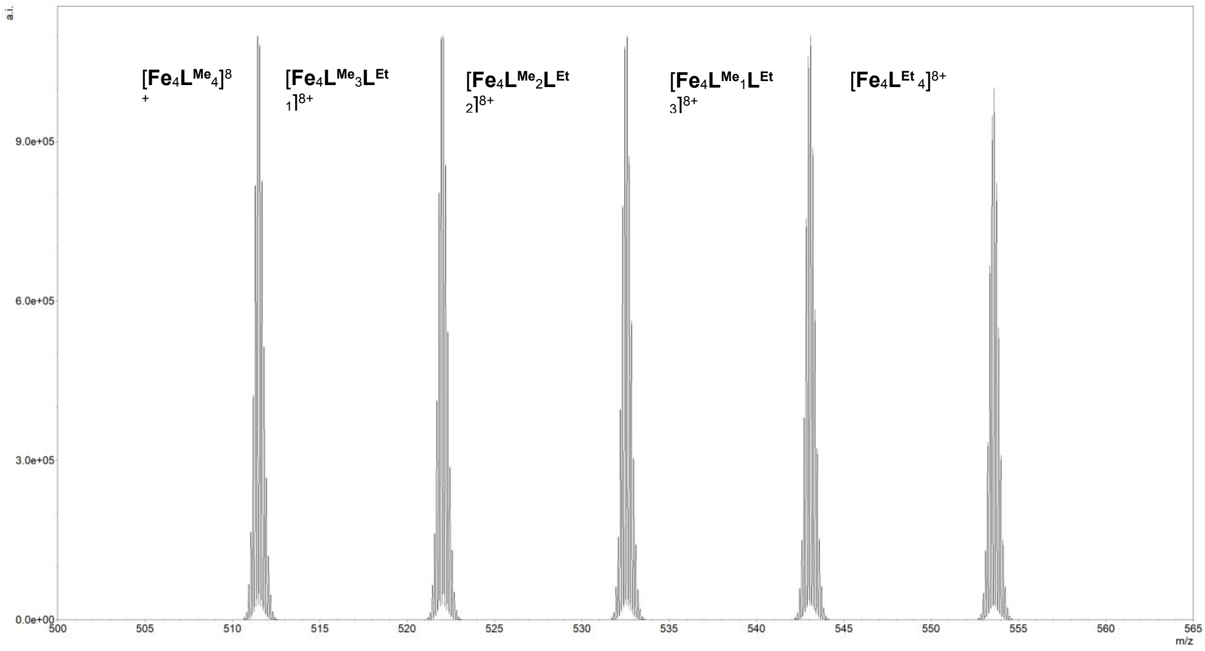

Figure S53. Predicted mass-spectrum peaks of the 8+ cations for the five possible cages resulting from mixing $\mathbf{Fe^{II}}$, $\mathbf{L^{Me}}$ and $\mathbf{L^{Et}}$. Left to right: $\mathbf{Fe_4L^{Me}_4}$, $\mathbf{Fe_4L^{Me}_3L^{Et}_1}$, $\mathbf{Fe_4L^{Me}_2L^{Et}_2}$, $\mathbf{Fe_4L^{Me}_1L^{Et}_3}$, $\mathbf{Fe_4L^{Et}_4}$.

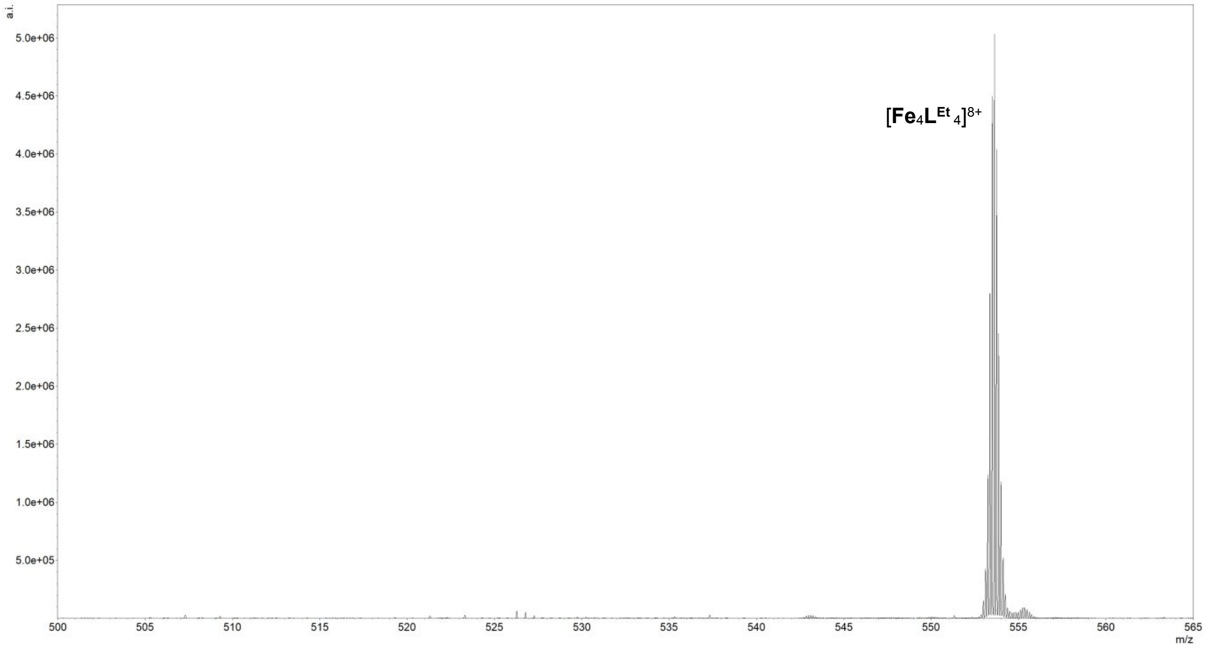

Figure S54. High-resolution mass-spectrum showing the exclusive formation of $\mathbf{Fe_4L^{Et}_4}$, starting from a 1/1 mixture of $\mathbf{A^{Me}}$ and $\mathbf{A^{Et}}$.

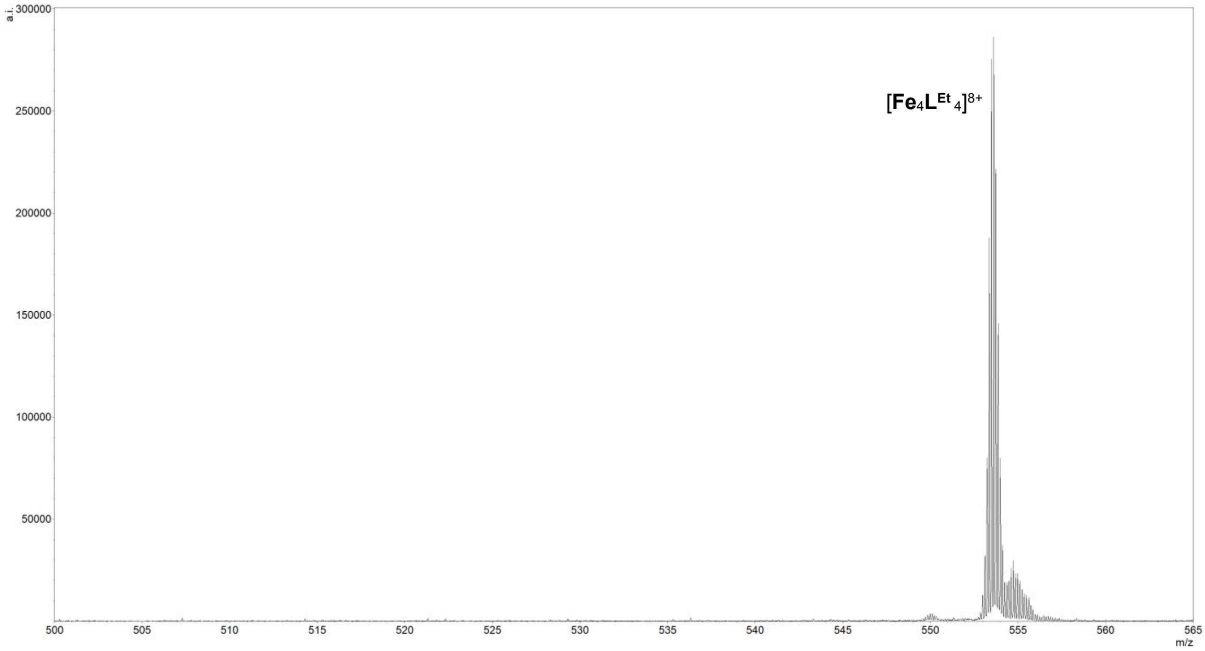

Figure S55. High-resolution mass-spectrum showing the preferential formation of **$Fe_4L^{Et}_4$** by subcomponent displacement from **$Fe_4L^{Me}_4$** upon addition of **A^{Et}**.

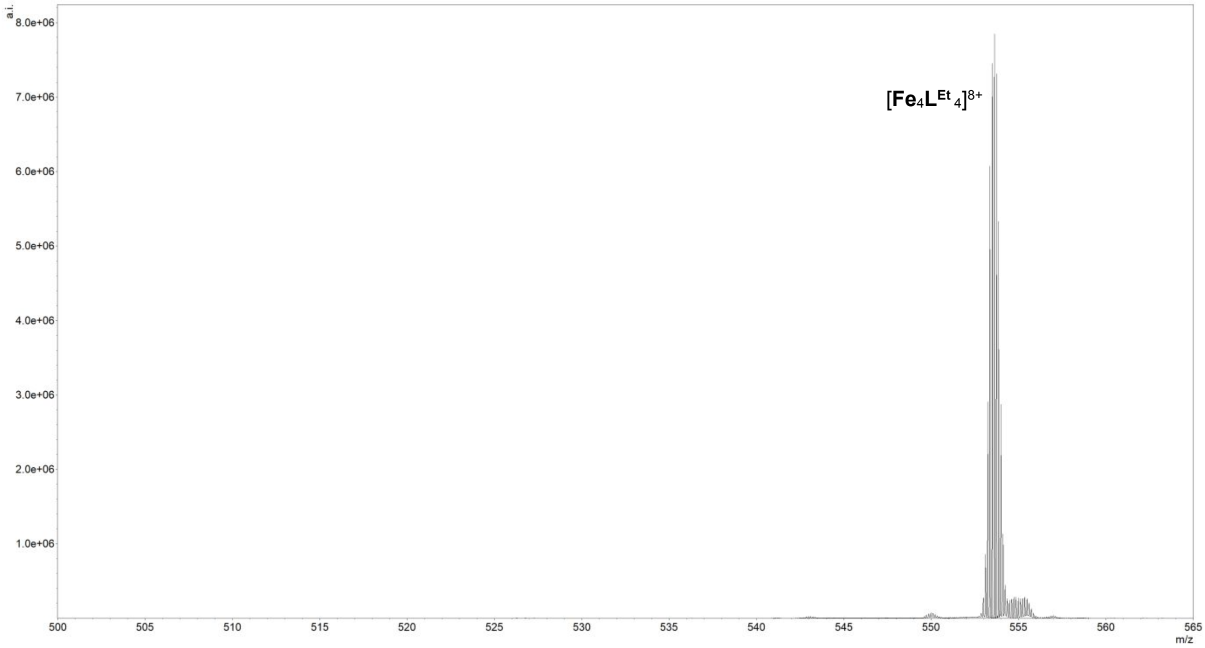

Figure S56. High-resolution mass-spectrum showing that no subcomponent displacement occurs upon addition of **A^{Me}** to **$Fe_4L^{Et}_4$**.

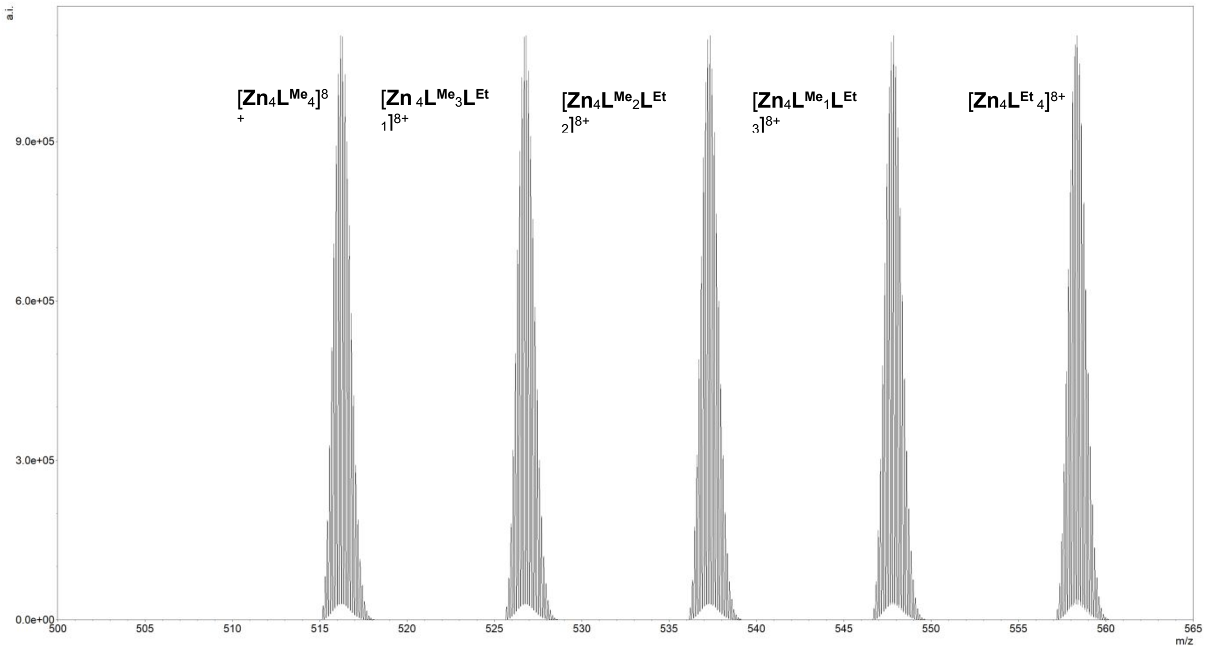

Figure S57. Predicted mass-spectrum peaks of the 8+ cations for the five possible cages resulting from mixing $\mathbf{Zn^{II}}$, $\mathbf{L^{Me}}$ and $\mathbf{L^{Et}}$. Left to right: $\mathbf{Zn_4L^{Me}}_4$, $\mathbf{Zn_4L^{Me}}_3\mathbf{L^{Et}}_1$, $\mathbf{Zn_4L^{Me}}_2\mathbf{L^{Et}}_2$, $\mathbf{Zn_4L^{Me}}_1\mathbf{L^{Et}}_3$, $\mathbf{Zn_4L^{Et}}_4$.

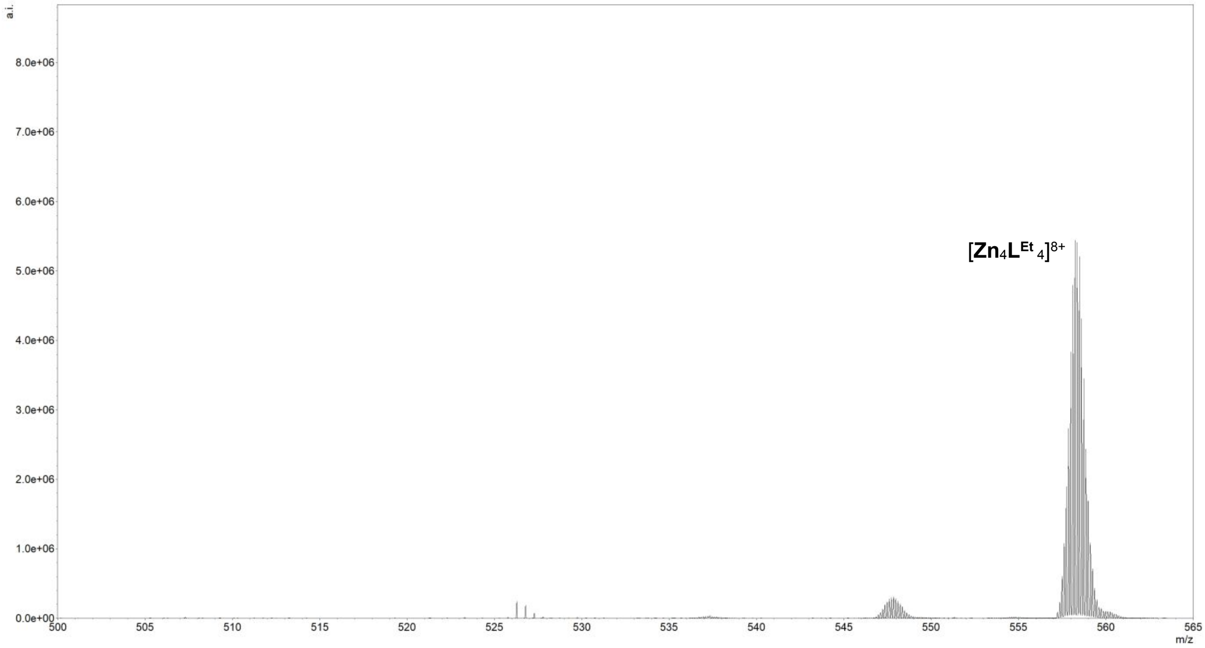

Figure S58. High-resolution mass-spectrum showing the preferential formation of $\mathbf{Zn_4L^{Et}}_4$, starting from a 1/1 mixture of $\mathbf{A^{Me}}$ and $\mathbf{A^{Et}}$.

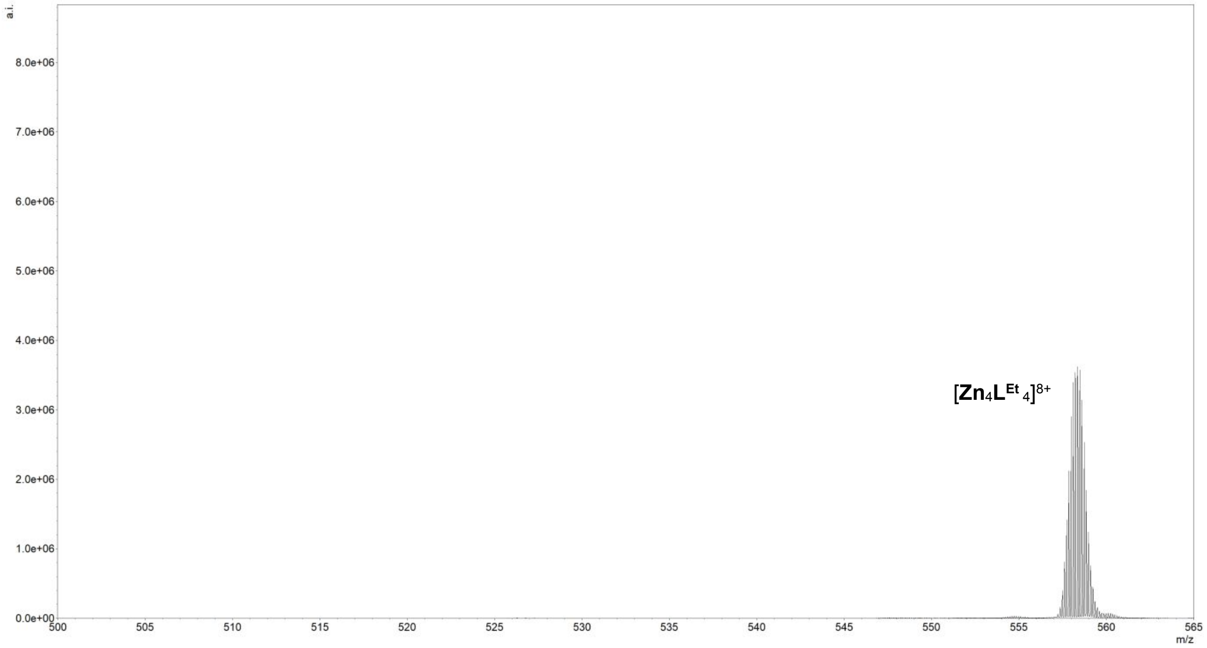

Figure S59. High-resolution mass-spectrum showing the preferential formation of $\mathbf{Zn_4L^{Et}{}_4}$ by subcomponent displacement from $\mathbf{Zn_4L^{Me}{}_4}$ upon addition of $\mathbf{A^{Et}}$.

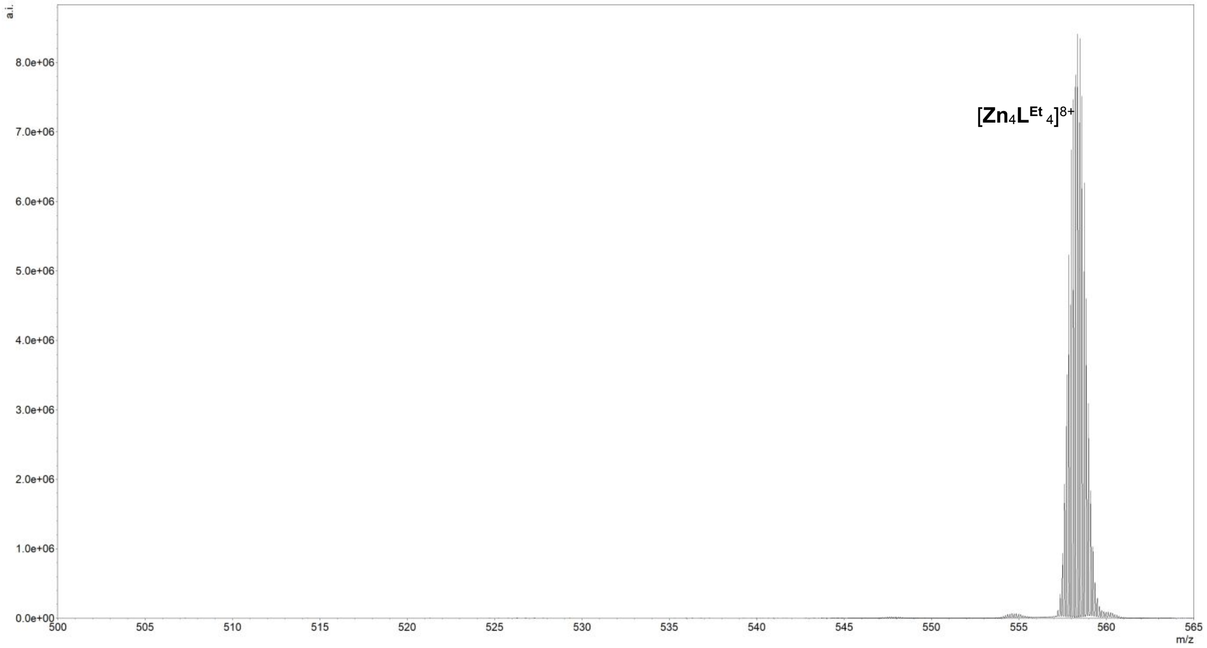

Figure S60. High-resolution mass-spectrum showing that no subcomponent displacement occurs upon addition of $\mathbf{A^{Me}}$ to $\mathbf{Zn_4L^{Et}{}_4}$.

Fe^{II} vs. Zn^{II} with limiting $\mathbf{A}^{\mathbf{triazine}}$

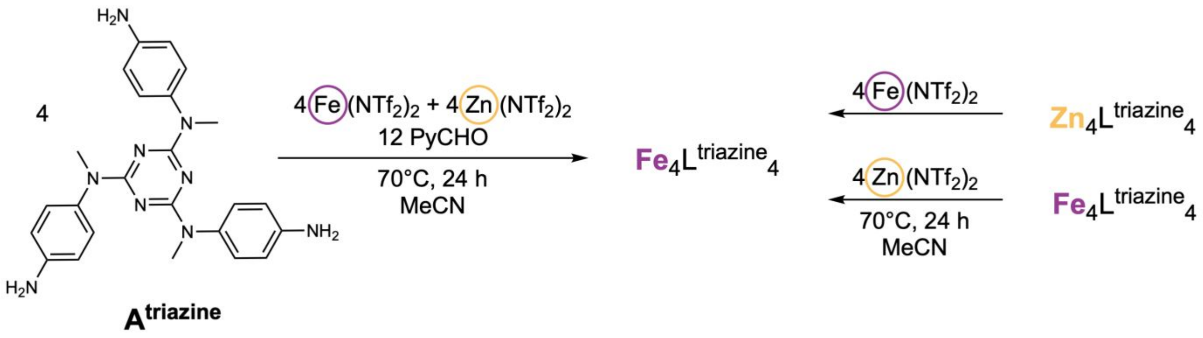

Figure S61. $\mathbf{Fe^{II}/Zn^{II}}$ competition with $\mathbf{A}^{\mathbf{triazine}}$, leading to preferential binding of $\mathbf{Fe^{II}}$.

Competition from subcomponents and both metal salts

To a J-Young NMR tube, $\mathbf{A}^{\mathbf{triazine}}$ (1 equiv., 2 mg, 4.5 µmol),[16] $Fe(NTf_2)_2$ (92% purity, 1 equiv., 3 mg, 4.5 µmol), $Zn(NTf_2)_2$ (93% purity, 1 equiv., 3 mg, 4.5 µmol), and 2-formylpyridine (3 equiv., 13.6 µmol, 1.45 µl) were added. Acetonitrile (500 µl) was added, and the solution was degassed through three freeze-pump-thaw cycles, flushed with nitrogen, sealed, and heated to 70 °C for 24 hours. A 20 µl aliquot was diluted with 200 µl of acetonitrile, filtered on glass fibre, and analysed by ESI-HRMS, which indicated the formation of a mixture of mixed metal cages featuring predominantly $\mathbf{Fe_4L^{triazine}}_4$, with minor amounts of $\mathbf{Fe_3Zn_1L^{triazine}}_4$ and traces of $\mathbf{Fe_2Zn_2}$ and $\mathbf{Fe_1Zn_3}$ (Figure S63).

M^{II} displacement from a pre-formed cage

To a J-Young NMR tube, $\mathbf{A}^{\mathbf{triazine}}$ (1 equiv., 2 mg, 4.5 µmol),[16] $Fe(NTf_2)_2$ (92% purity, 1 equiv., 3 mg, 4.5 µmol), and 2-formylpyridine (3 equiv., 13.6 µmol, 1.45 µl) were added. Acetonitrile (500 µl) was added, and the solution was degassed through three freeze-pump-thaw cycles, flushed with nitrogen, sealed, and heated to 70 °C for 24 hours. ESI-HRMS confirmed the formation of $\mathbf{Fe_4L^{triazine}}_4$.[16]

$Zn(NTf_2)_2$ (93% purity, 1 equiv., 3 mg, 4.5 µmol) was then added to the above mixture, and the mixture was heated to 70 °C for 24 hours. A 20 µl aliquot was diluted with 200 µl of acetonitrile, filtered on glass fibre, and analysed by ESI-HRMS, which an essentially identical spectrum to the one obtained when starting from $\mathbf{A}^{\mathbf{triazine}}$ and a mixture of both metals (Figure S64).

The analogous experiment starting from $\mathbf{Zn_4L^{triazine}}_4$[17] and adding $Fe(NTf_2)_2$ gave a similar result (Figure S65).

Overall, these results indicate a clear preference for binding Fe^{II} over Zn^{II} by the pyridyl-imine ligands at the cage vertices, in line with the stronger metal-ligand bonds and crystal-field stabilization energy of the former.[28]

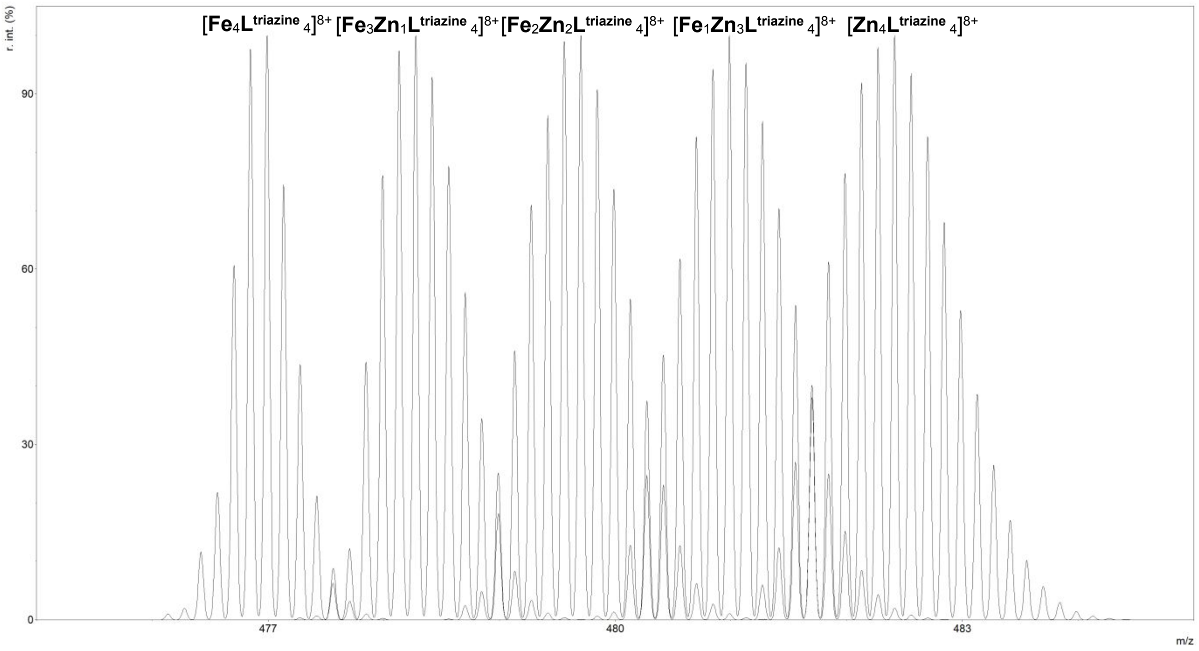

Figure S62. Predicted mass-spectrum peaks of the 8+ cations for the five possible cages resulting from mixing $\mathbf{Fe^{II}}$, $\mathbf{Zn^{II}}$, and $\mathbf{L^{triazine}}$. Left to right: $\mathbf{Fe_4L^{triazine}}_4$, $\mathbf{Fe_3Zn_1L^{triazine}}_4$, $\mathbf{Fe_2Zn_2L^{triazine}}_4$, $\mathbf{Fe_3Zn_1L^{triazine}}_4$, $\mathbf{Fe_4L^{triazine}}_4$.

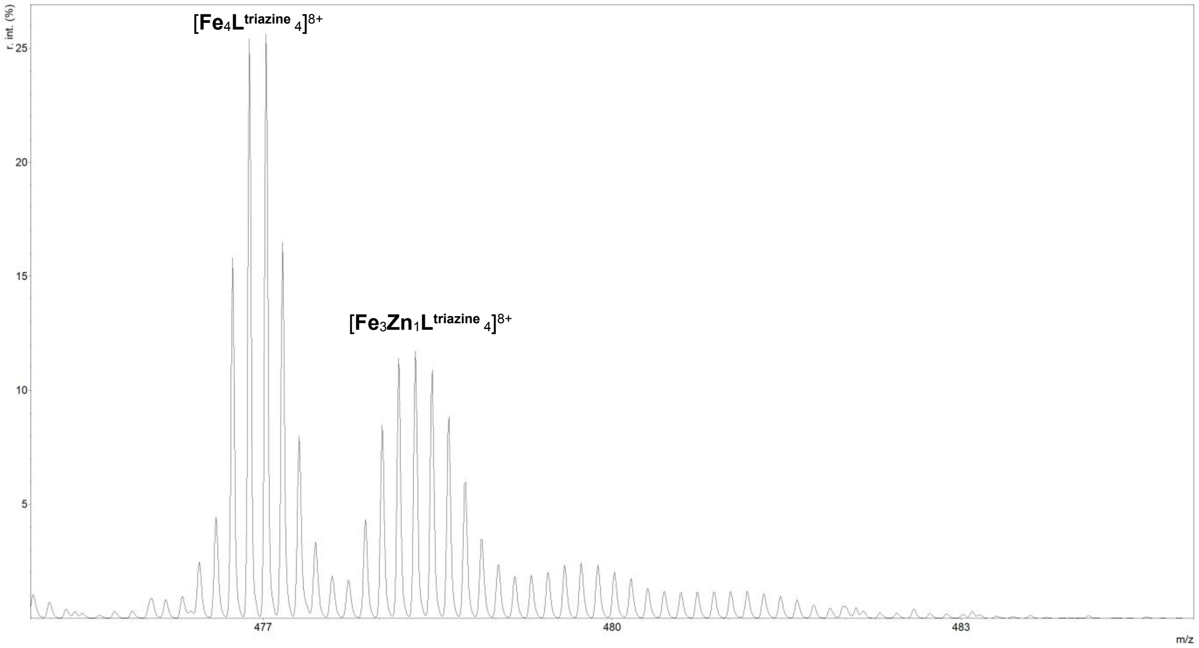

Figure S63. High-resolution mass-spectrum showing the preferential formation of $\mathbf{Fe_4L^{triazine}}_4$, starting from $\mathbf{A^{triazine}}$ and a mixture of $Fe(NTf_2)_2$ and $Zn(NTf_2)_2$.

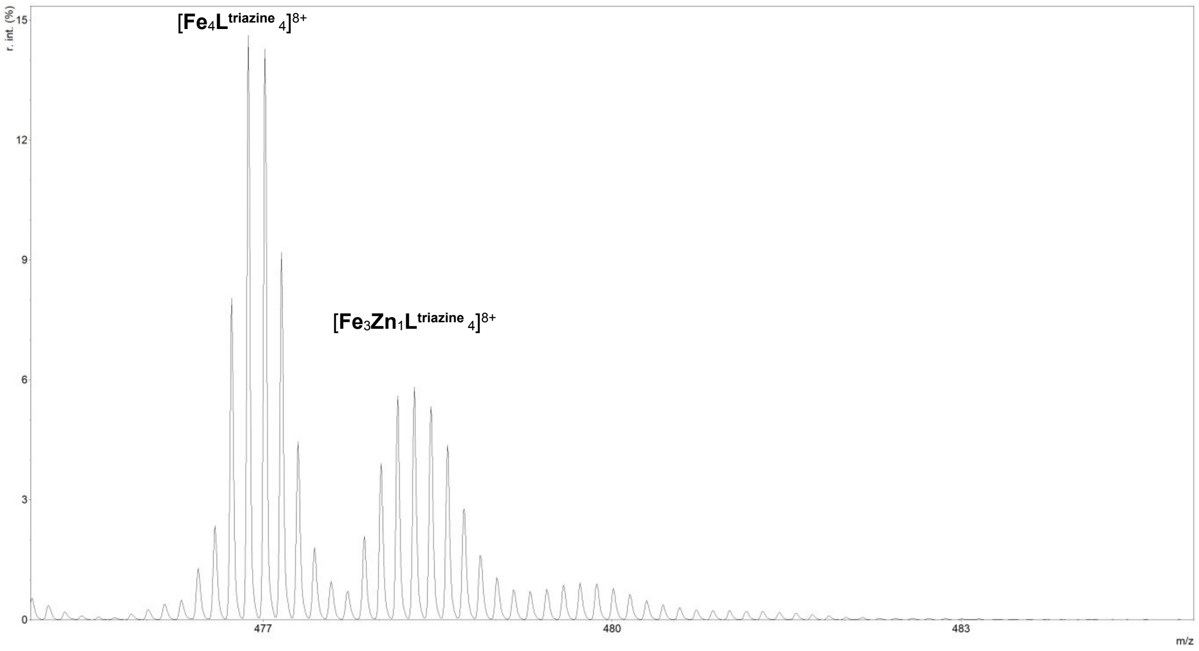

Figure S64. High-resolution mass-spectrum showing the preferential formation of **$Fe_4L^{triazine}_4$** with minor amounts of **$Fe_3Zn_1L^{triazine}_4$** upon addition of $Zn(NTf_2)_2$ to **$Fe_4L^{triazine}_4$**.

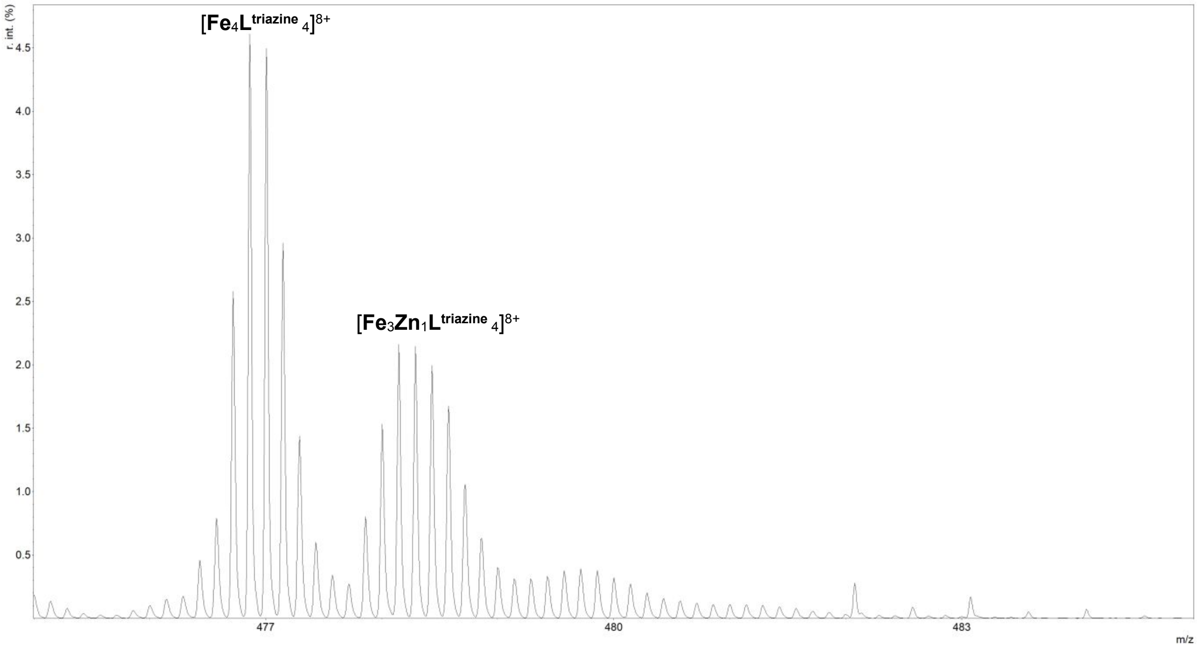

Figure S65. High-resolution mass-spectrum showing the preferential formation of **$Fe_4L^{triazine}_4$** with minor amounts of **$Fe_3Zn_1L^{triazine}_4$** upon addition of $Fe(NTf_2)_2$ to **$Zn_4L^{triazine}_4$**.

Fe^{II} vs. Zn^{II} with limiting A^R

From subcomponent and both metal salts

To a J-Young NMR tube, $\mathbf{A^{Me}}$ (1 equiv., 2 mg, 2.9 μmol), $Fe(NTf_2)_2$ (92% purity, 1 equiv., 1.9 mg, 2.9 μmol), $Zn(NTf_2)_2$ (93% purity, 1 equiv., 1.9 mg, 2.9 μmol), and 2-formylpyridine (3 equiv., 8.7 μmol, 10 μl of a stock solution containing 100 μl acetonitrile and 8.3 μl 2-formylpyridine) were added. Acetonitrile (500 μl) was added, and the solution was degassed through three freeze-pump-thaw cycles, flushed with nitrogen, sealed, and heated to 70 °C for 24 hours. A 20 μl aliquot was diluted with 200 μl of acetonitrile, filtered on glass fibre, and analysed by ESI-HRMS, which indicated the formation of a mixture of mixed metal cages featuring predominantly $\mathbf{Fe_2Zn_2L^{Me}_4}$ (Figure S67).

An analogous experiment with $\mathbf{A^{Et}}$ (1 equiv., 2.2 mg, 2.9 μmol) in place of $\mathbf{A^{Me}}$ led to a mixture of cages containing predominantly $\mathbf{Zn_4L^{Et}_4}$ and $\mathbf{Zn_3Fe_1L^{Et}_4}$ (Figure S71).

Metal displacement from a pre-formed cage

To a J-young NMR tube, $\mathbf{Fe_4L^{Me}_4}$ (1 equiv., 2.26 mg, 0.36 μmol) and $Zn(NTf_2)_2$ (93% purity, 1 equiv., 0.96 mg, 1.5 μmol) were added. Acetonitrile (500 μl) was added, and the solution was degassed through three freeze-pump-thaw cycles, flushed with nitrogen, sealed, and heated to 70 °C for 24 hours. A 20 μl aliquot was diluted with 200 μl of acetonitrile, filtered on glass fibre, and analysed by ESI-HRMS, which indicated the formation of a mixture of all four mixed metal cages featuring predominantly $\mathbf{Fe_2Zn_2L^{Me}_4}$ (Figure S68).

An analogous experiment was performed starting from $\mathbf{Zn_4L^{Me}_4}$ (1 equiv., 2.28 mg, 0.36 μmol) and $Fe(NTf_2)_2$, leading to a similar distribution of products (Figure S69).

Two analogous experiments were performed on the corresponding Et cages, which preferentially incorporated Zn^{II} (Figure S72-S73).

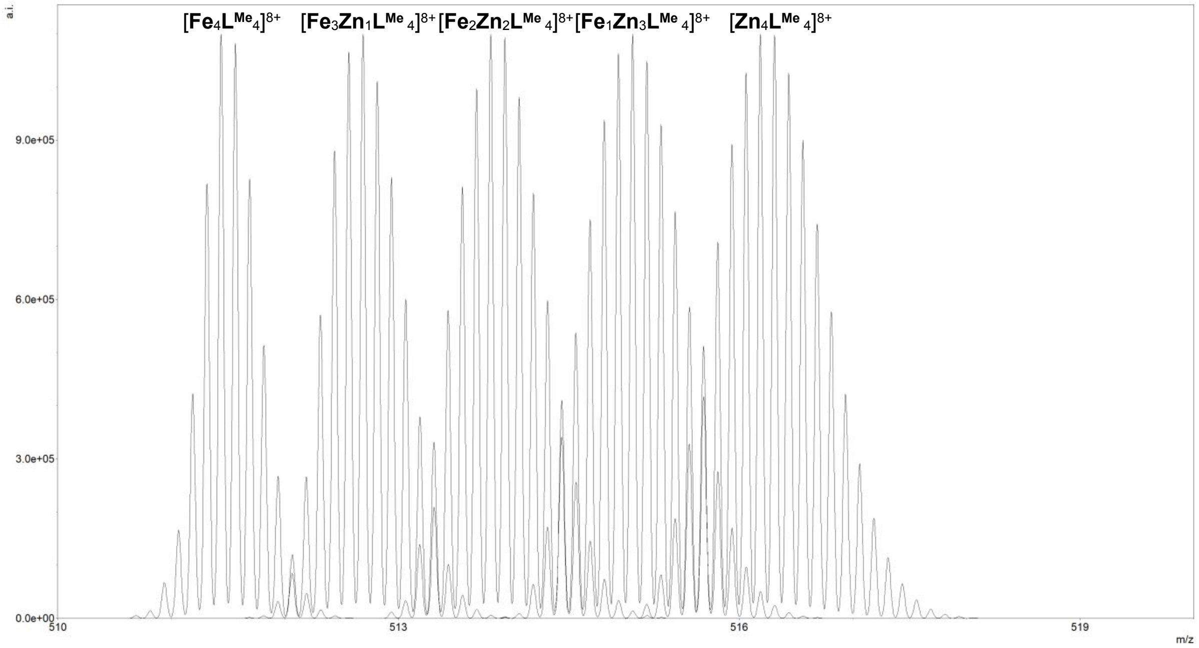

Figure S66. Predicted mass-spectrum peaks of the 8+ cations for the five possible cages resulting from mixing Fe^{II}, Zn^{II}, and $\mathbf{L^{Me}}$. Left to right: $\mathbf{Fe_4L^{Me}_4}$, $\mathbf{Fe_3Zn_1L^{Me}_4}$, $\mathbf{Fe_2Zn_2L^{Me}_4}$, $\mathbf{Fe_1Zn_3L^{Me}_4}$, $\mathbf{Zn_4L^{Me}_4}$.

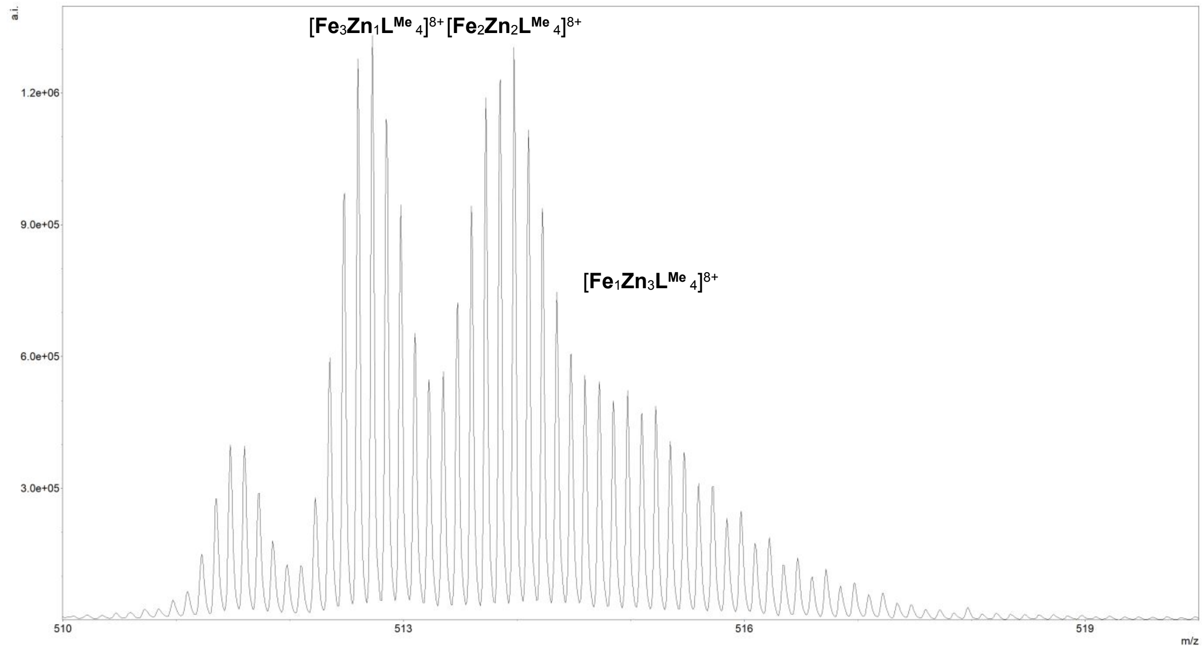

Figure S67. High-resolution mass-spectrum showing the preferential formation of $\mathbf{Fe_3Zn_1L^{Me}_4}$ and $\mathbf{Fe_2Zn_2L^{Me}_4}$, starting from $\mathbf{A^{Me}}$ and a mixture of $Fe(NTf_2)_2$ and $Zn(NTf_2)_2$.

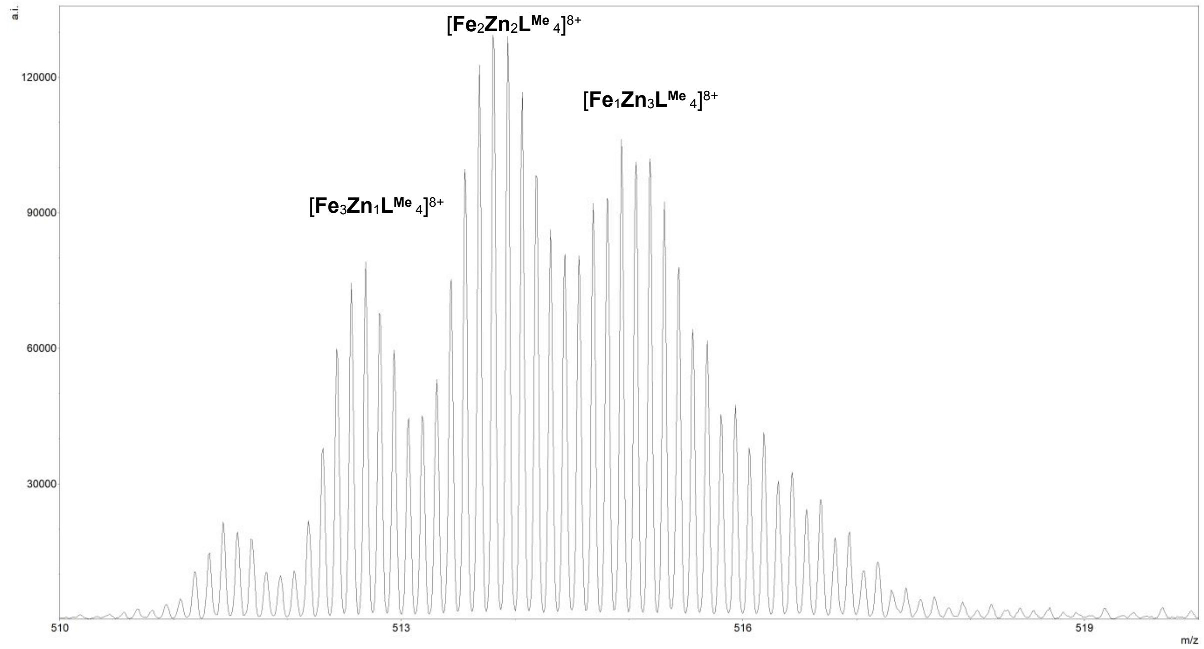

Figure S68. High-resolution mass-spectrum showing the preferential formation of $\mathbf{Fe_2Zn_2L^{Me}_4}$ upon addition of $Zn(NTf_2)_2$ to $\mathbf{Fe_4L^{Me}_4}$.

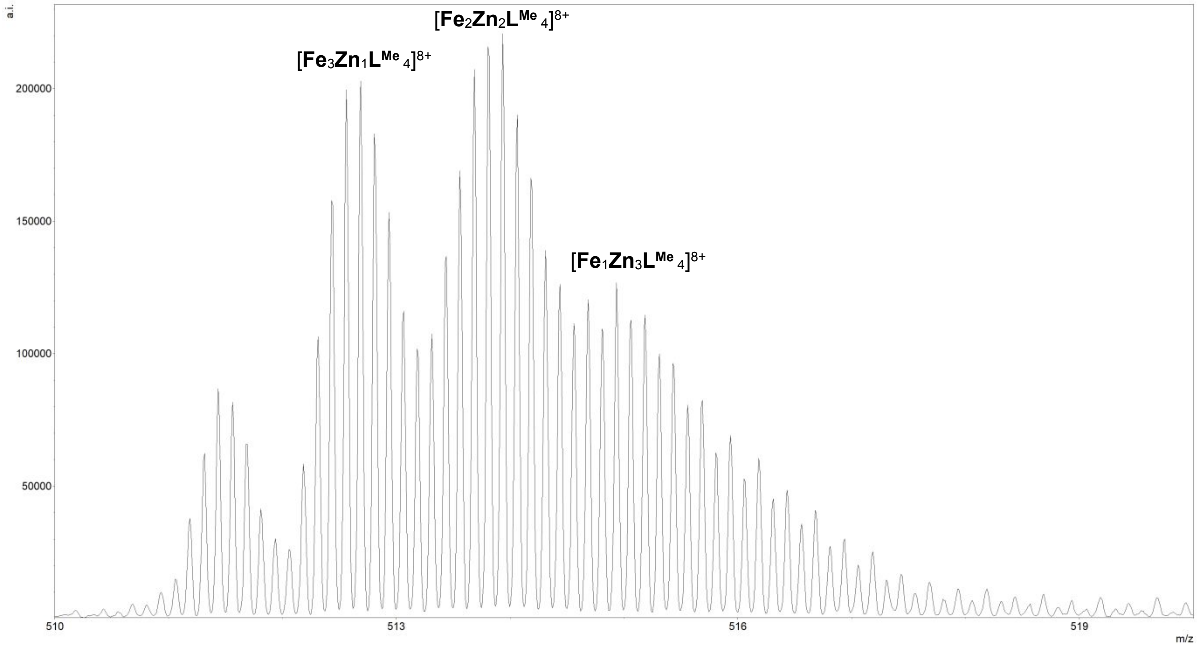

Figure S69. High-resolution mass-spectrum showing the preferential formation of **$Fe_2Zn_2L^{Me}_4$** upon addition of $Fe(NTf_2)_2$ to **$Zn_4L^{Me}_4$**.

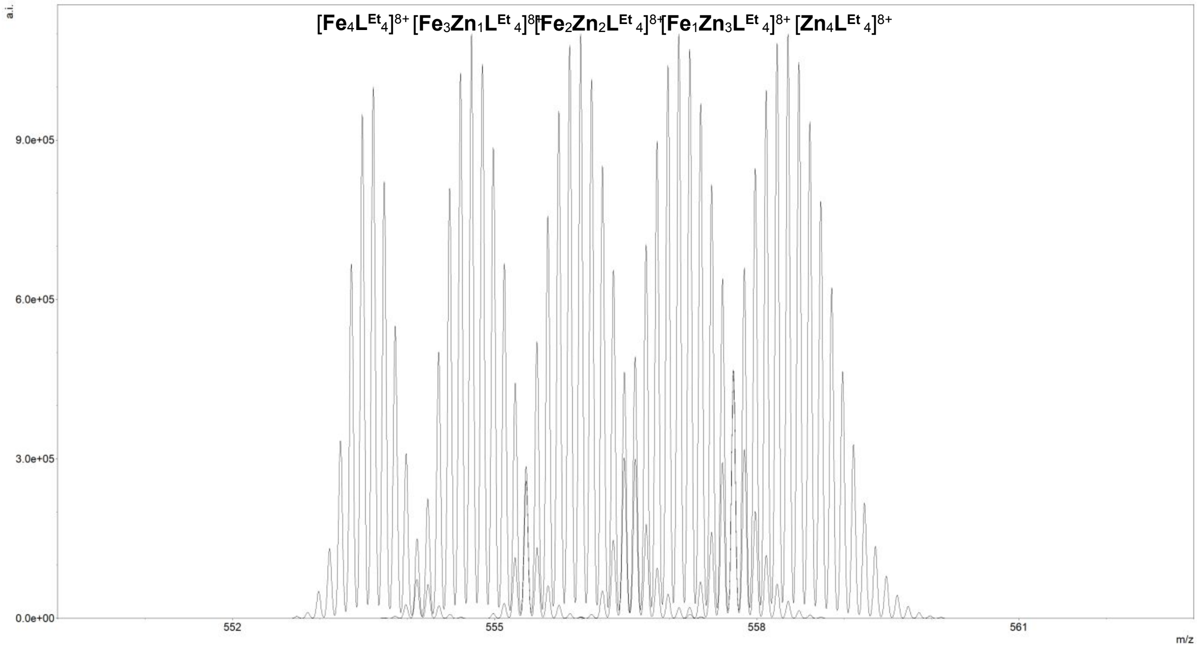

Figure S70. Predicted mass-spectrum peaks of the 8+ cations for the five possible cages resulting from mixing Fe^{II}, Zn^{II}, and **L^{Et}**. Left to right: **$Fe_4L^{Et}_4$**, **$Fe_3Zn_1L^{Et}_4$**, **$Fe_2Zn_2L^{Et}_4$**, **$Fe_1Zn_3L^{Et}_4$**, **$Zn_4L^{Et}_4$**.

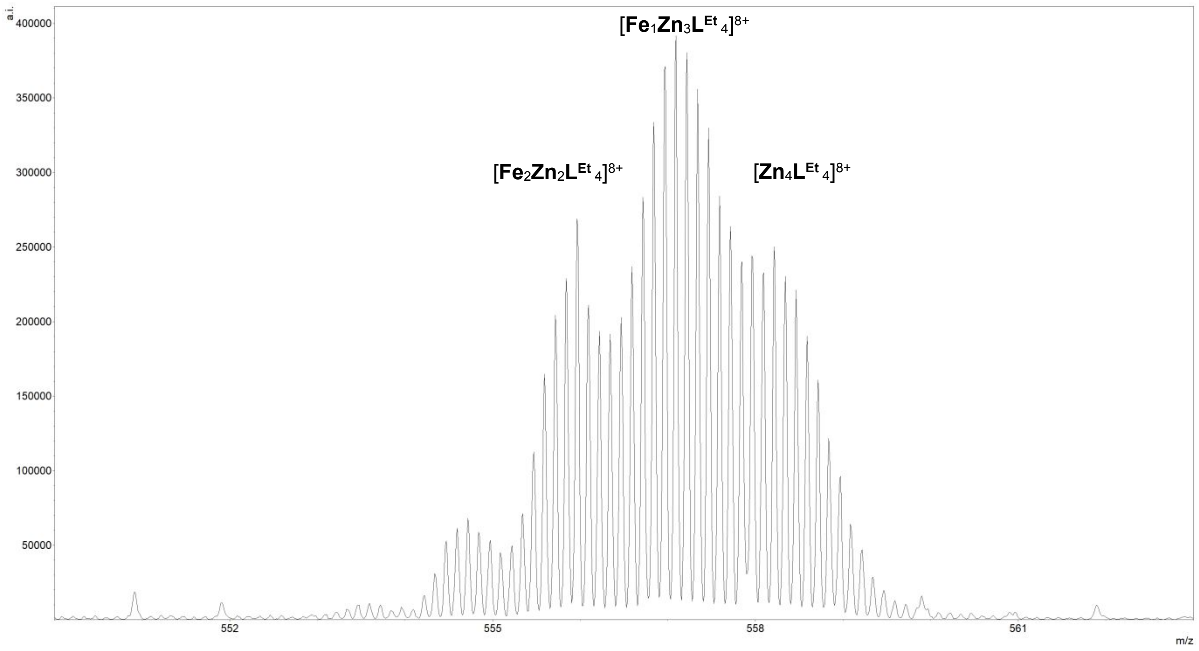

Figure S71. High-resolution mass-spectrum showing the preferential formation of **$Fe_1Zn_3L^{Et}_4$**, starting from **A^{Et}** and a mixture of $Fe(NTf_2)_2$ and $Zn(NTf_2)_2$.

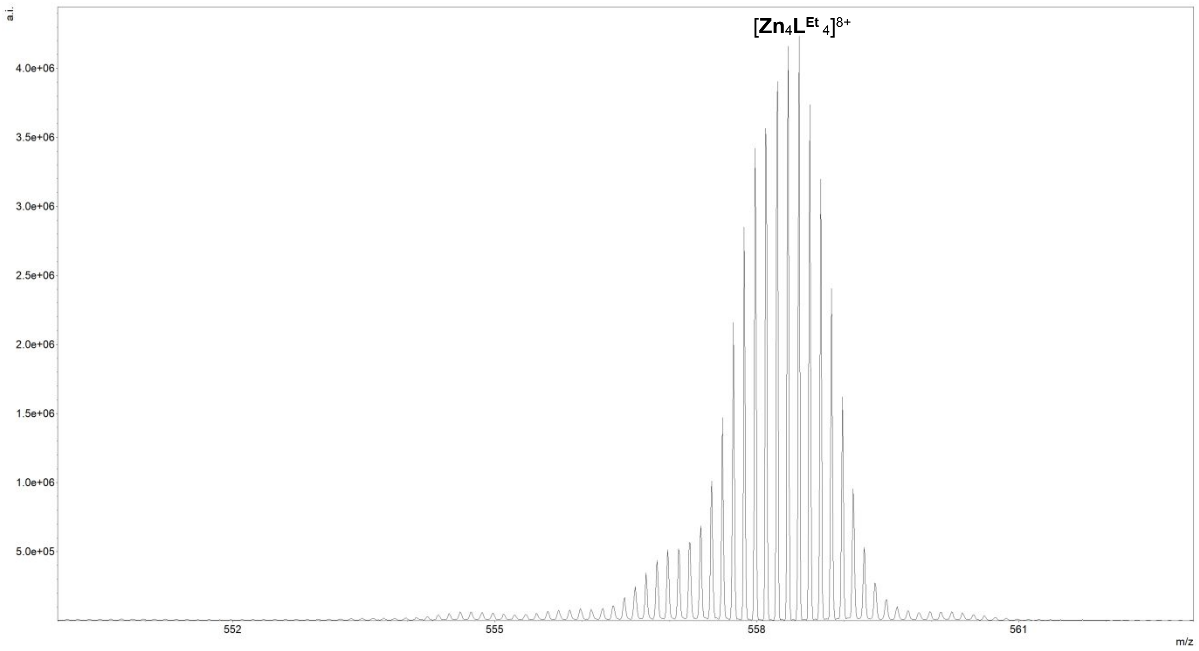

Figure S72. High-resolution mass-spectrum showing the preferential formation of **$Zn_4L^{Et}_4$** upon addition of $Zn(NTf_2)_2$ to **$Fe_4L^{Et}_4$**.

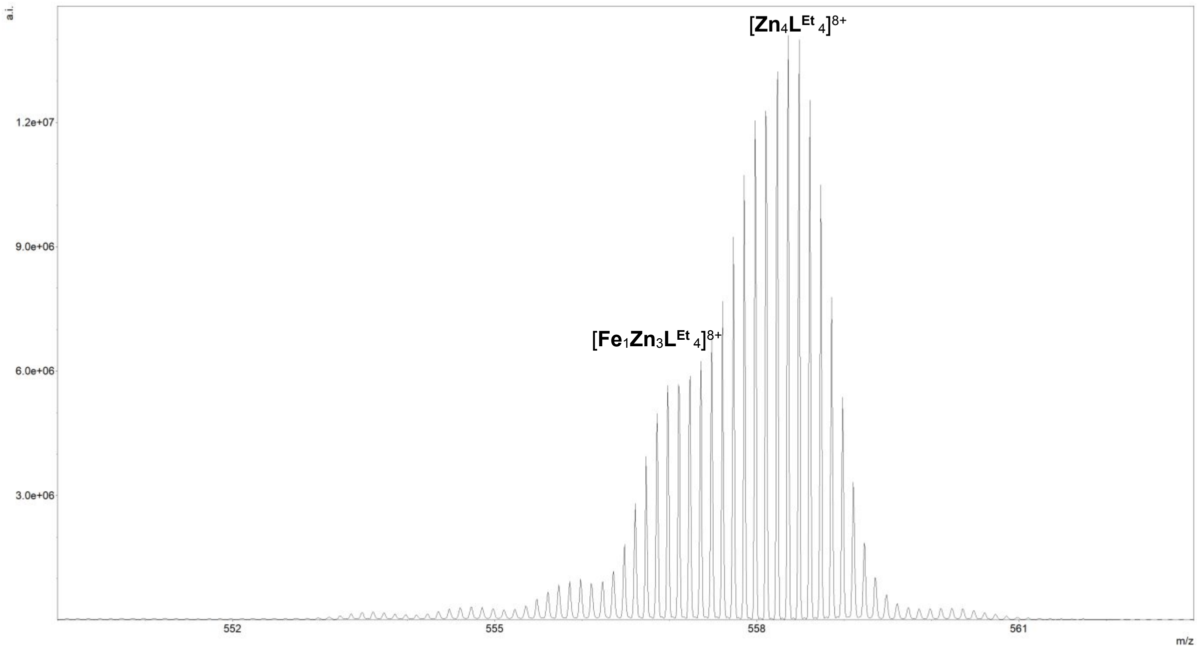

Figure S73. High-resolution mass-spectrum showing the preferential formation of $\mathbf{Zn_4L^{Et}}_4$ and minor amounts of $\mathbf{Fe_1Zn_3L^{Et}}_4$ upon addition of $Fe(NTf_2)_2$ to $\mathbf{Zn_4L^{Et}}_4$.

9. CID-MS experiments

The gradient tandem mass (gMS^2) spectra for cages **$Fe_4L^{Me}_4$**, **$Fe_4L^{Et}_4$**, **$Zn_4L^{Me}_4$** and **$Zn_4L^{Et}_4$** were recorded on a Waters Synapt G2-Si mass spectrometer under the following conditions: Q-isolated ion, 7+; ESI capillary voltage, 2.0 kV; sample cone voltage, 10 V; trap gas flow, 8 mL min^{-1}; source offset, 80 V; source temperature, 80 °C; desolvation temperature, 120 °; cone gas flow, 60 L h^{-1}; desolvation gas flow, 600 L/h. The trap voltage was increased to induce dissociation of each ion (0-15 V for **$Zn_4L^{Me}_4$**, 0-21V for **$Zn_4L^{Et}_4$** and **$Fe_4L^{Me}_4$**, and 0-25 V for **$Fe_4L^{Et}_4$**). The cages were injected as 0.15 μM solutions at a 420 μl/h rate using a syringe pump.

Note that the $[(M_4L_4)(NTf_2)]^{7+}$ ions, rather than $[M_4L_4]^{8+}$, were analyzed because the fragmentation of $[M_4L_4]^{8+}$ yields $[M_2L_2]^{4+}$ as one of the major products, which features the same *m*/*z* ratio as the parent ion. The extent of fragmentation of $[(M_4L_4)(NTf_2)]^{7+}$ into $[M_2L_2]^{4+}$ and $[(M_2L_2)(NTf_2)]^{3+}$ (and additional fragments) is thus simpler to quantify, although the presence of a single anion might introduce a small systematic error (which would be similar for all four cages).

$$SY = \frac{I_p}{I_P + \sum I_f} (4)$$

$$E_{cm} = E_{lab} \cdot \left(\frac{m_{gas}}{m_{gas} + m_{ion}}\right) (5)$$

The survival yield (SY), defined as the intensity of isolated ions (I_p) divided by total ion intensity (I_p + sum of fragment ions (ΣI_f)) (Eq. 4), is plotted against the centre-of-mass collision energy (E_{cm}) (Eq. 5), where E_{lab} is the laboratory-frame kinetic energy, m_{gas} is the mass of the target gas (argon) and m_{ion} is the mass of the isolated ion. As expected, the survival yield decreased in a sigmoidal fashion with increasing collision energy. The data has been fit with Eq. 6.

$$f(x) = \frac{100}{1 + a \cdot e^{bx}} (6)$$

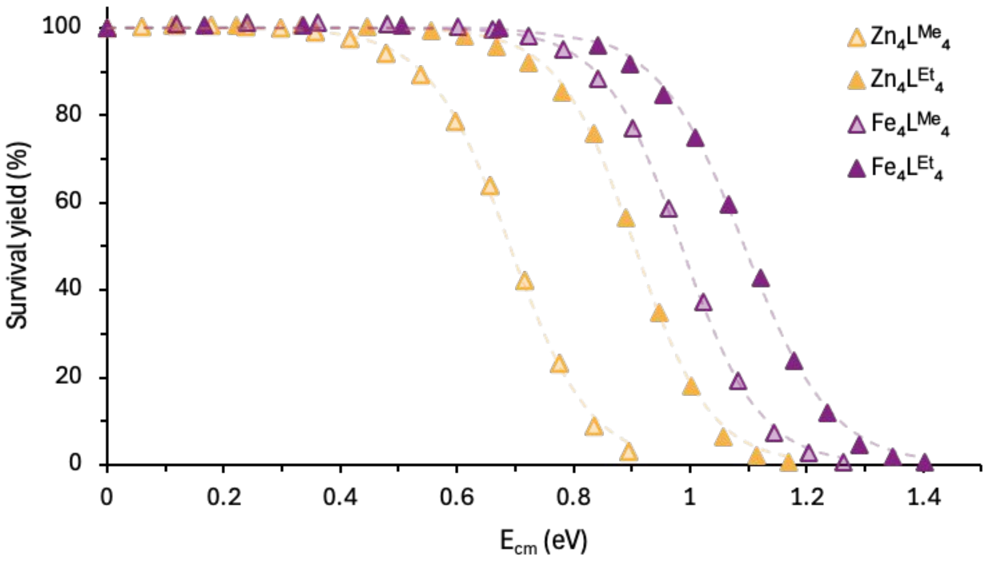

Figure S74. Survival yield curve comparisons between the experimental data and the theoretical sigmoidal curve for the four cages. Structure **$Zn_4L^{Me}_4$** sigmoid survival yield = $100/(1+4.47\times10^{-5}e^{14.5*E_{cm}})$. Structure **$Zn_4L^{Et}_4$** sigmoid survival yield = $100/(1+9.04\times10^{-7}e^{15.4*E_{cm}})$. Structure **$Fe_4L^{Me}_4$** sigmoid survival yield = $100/(1+4.22\times10^{-7}e^{14.9*E_{cm}})$. Structure **$Fe_4L^{Et}_4$** sigmoid survival yield = $100/(1+4.53\times10^{-7}e^{13.4*E_{cm}})$.

Eq. 7 (a linearised form of Eq. 6) produces a linear fit from which the energy required for 50% dissociation (CE_{50}) of the respective ion can be calculated.

$$\ln\left(\frac{100 - SY}{SY}\right) = \ln(a) + bx (7)$$

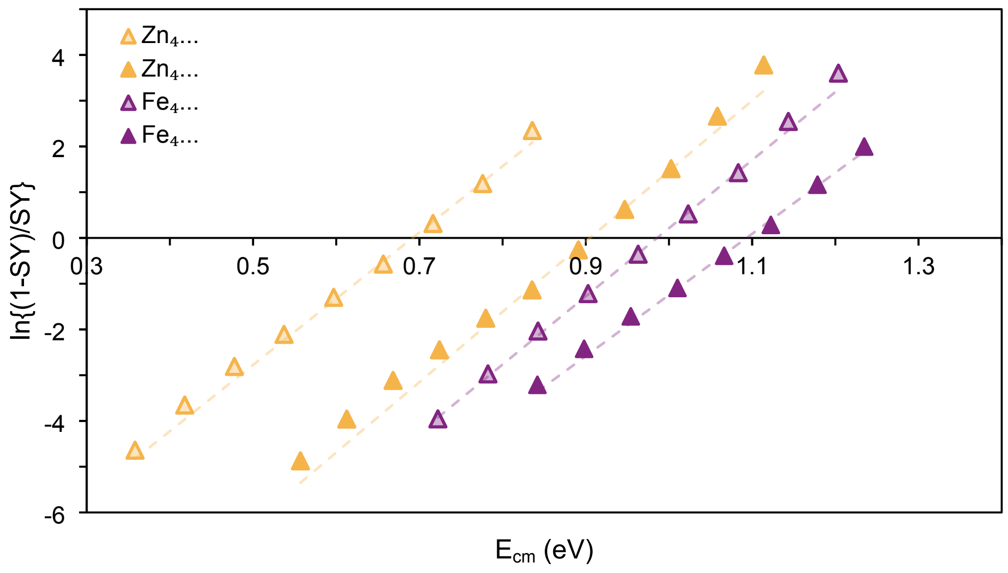

Figure S75. Linearised survival yield plots for the four cages. **$Zn_4L^{Me}{}_4$** CE_{50} = 0.69±0.0006 eV, **$Zn_4L^{Et}{}_4$** CE_{50} = 0.91±0.0003 eV, **$Fe_4L^{Me}{}_4$** CE_{50} = 0.99±0.0048 eV and **$Fe_4L^{Et}{}_4$** CE_{50} = 1.09±0.0023 eV.

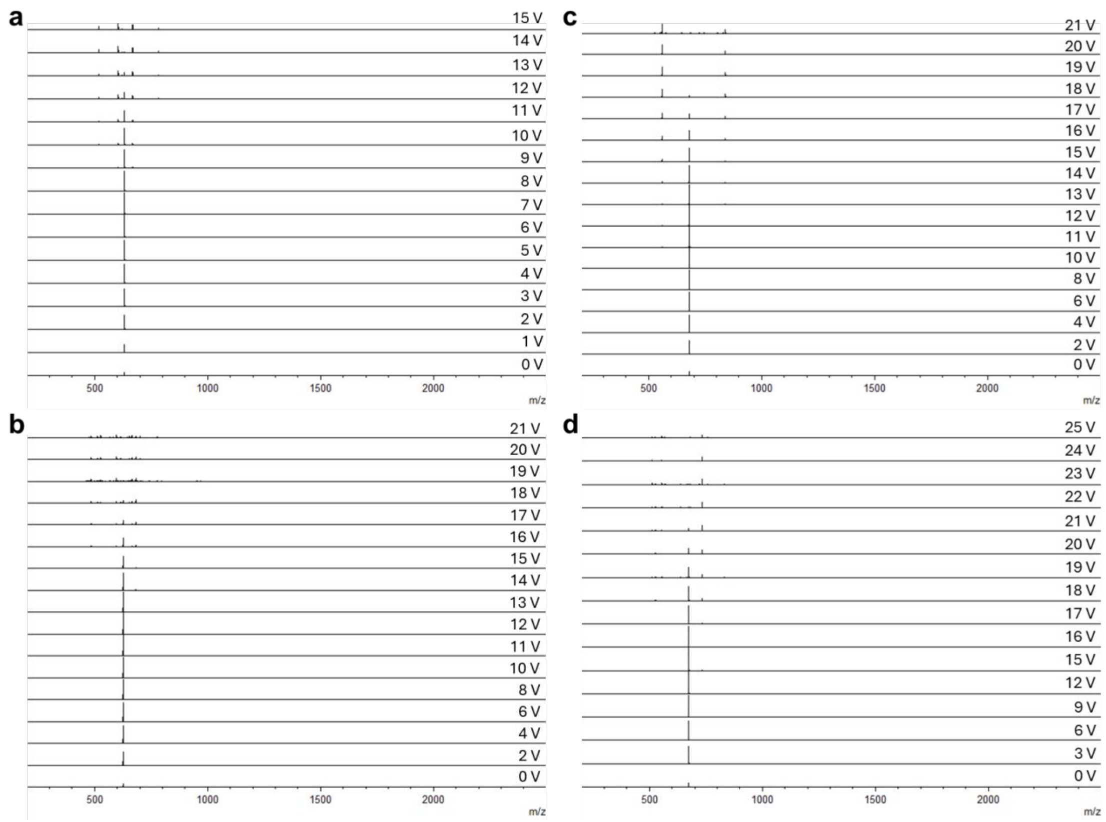

Figure S76. **a.** gMS^2 spectra of **$Zn_4L^{Me}{}_4$**. **b.** gMS^2 spectra of **$Fe_4L^{Me}{}_4$**. **c.** gMS^2 spectra of **$Zn_4L^{Et}{}_4$**. **d.** gMS^2 spectra of **$Fe_4L^{Et}{}_4$**.

10. Self-sorting experiments

From subcomponents and both metal salts

To a vial, **A^{Me}** (1 equiv., 2 mg, 2.9 µmol), **A^{Et}** (1 equiv., 2.2 mg, 2.9 µmol), $Fe(NTf_2)_2$ (92% purity, 1 equiv., 1.9 mg, 2.9 µmol), $Zn(NTf_2)_2$ (93% purity, 1 equiv., 19.2 mg, 28.6 µmol, and 2-formylpyridine (6 equiv., 18 µmol, 20 µl of a stock solution containing 100 µl acetonitrile and 8.3 µl 2-formylpyridine) were added. Acetonitrile (5 ml) was added, and the mixture was stirred at room temperature for 1 hour, until a clear solution formed. 500 µl of the above solution were transferred to a J-Young NMR tube, which was then sealed and heated to 80 °C for 10 days. A 100 µl aliquot was diluted with 100 µl of acetonitrile, filtered on glass fibre, and analysed by ESI-HRMS (Figures S78-79).

From pre-formed cages

To a J-young NMR tube, **$Zn_4L^{Me}_4$** (1 equiv., 0.71 µmol, 100 µl of a 7.1 µM stock solution) and **$Fe_4L^{Et}_4$** (1 equiv., 0.71 µmol, 100 µl of a 7.1 µM stock solution were added). Acetonitrile (300 µl) was added, and the tube was sealed and heated to 80 °C for 10 days. A 100 µl aliquot was diluted with 100 µl of acetonitrile, filtered on glass fibre, and analysed by ESI-HRMS (Figure S80).

Analysis

The product distribution was quantified using an online mass spectrum deconvolution tool,[29] where the 8+ peaks were analysed.

The relative response factors (RRFs) of the different cages were found to differ slightly. The RRFs of the homoleptic-homometallic cages were quantified by injecting a 1/1 mixture of two cages and dividing the integrals of the 8+ peaks (Table S8). Then, the RRFs of the mixed cages in the self-sorting experiments were estimated as weighted averages of the RRFs of the homoleptic-homometallic cages (Table S8).

Table S8. Relative response factors (RRFs) of the different cages in the system, where the lowest value ($Zn_4L^{Me}_4$) was set to 1. Values in bold were measured directly from the relative integrals in the mass spectrum of a freshly prepared 1/1 mixture of the two respective cages.

**RRF**	Fe_4	Fe_3Zn_1	Fe_2Zn_2	Fe_1Zn_3	Zn_4
L^{Me}_4	**1.25**	1.18	1.12	1.06	**1.00**
$L^{Me}_3L^{Et}_1$	1.41	1.36	1.31	1.26	1.21
$L^{Me}_2L^{Et}_2$	1.57	1.53	1.50	1.46	1.42
$L^{Me}_1L^{Et}_3$	1.73	1.71	1.68	1.66	1.63
L^{Et}_4	**1.90**	1.88	1.87	1.85	**1.84**

Both homometallic **$M_4L^{Me}_4$** cages have similar RRFs, smaller than the those of the **$M_4L^{Et}_4$** cages, which are also similar. We attribute the difference in RRFs to partial fragmentation of the more labile Me-containing cages prior to detection (in line with CID-MS studies above), such that the more fragile cages are detected with smaller sensitivity.

The relative peak intensities obtained from deconvolution were divided by the RRFs, giving rise to the distributions displayed as heat maps in Fig. 5 in the paper.

Qualitatively, the results show that the system self-sorts to couple **L^{Me}** with **Fe^{II}**, and **L^{Et}** with **Zn^{II}**, and the product distribution significantly deviates from a purely statistical mixture. Two factors complicated quantitative analysis: the long reaction times required for equilibration, combined with precipitation of cages on the walls of the tube over the course of the reaction. The precipitate, dark in colour, is most likely enriched in **L^{Me}** and **Fe^{II}**, since samples where substantial precipitation was observed only showed **$M_4L^{Et}_4$** and minor amounts of **$M_4L^{Me}_1L^{Et}_3$** by HRMS (Figure S81). This observation is in line with the higher solubility of Et-containing cages observed empirically. The combination of these two factors (slow equilibration and precipitation) prevented the attainment of a converged product distributions between

the two experiments (starting from the mismatched cages ($Zn_4L^{Me}_4$ and $Fe_4L^{Et}_4$) or the individual subcomponents and metal salts), as longer reaction times led to precipitation.

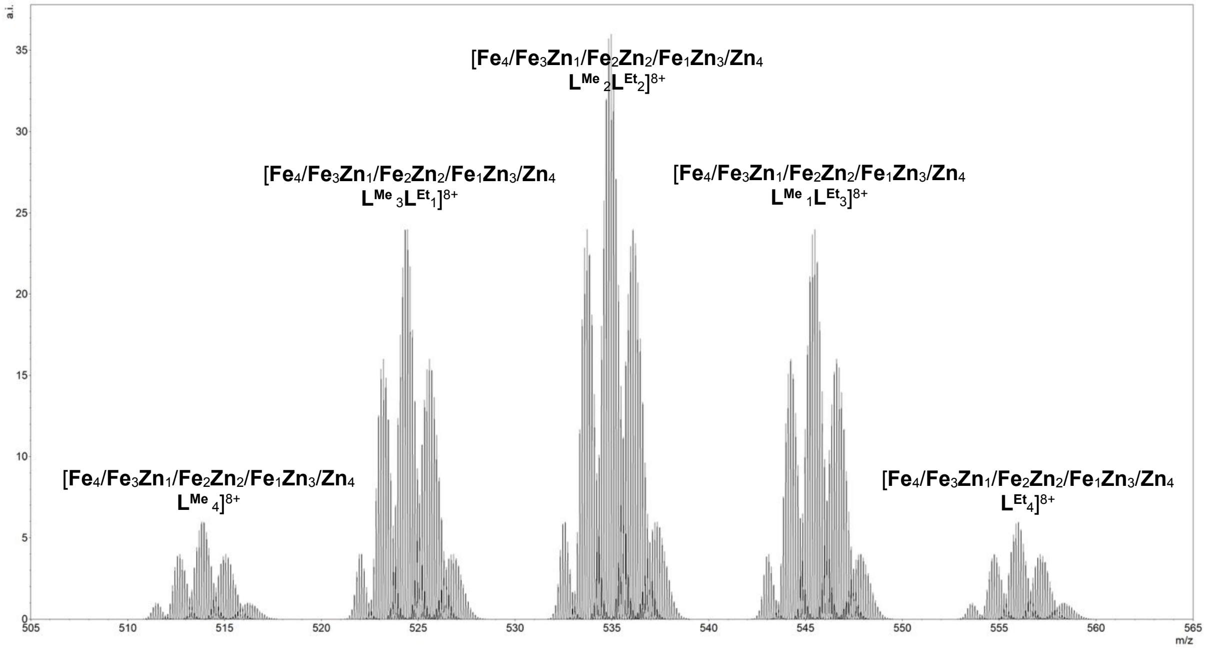

Figure S77. Predicted mass-spectrum peaks of the 8+ cations for the 25 possible cages resulting from mixing Fe^{II}, Zn^{II}, L^{Me}, and L^{Et}, in intensity ratios expected for purely statistical control.

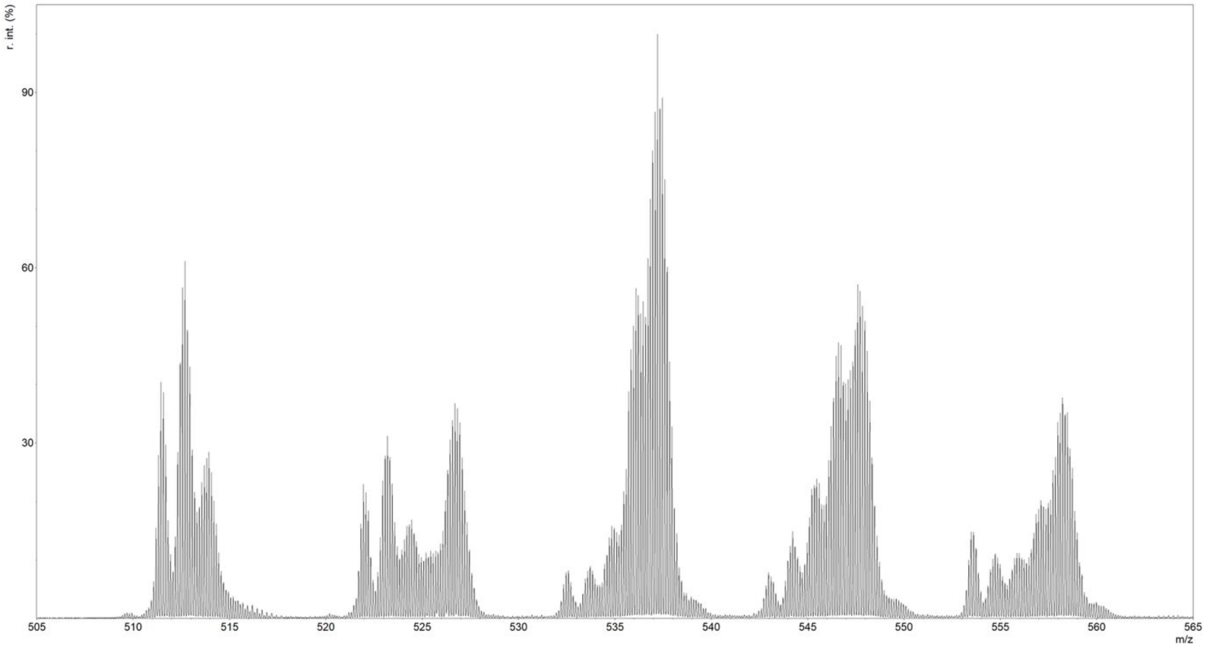

Figure S78. High-resolution mass-spectrum from a self-sorting experiment starting from equal amounts of A^{Me}, A^{Et}, Fe^{II}, and Zn^{II}, after 1 hour at room temperature.

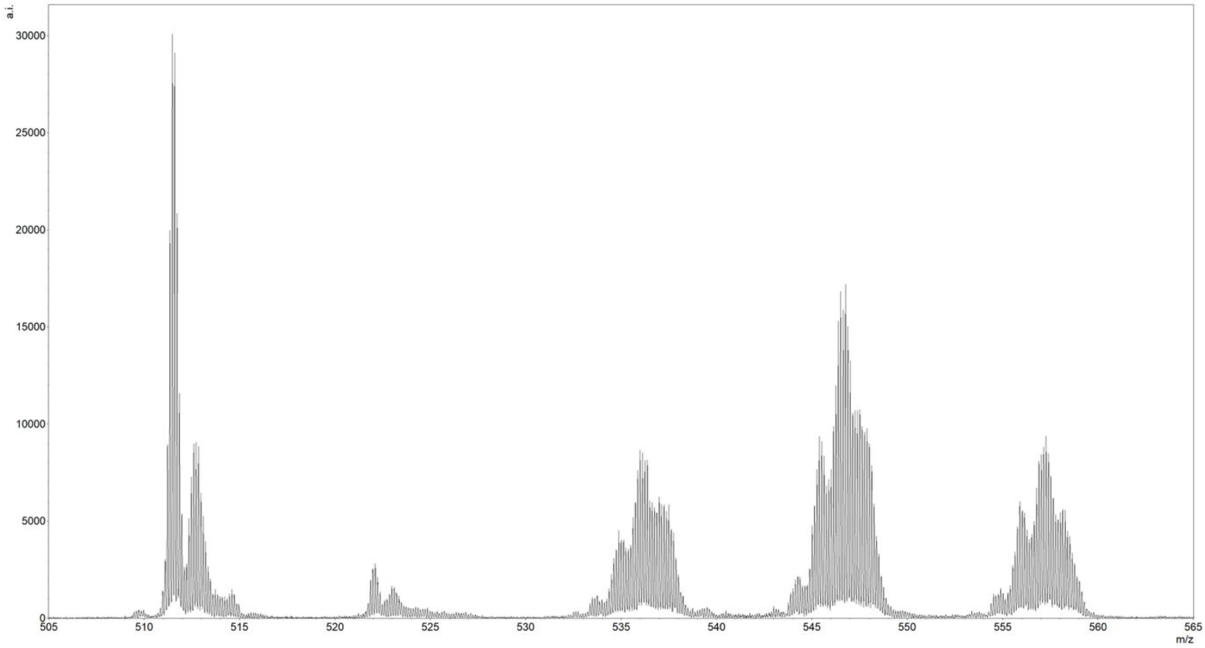

Figure S79. High-resolution mass-spectrum from a self-sorting experiment starting from equal amounts of $\mathbf{A^{Me}}$, $\mathbf{A^{Et}}$, $\mathbf{Fe^{II}}$, and $\mathbf{Zn^{II}}$, after 10 days at 80 °C.

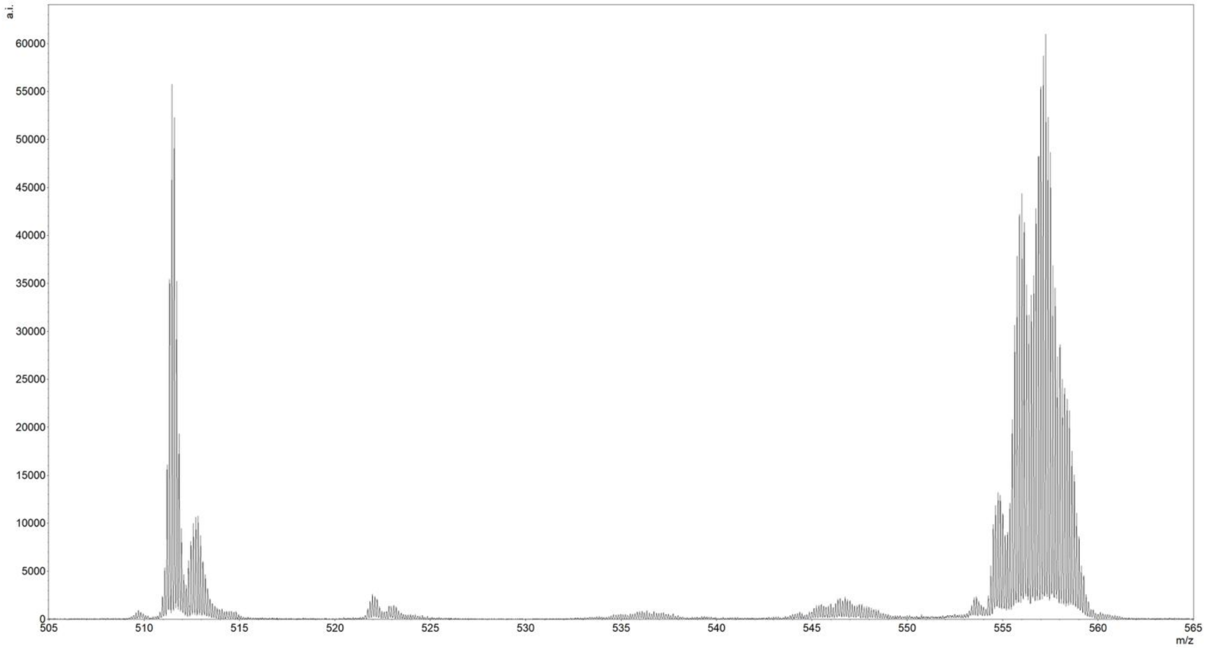

Figure S80. High-resolution mass-spectrum from a self-sorting experiment starting from equal amounts of $\mathbf{Zn_4L^{Me}_4}$ and $\mathbf{Fe_4L^{Et}_4}$ after 10 days at 80 °C.

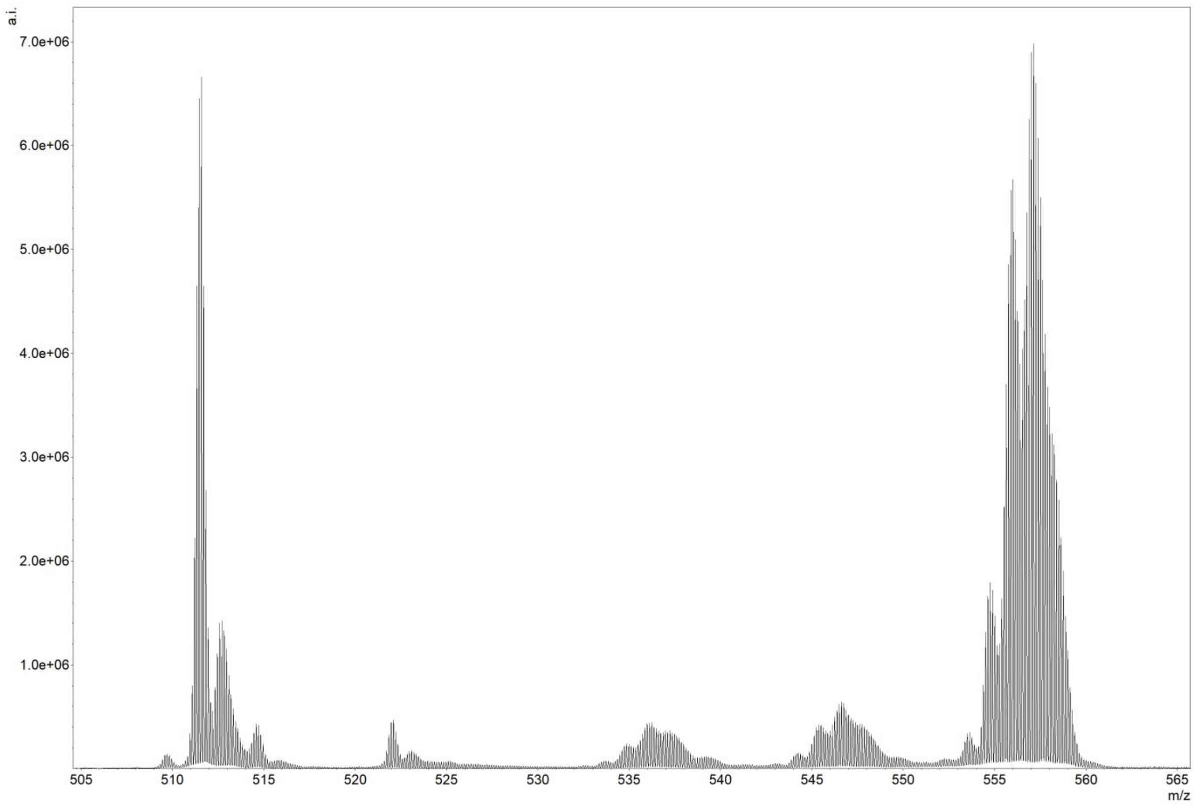

Figure S81. High-resolution mass-spectrum from a self-sorting experiment starting from equal amounts of $\mathbf{Fe_4L^{Me}_4}$ and $\mathbf{Zn_4L^{Et}_4}$ after 10 days at 80 °C.

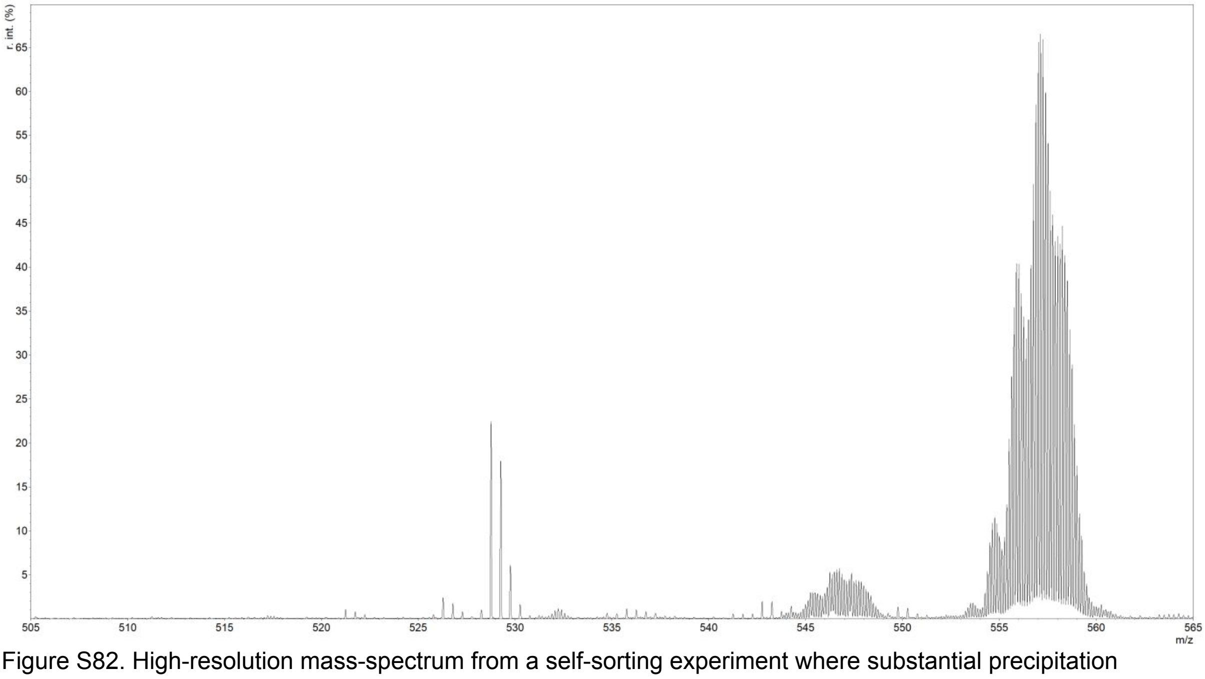

Figure S82. High-resolution mass-spectrum from a self-sorting experiment where substantial precipitation occurred after heating at 80 °C for 2 weeks.

To assess the enthalpic difference between the 25 cages, the relative concentrations from Fig. 5b were divided by the respective binomial coefficient for each cage (shown in Fig. 5b, left). Figure S82 thus shows that $\mathbf{Fe_4L^{Me}_4}$ and $\mathbf{Zn_4L^{Et}_4}$ are enthalpically favoured.

	Fe_4	Fe_3Zn_1	Fe_2Zn_2	Fe_1Zn_3	Zn_4
L^{Me}_4	19	2	0	0	0
$L^{Me}_3L^{Et}_1$	0	0	0	0	0
$L^{Me}_2L^{Et}_2$	0	0	0	0	1
$L^{Me}_1L^{Et}_3$	0	0	0	1	2
L^{Et}_4	0	0	1	2	4

	Fe_4	Fe_3Zn_1	Fe_2Zn_2	Fe_1Zn_3	Zn_4
L^{Me}_4	21	2	0	0	0
$L^{Me}_3L^{Et}_1$	0	0	0	0	0
$L^{Me}_2L^{Et}_2$	0	0	0	0	0
$L^{Me}_1L^{Et}_3$	0	0	0	0	0
L^{Et}_4	1	1	3	7	12

Figure S83. Relative concentration values from the mixed-subcomponents (left) and mixed-cages (right) experiments divided by the respective binomial probability coefficients for each cage.

11. References

(1) Fan, Y.; Wen, Q.; Zhan, T. G.; Qi, Q. Y.; Xu, J. Q.; Zhao, X. A case study on the influence of substitutes on interlayer stacking of 2D covalent organic frameworks. *Chem. Eur. J.* **2017**, *23*, 5668-5672.
(2) Zhu, J.-L.; Zhang, D.; Ronson, T. K.; Wang, W.; Xu, L.; Yang, H.-B.; Nitschke, J. R. A Cavity-Tailored Metal-Organic Cage Entraps Gases Selectively in Solution and the Amorphous Solid State. *Angew. Chem. Int. Ed.* **2021**, *60*, 11789-11792.
(3) McConnell, A. J.; Aitchison, C. M.; Grommet, A. B.; Nitschke, J. R. Subcomponent exchange transforms an FeII4L4 cage from high-to low-spin, switching guest release in a two-cage system. *J. Am. Chem. Soc.* **2017**, *139*, 6294-6297.
(4) Zheng, J.; von Krbek, L. K.; Ronson, T. K.; Nitschke, J. R. Host Spin-Crossover Thermodynamics Indicate Guest Fit. *Angew. Chem. Int. Ed.* **2022**, *61*, e202212634.
(5) Klaeui, W.; Eberspach, W.; Guetlich, P. Spin-crossover cobalt (III) complexes: steric and electronic control of spin state. *Inorg. Chem.* **1987**, *26*, 3977-3982.
(6) Allan, D.; Nowell, H.; Barnett, S.; Warren, M.; Wilcox, A.; Christensen, J.; Saunders, L.; Peach, A.; Hooper, M.; Zaja, L.; et al. A Novel Dual Air-Bearing Fixed-χ Diffractometer for Small-Molecule Single-Crystal X-ray Diffraction on Beamline I19 at Diamond Light Source. *Crystals* **2017**, *7*, 336.
(7) Evans, P. Scaling and assessment of data quality. *Acta Cryst.* **2006**, *D62*, 72-82.
(8) Winter, G. xia2: an expert system for macromolecular crystallography data reduction. *J. Appl. Crystallogr.* **2010**, *43*, 186-190.
(9) Winter, G.; Waterman, D. G.; Parkhurst, J. M.; Brewster, A. S.; Gildea, R. J.; Gerstel, M.; Fuentes-Montero, L.; Vollmar, M.; Michels-Clark, T.; Young, I. D.; et al. DIALS: implementation and evaluation of a new integration package. *Acta Cryst.* **2018**, *D74*, 85-97.
(10) Farrugia, L. WinGX and ORTEP for Windows: an update. *J. Appl. Crystallogr.* **2012**, *45*, 849-854.
(11) Hubschle, C. B.; Sheldrick, G. M.; Dittrich, B. ShelXle: a Qt graphical user interface for SHELXL. *J. Appl. Crystallogr.* **2011**, *44*, 1281-1284.
(12) Sheldrick, G. SHELXT - Integrated space-group and crystal-structure determination. *Acta. Cryst.* **2015**, *A71*, 3-8.
(13) Sheldrick, G. M. Crystal structure refinement with SHELXL. *Acta. Cryst.* **2015**, *C71*, 3-8.
(14) van der Sluis, P.; Spek, A. L. BYPASS: an effective method for the refinement of crystal structures containing disordered solvent regions. *Acta Cryst.* **1990**, *A46*, 194-201.
(15) Spek, A. L. *PLATON: A Multipurpose Crystallographic Tool*; Utrecht University, 2008.
(16) Bolliger, J. L.; Ronson, T. K.; Ogawa, M.; Nitschke, J. R. Solvent effects upon guest binding and dynamics of a FeII4L4 cage. *J. Am. Chem. Soc.* **2014**, *136*, 14545-14553.
(17) Rizzuto, F. J.; Carpenter, J. P.; Nitschke, J. R. Multisite binding of drugs and natural products in an entropically favorable, heteroleptic receptor. *J. Am. Chem. Soc.* **2019**, *141*, 9087-9095.
(18) Maglic, J. B.; Lavendomme, R. MoloVol: an easy-to-use program for analyzing cavities, volumes and surface areas of chemical structures. *J. Appl. Crystallogr.* **2022**, *55*, 1033-1044.
(19) OPTIM: A program for geometry optimisation and pathway calculations. https://www-wales.ch.cam.ac.uk/OPTIM/,
(20) Wesołowski, P. A.; Wales, D. J.; Pracht, P. Multilevel Framework for Analysis of Protein Folding Involving Disulfide Bond Formation. *J. Phys. Chem. B.* **2024**, *128*, 3145-3156.
(21) Bannwarth, C.; Ehlert, S.; Grimme, S. GFN2-xTB—An accurate and broadly parametrized self-consistent tight-binding quantum chemical method with multipole electrostatics and density-dependent dispersion contributions. *J. Chem. Theory. Comput.* **2019**, *15*, 1652-1671.
(22) Bursch, M.; Neugebauer, H.; Grimme, S. Structure optimisation of large transition-metal complexes with extended tight-binding methods. *Angew. Chem. Int. Ed.* **2019**, *58*, 11078-11087.
(23) Bannwarth, C.; Caldeweyher, E.; Ehlert, S.; Hansen, A.; Pracht, P.; Seibert, J.; Spicher, S.; Grimme, S. Extended tight-binding quantum chemistry methods. *Wiley Interdisciplinary Reviews: Computational Molecular Science* **2021**, *11*, e1493.
(24) Ehlert, S.; Stahn, M.; Spicher, S.; Grimme, S. Robust and efficient implicit solvation model for fast semiempirical methods. *J. Chem. Theory. Comput.* **2021**, *17*, 4250-4261.
(25) Neese, F.; Wennmohs, F.; Becker, U.; Riplinger, C. The ORCA quantum chemistry program package. *J. Chem. Phys.* **2020**, *152*,
(26) Grimme, S.; Hansen, A.; Ehlert, S.; Mewes, J.-M. r2SCAN-3c: A “Swiss army knife” composite electronic-structure method. *J. Chem. Phys.* **2021**, *154*,
(27) Weigend, F. Accurate Coulomb-fitting basis sets for H to Rn. *Phys. Chem. Chem. Phys.* **2006**, *8*, 1057-1065.

(28) Carroy, G.; Lemaur, V.; De Winter, J.; Isaacs, L.; De Pauw, E.; Cornil, J.; Gerbaux, P. Energy-resolved collision-induced dissociation of non-covalent ions: charge-and guest-dependence of decomplexation reaction efficiencies. *Phys. Chem. Chem. Phys.* **2016**, *18*, 12557-12568.
(29) Mass spectra deconvolution, https://mstools.epfl.ch/deconvolution/, (2025).
